# Reaction Mechanisms and Kinetics of the Hydrogen Abstraction Reactions of C_4_–C_6_ Alkenes with Hydroxyl Radical: A Theoretical Exploration

**DOI:** 10.3390/ijms20061275

**Published:** 2019-03-14

**Authors:** Quan-De Wang, Mao-Mao Sun, Jin-Hu Liang

**Affiliations:** 1 Low Carbon Energy Institute and School of Chemical Engineering, Jiangsu Province Engineering Lab of High-Efficient Energy Storage Technology and Equipment, China University of Mining and Technology, Xuzhou 221008, China; maomaosun2019@126.com; 2 School of Environment and Safety Engineering, North University of China, Taiyuan 030051, China

**Keywords:** abstraction reactions, reaction mechanism, transition state theory

## Abstract

The reaction of alkenes with hydroxyl (OH) radical is of great importance to atmospheric and combustion chemistry. This work used a combined ab initio/transition state theory (TST) method to study the reaction mechanisms and kinetics for hydrogen abstraction reactions by OH radical on C_4_–C_6_ alkenes. The elementary abstraction reactions involved were divided into 10 reaction classes depending upon the type of carbon atoms in the reaction center. Geometry optimization was performed by using DFT M06-2X functional with the 6-311+G(d,p) basis set. The energies were computed at the high-level CCSD(T)/CBS level of theory. Linear correlation for the computed reaction barriers and enthalpies between M06-2X/6-311+G(d,p) and CCSD(T)/CBS methods were found. It was shown that the C=C double bond in long alkenes not only affected the related allylic reaction site, but also exhibited a large influence on the reaction sites nearby the allylic site due to steric effects. TST in conjunction with tunneling effects were employed to determine high-pressure limit rate constants of these abstraction reactions and the computed overall rate constants were compared with the available literature data.

## 1. Introduction

The reaction of alkenes with hydroxyl (OH) radical is of great importance to atmospheric chemistry and combustion chemistry [1,2]. The development of detailed chemical kinetic mechanisms used for atmospheric and combustion kinetic modeling requires a detailed understanding of the reaction mechanisms and kinetics for the reaction of alkenes with OH. Generally, the reaction of alkenes with OH can proceed through two pathways—the addition reaction channel of OH to the C=C double bond in alkenes and the abstraction reaction channel of hydrogen atoms in different reaction sites in alkenes [2,3]. For long alkene molecules, although the abstraction reaction channel at the vinylic site cannot compete with the addition channel at the C=C double bond position, the abstraction channels at the other reaction sites are dominant in the overall rate of reaction, especially at high temperatures. Thus, extensive work has been conducted in studying the reaction of alkenes with OH, especially in combustion chemistry [4,5,6,7,8,9,10].

With advanced experimental facilitates and the development of theoretical chemistry methods, accurate rate constants are available for small alkenes including ethylene and propene [4,5]. However, for large alkenes with OH, the kinetics becomes very complex during the development of detailed chemical kinetic models due to the different reaction types and reaction sites. Hence, reaction classes (RC) are generally employed to describe these processes, especially for the fuel oxidation process [11]. Butene is usually selected as the first typical alkene toward large alkene molecules due to the associated isomers which can represent the influence of the location and structure of the C=C double bond in long alkenes [6]. Detailed reaction mechanisms for modeling the combustion of butene isomers have been validated, and the success of the reaction classes are then adopted for large alkenes [2,7,8]. In order to identify the key reactions which greatly affect modeling results, sensitivity and reaction path analysis are employed. It has been found that hydrogen abstraction reactions are key initiation steps and are crucial in the prediction of combustion properties [6,7,8]. Recently, a shock tube facility was employed to study the reactions of butene isomers and C_5_–C_6_ straight chain alkenes with OH [9,10,12]. The overall reaction rate coefficients were measured and fitted. For atmospheric chemistry, the low temperature reaction mechanisms were also studied. McGillen et al. [3] investigated the reactions of large alkenes with OH and they concluded that the abstraction pathway also makes an appreciable contribution to the overall reaction rate. Further, the reactions of alkenes with OH were pressure-independent and were at their high-pressure limit. However, it was very hard to extract reaction rate constants for elementary reactions used in chemical kinetic modeling studies. Further, it was shown that the location of C=C double bond in long alkenes exhibited some unexpected trends among different structural alkenes [10], indicating that the C=C double bond may also induce steric effects compared with small alkenes. This unexpected trend suggested that the rate constants of reaction classes for small alkenes may be improper for large alkenes. Therefore, to continuously improve the accuracy of detailed reaction mechanisms for atmospheric and combustion chemistry, systematic theoretical studies of these reaction classes for long alkenes are crucial.

Based on the above considerations, detailed theoretical studies for hydrogen abstraction reactions of C_4_–C_6_ alkenes with OH radical are presented in this work. A comprehensive set of reactions of different structural C_4_–C_6_ alkene molecules are considered. The elementary reactions involved are divided into 10 reaction classes, depending upon the type of carbon atoms in the reaction centers. Conventional transition state theory (TST), together with tunneling effects, are used to compute the high-pressure limit rate constants in the temperature range of 500–2500 K and the overall rate constants are compared with literature data.

## 2. Results and Discussion

### 2.1. Geometry Analysis

**Figure 1:**
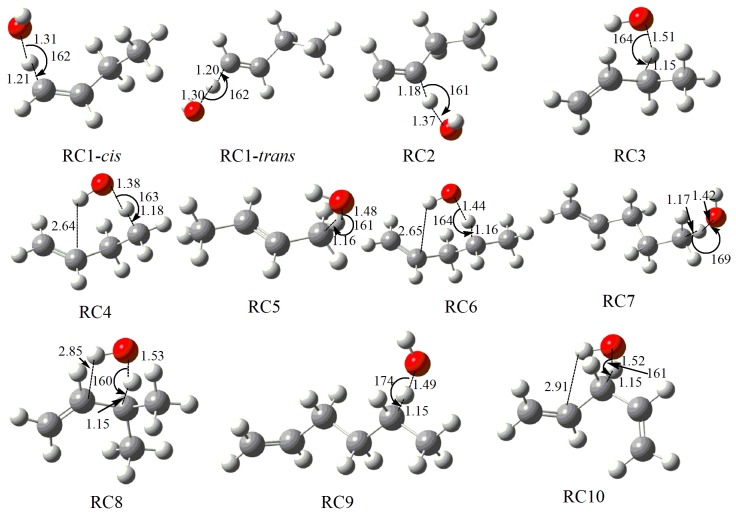
Optimized transition state (TS) structures for the 10 prototype reactions at the M06-2X/6-311+G(d,p) level.

The alkene molecules studied include isomers of butene, pentene, hexene and 1,4-pentadiene. Considering all possible abstraction reaction sites on these alkenes, there are 47 elementary abstraction reactions. These reactions were divided into 10 reaction classes based on the type of carbon atoms in the reaction centers and the RC-TST [13,14]. A full list of all elementary reactions is provided in the Supplementary Materials. The validation of the classification of reaction classes is based on the abstraction reaction centers of the TS structures, which is originally defined in RC-TST. The optimized reaction centers in the geometries of TS structures of different reactions within the same reaction class are compared and provided in the Supplementary Materials. It was found that they share similar TS structures, and the reaction centers of TS structures are nearly identical, indicating that the classification of the reaction classes is reasonable. Figure 1 illustrates the optimized geometries of the TS structures of the prototype reactions for the 10 divided reaction classes at the M06-2X/6-311+G(d,p) level, in which the 10 reaction classes are defined as RC1–RC10, respectively. The local minima corresponding to TSs at certain reaction sites with different hydrogen atoms is confirmed by the relaxed scan, but with the bond lengths frozen at the critical geometries. Only the lowest energy TS structures are considered and shown in Figure 1, while the other modes are treated as hindered rotors. There are *cis* and *trans* configurations for the abstraction reactions at the primary vinylic carbon site for 1-alkenes due to the location of the C=C double bond, which are defined as RC1-*cis* and RC1-*trans*, respectively. For simplicity, we use RC to represent the prototype reactions as shown in Figure 1 unless otherwise specified. For the prototype reactions at the primary vinylic carbon site, namely, RC1-*cis* and RC1-*trans*, the TS structures are nearly identical and only differ slightly from that of RC2 at the secondary vinylic carbon site. The abstraction reactions at the secondary allylic carbon site (RC3), the primary allylic carbon site (RC5), the tertiary allylic carbon site (RC8) and the super secondary allylic carbon site (RC10) also share a similar TS structure. Due to the interaction between the O atom in the OH radical and vinylic C atom, the TS structures for reactions RC4 and RC6 are different from reactions RC7 and RC9, respectively. This also greatly affects the reaction barriers and rate constants as shown below. Thus, it can be concluded that the C=C double bond in large alkenes not only affects the structures and energies of the allylic site, but also has a large influence on the reaction sites near the allylic site.

### 2.2. Reaction Barriers and Enthalpies

The computed reaction barriers (ΔE) and enthalpies ($\Delta_{f}H_{0K}^{o}$) for the 10 prototype abstraction reaction classes for alkenes with OH radical studied at the high CCSD(T)-MP2/CBS level together with the reaction barriers for RC1 to RC4 at the CCSD(T)/CBS level are listed in Table 1. During CCSD(T) calculations, the T1 diagnostic [15] values for reactant species and TSs were between 0.010 and 0.025, which indicates that the use of single-reference methods can describe the wave function appropriately. To validate the computational methods, the abstraction reactions of OH with ethylene (C_2_H_4_) and propene (C_3_H_6_) were also calculated. Details are provided in the Supplementary Materials. The computed barrier for C_2_H_4_ with OH at the CCSD(T)-MP2/CBS//M06-2X/6-311+G(d,p) level was 5.49 kcal/mol, which is very close to 5.6 and 5.4 kcal/mol at the QCISD(T)/6-311+G(2df,2p)//QCISD/6-31G(d,p) and G2//QCISD/6-31G(d,p) levels reported by Liu et al. [16]. For propene with OH, the two abstraction reactions at the vinylic sites were in good agreement with the results obtained by Zador et al. [17]. The result given by the method used in this work (1.73 kcal/mol) was also in reasonable agreement with that of Huyhn et al. (1.60 kcal/mol) [18] and the benchmark results of Szori et al. [19] at the multi-reference [5,5]-CASPT2/CBS//[5,5]-CASPT2/cc-pVTZ level with a value of 1.10 kcal/mol. Thus, this confirms that the computational methods are reasonable for the studied reaction systems. Table 1 also lists the computed reaction barriers for the abstraction reactions of OH with 1-butene at the CCSD(T)/6-311++G(d,p)//BH&HLYP/6-311G(d,p) and CCSD(T)/6-311++G(d,p)//QCISD/6-31G(d) levels reported by Sun et al. [20] and Vasu et al. [9], respectively. The small 6-311++G(d,p) basis set used for CCSD(T) calculations tended to overestimate the reaction barriers for RC1, RC2 and RC4 by an average of 1.08 kcal/mol and to underestimate the reaction barrier for RC3 by an average of 0.43 kcal/mol compared with the present CCSD(T)-MP2/CBS results.

**Figure 2:**
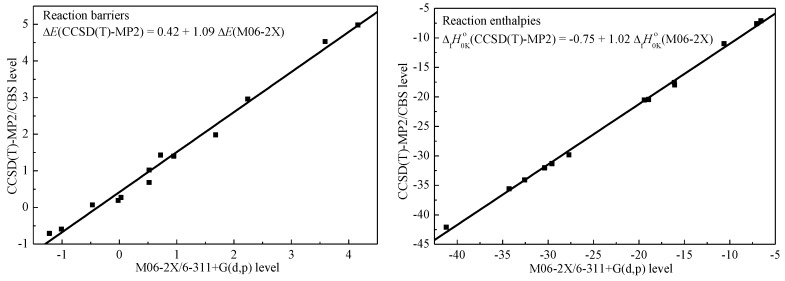
Linear correlation of the computed reaction barriers and enthalpies between CCSD(T)-MP2/CBS and M06-2X/6-311+G(d,p) methods.

**Figure 3:**
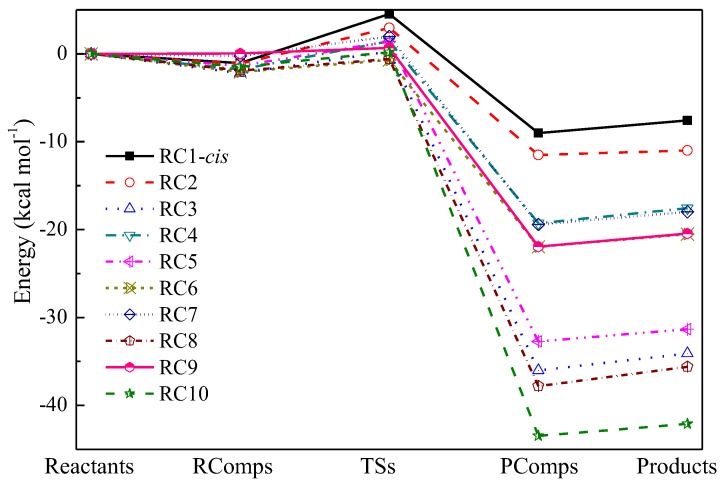
PESs of the abstraction reactions of the 10 prototype reactions at the CCSD(T)-MP2/CBS level.

From Table 1, the deviations of the predicted ΔE and $\Delta_{f}H_{0K}^{o}$ between CCSD(T)/CBS and CCSD(T)-MP2/CBS were within 0.20 and 0.15 kcal/mol, respectively, indicating that the CCSD(T)-MP2/CBS method is a reasonable approximation of the CCSD(T)/CBS method. The deviations of the predicted ΔE and $\Delta_{f}H_{0K}^{o}$ between M06-2X/6-311+G(d,p) and CCSD(T)-MP2/CBS were averages of 0.50 and 1.17 kcal/mol, with the largest deviations being 0.94 and 1.91 kcal/mol, respectively. Although no linear energy relationship like the well-known Evans–Polanyi-like relationship between the reaction barriers and enthalpies was found, a good linear correlation for the computed reaction barriers and enthalpies between M06-2X/6-311+G(d,p) and CCSD(T)-MP2/CBS methods were found and are shown in Figure 2. The absolute averaged deviations of the fitted ΔE and $\Delta_{f}H_{0K}^{o}$ results were reduced to 0.16 and 0.44 kcal/mol compared with the CCSD(T)-MP2/CBS results. Such linear correlation expressions can be helpful to quickly estimate accurate energy information of abstraction reactions for large alkene molecules with OH radical. As shown in Table 1, the studied abstraction reactions were all found to be exothermic with negative $\Delta_{f}H_{0K}^{o}$ values. The abstraction reactions at the vinylic sites were most difficult due to the high reaction barriers, and the reaction barrier at the secondary vinylic reaction site (RC2) was lower than that at the primary vinylic site (RC1) by 1.57 and 2.02 kcal/mol, corresponding to the *cis* and *trans* configurations at the CCSD(T)-MP2/CBS level, respectively. For the reactions at the three allylic sites, both the reaction barriers and reaction enthalpies at the tertiary allylic carbon site (RC8) were the lowest, followed by the secondary allylic carbon site (RC3), and the primary allylic carbon site (RC5) was the largest. As shown in Figure 1, due to the interaction of the O atom in OH radical with the vinylic C atom, the reaction barriers for RC4 and RC6 exhibited large differences from RC7 and RC9 as a result of the TS geometries. The C=C double bond also reduced the reaction barriers of the abstraction reactions near the allylic carbon. However, the reaction enthalpies $\Delta_{f}H_{0K}^{o}$ defined as the energy difference between the products and reactants were not affected, and they were close to each other. This also explains why no Evans–Polanyi-like relationship between the reaction barriers and enthalpies existed. Further, it is also notable that although the abstraction reaction at the primary allylic site (RC5) was much more favorable in thermodynamics compared with that at the primary carbon site (RC4), the two RCs could compete in kinetics due to the close reaction barriers. The carbon site that is two carbon sites away from the C=C double bond was not influenced and the secondary carbon site (RC9) exhibited general reactivity that was faster than the primary carbon site (RC7). In comparison with the abstraction reaction at the secondary allylic carbon site (RC3), the abstraction reaction barrier at the super secondary allylic carbon site in 1,4-pentadiene (RC10) was only reduced by 0.08 kcal/mol at the CCSD(T)-MP2/CBS level, even though it was much more favorable in thermodynamics. Intrinsic reaction coordinate (IRC) results were employed to determine the geometries of reactant complexes (RComps) and product complexes (PComps). The whole potential energy surfaces (PESs) were then further refined at the CCSD(T)-MP2/CBS level as displayed in Figure 3. It was found that the RComps have relative energies in the narrow range from −2.20 to 0.05 kcal/mol compared to the reactants, while those of PComps were consistently lower than those of the products by an average of 1.50 kcal/mol. In addition, the diffusion function and basis set superposition error (BSSE) for the RComp and PComp of RC3 were also examined by comparing results with large cc-pVTZ and aug-cc-pVTZ basis sets. It was demonstrated that the optimized geometries via the 6-311+G(d,p) basis set were very close to the results via the aug-cc-pVTZ basis set. The BSSE corrections were within 0.45 and 0.20 kcal/mol for the 6-311+G(d,p) and aug-cc-pVTZ basis sets. However, the optimized geometries and BSSE corrections via the cc-pVTZ basis set without diffusion function tended to exhibit minor deviations, but the BSSE was also within 1 kcal/mol. Thus, it may be necessary to include the diffusion function in the basis set to determine the formed reactant and product complexes, and a medium 6-311+G(d,p) basis set is reasonable.

To investigate the structural effect of alkenes on the abstraction reactions, the reactions within the same reaction class were analyzed. The averaged values, standard error of mean, and standard deviation of the computed reaction barriers at M06-2X/6-311+G(d,p) and the corrected barriers using linear correlations are provided as Supplementary Materials. For RC1-*cis*, RC1-*trans*, RC2, RC3 and RC4, the averaged corrected reaction barriers were 4.41, 4.76, 2.49, 0.04 and 1.06 kcal/mol associated with the standard error of mean being 0.09, 0.15, 0.09, 0.10 and 0.04, respectively. The deviations within these reaction classes were small—less than 0.5 kcal/mol. For RC5, reaction barriers at the three primary allylic carbon sites of the most branched alkenes, namely, 2-methyl-2-butene (HC(CH_3_)=C(CH_3_)_2_), were the lowest with around 0.5 kcal/mol, followed by that of isobutene, whose barrier was predicted to be 0.99 kcal/mol. The barriers of the other reactions within RC5 lie between 1.25 and 1.50 kcal/mol. The overall averaged reactivity tendency of the studied reaction classes is in accordance with the prototype reactions except for RC3 compared with RC10 due to the slight deviations of the reactions within RC3.

### 2.3. High-Pressure Limit Rate Constants

As shown before, the studied reactions involved the formation of RComps and PComps. To this end, it was necessary to include the contribution of RComps and PComps to the rate constant. However, a series of previous theoretical studies indicated that the formation of the RComps was not the rate-determining step [21,22,23]. Such a simplification is rational in kinetics because the energies of the formed weakly RComps are just lower than the reactants, as shown in Figure 3, and are rather unstable at high temperatures. Hence, it is fairly reasonable to employ the TST method regardless of the RComps to estimate the high-pressure limit rate constants.

**Figure 4:**
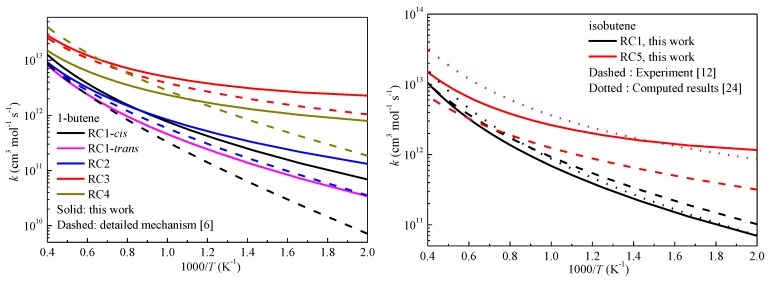
Comparisons of the calculated rate constants of some reactions.

**Figure 5:**
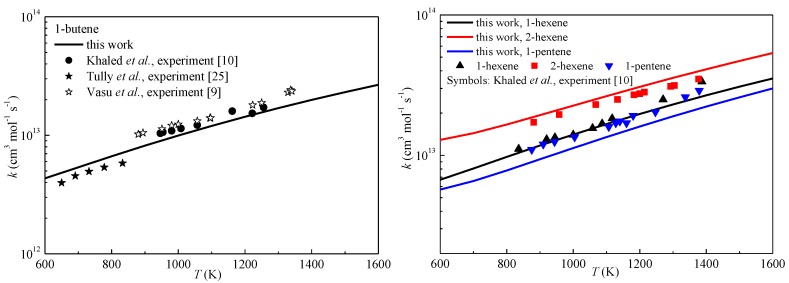
Predicted overall rate constants for some alkenes compared with experiments.

Figure 4 compares the rate constants of some reactions studied in this work at the CCSD(T)-MP2/CBS level with previous rate constants [6,12,24]. It can be seen that the rate constants in this work and those used in the detailed mechanism [7] for 1-butene reveal the same reactivities of the abstraction reactions. The abstraction from the allylic site was the fastest, followed by the primary carbon site and the secondary vinylic site, with the primary vinylic site being the slowest. The computed rate constants of RC1 for isobutene showed good correlation with previous results, while the rate constants of RC5 were much closer to the computed results compared with the experimental results. To further validate our computed results, the overall reactivities of some alkenes with experimental results were compared as shown in Figure 5 by summing contributions from all abstraction sites, assuming that neither mixing nor crossover between different abstraction channel pathways occurred. It can be seen that the computed results correctly exhibited the overall reactivity of the studied alkenes. Specifically, the overall rate constants for 1-butene in this work showed good correlation with previous experimental results over a wide range of temperatures [9,10,25]. For 1-pentene, 1-hexene and 2-hexene, it can be seen that the present results correctly revealed the overall reactivity of these long alkenes. The overall reactivity of 2-hexene was larger than that of 1-hexene due to the two allylic reaction sites. The deviation between 1-pentene and 1-hexene was smaller compared with that of 1-hexene and 2-hexene. This trend was successfully reproduced in this work.

**Figure 6:**
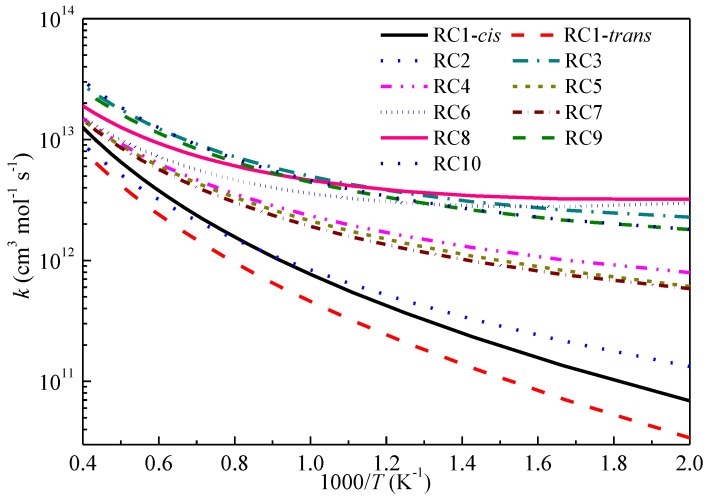
Predicted reaction rate constants for the studied reaction classes.

Figure 6 displays the rate constants as a function of temperature for the studied prototype reactions at the CCSD(T)-MP2/CBS//M06-2X/6-311+G(d,p) level. It is shown that the abstraction reactions at the vinylic sites are most difficult and the *cis* and *trans* configurations of RC1 affect the rate constants. The rate constants at the secondary vinylic site (RC2) are faster than those that at the primary vinylic site (RC1). The rate constants at the primary carbon site nearby the allylic site (RC4), the primary allylic site (RC5) and the primary carbon site (RC7) are close to each other and the rate constants of RC4 are slightly higher than those of the other reaction classes. Hence, the abstraction reactions at these sites can compete with each other in kinetics. For the studied reaction classes at different allylic sites, it can be seen that the rate constants at the tertiary allylic site (RC8), secondary allylic site (RC3), and the super secondary allylic site (RC10) are much larger than that at the primary allylic site (RC5), and the differences among reactions RC8, RC3 and RC10 are not large. The rate constants of RC6 at the secondary carbon site, which is nearby the secondary allylic site, are lower than that of RC3 at temperatures above 800 K and the rate constants of the two reactions grow close as the temperature decreases. A detailed comparison of the elementary reactions within certain reaction classes is shown in the Supplementary Materials, and it was found that the rate constants of the studied reactions for RC1, RC2 and RC4 exhibited small deviations, while in those for RC3, RC5, RC6 and RC7, the branching and length of the alkenes and the location of the C=C double bond still affected the computed rate constants.

## 3. Computational Methods

Geometries of all species were optimized by using the M06-2X functional with the 6-311+G(d,p) basis set [26]. Analytical harmonic frequency calculations were carried out to obtain the zero-point vibrational energies (ZPVEs). All frequencies were scaled by 0.98, with ZPVEs scaled by 0.97 for the M06-2X functional [26]. Intrinsic reaction coordinate (IRC) calculations [27] were performed at the M06-2X/6-311+G(d,p) level to confirm that the TSs were the right saddle point connecting the desired reactants and products. We employed the IRC results to determine the geometries of reactant complexes and product complexes formed in the entrance and exit channels. To obtain reliable potential energies, single point energies (SPEs) were computed by using the coupled cluster (CCSD(T)) method in conjunction with the complete basis set (CBS) limit extrapolated from Dunning’s correlation consistent basis sets [28] for the 10 prototype reactions. Specifically, for the reactions of 1-butene with OH, the SPEs were extrapolated to the CBS limit via the following formula [29]:

$$E_{\mathrm{CCSD}\left(T\right)/\mathrm{CBS}}=E_{\mathrm{CCSD}\left(T\right)/\mathrm{QZ}}+\left(E_{\mathrm{CCSD}\left(T\right)/\mathrm{QZ}}-E_{\mathrm{CCSD}\left(T\right)/\mathrm{TZ}}\right)\times \frac{4^{4}}{5^{4}-4^{4}}$$

where TZ and QZ represent the cc-pVTZ and cc-pVQZ basis sets, respectively. For larger systems, the CCSD(T)/cc-pVQZ calculations were computationally prohibitive. Instead, the SPEs were computed based on extrapolations from the cc-pVDZ and cc-pVTZ results together with the corrected energies for the differences in cc-pVDZ, cc-pVTZ and cc-pVTZ, cc-pVQZ at the MP2 level. The final energies were calculated using the following equation [29]:

$$E_{\mathrm{CCSD}\left(T\right)/\mathrm{CBS}}=E_{\mathrm{CCSD}\left(T\right)/\mathrm{TZ}}+\left(E_{\mathrm{CCSD}\left(T\right)/\mathrm{TZ}}-E_{\mathrm{CCSD}\left(T\right)/\mathrm{DZ}}\right)\times \frac{3^{4}}{4^{4}-3^{4}}+E_{\mathrm{MP}2/\mathrm{QZ}}+\left(E_{\mathrm{MP}2/\mathrm{QZ}}-E_{\mathrm{MP}2/\mathrm{TZ}}\right)\times \frac{4^{4}}{5^{4}-4^{4}}-E_{\mathrm{MP}2/\mathrm{TZ}}-\left(E_{\mathrm{MP}2/\mathrm{TZ}}-E_{\mathrm{MP}2/\mathrm{DZ}}\right)\times \frac{3^{4}}{4^{4}-3^{4}}$$

This method is denoted as CCSD(T)-MP2/CBS in this work. All quantum chemical calculations were performed using Gaussian 09 software [30].

High-pressure limit rate constants from TST were computed via KiSThelP software [31]. Quantum tunneling was computed via the Winger method [32]. One-dimensional (1-D) hindered rotor approximation was considered for low-frequency torsional modes [33]. The hindrance potentials were obtained via the relaxed scan by a dihedral angle step of 10° at the M06-2X/6-311+G(d,p) level. The rate constants were calculated at temperatures from 500 to 2500 K in increments of 100 K, and the data were fitted to the modified Arrhenius expression, $k\left(T\right)=AT^{n}\exp\left(-E_{a}/RT\right)$, in which *A* is the Arrhenius prefactor and *E*_a_ is the barrier height.

## 4. Conclusions

The reaction mechanisms and kinetics were studied by using ab initio calculations together with TST. The optimized geometries for the transition states indicate that the C=C double bond greatly affects the structures of the abstraction sites due to the weak van der Waals interactions between OH radical and C atoms in alkenes. Such an interaction causes steric effects on the abstraction reaction sites not only at the allylic site but also the nearby sites for long alkenes, and the reaction barriers are decreased. Further, it was found that linear correlations exist for the computed reaction barriers and enthalpies between M06-2X/6-311+G(d,p) and high-level CCSD(T)-MP2/CBS methods. Such correlations provide an efficient way to estimate accurate reaction barriers in large alkene molecules. Based on quantum chemistry calculations, high-pressure limit rate constants were calculated via TST. The overall rate constants for the studied alkenes were compared with experimental results from the literature and good agreement was found, indicating that the computed rate constants are reasonable, and thus can be directly used in kinetic modeling studies. Further, it also reveals that the abstraction reaction channels from various reaction sites are dominant in the reaction of long alkenes with OH.

## Figures and Tables

**Table 1:** Computed reaction barriers and enthalpies for the prototype reactions in kcal/mol. ^a^

RC	Prototype Reaction	CCSD(T)-MP2/CBS	
		ΔE	$\Delta_{f}H_{0K}^{o}$
RC1-*cis*	CH_2_=CHCH_2_CH_3_ → •HC=CHCH_2_CH_3_	4.53 (4.35)	−7.58 (−7.69)
		6.01 (6.32) ^b^	
RC1-*trans*	CH_2_=CHCH_2_CH_3_ → •HC=CHCH_2_CH_3_	4.98 (4.78)	−7.10 (−7.23)
		6.04 ^b^	
RC2	CH_2_=CHCH_2_CH_3_ → CH_2_=C•CH_2_CH_3_	2.96 (2.78)	−10.99 (−11.13)
		3.90 (3.79) ^b^	
RC3	CH_2_=CHCH_2_CH_3_ → CH_2_=CHCH•CH_3_	0.27 (0.09)	−34.08 (−34.17)
		−0.01 (−0.41) ^b^	
RC4	CH_2_=CHCH_2_CH_3_ → CH_2_=CHCH_2_CH_2_•	1.43 (1.27)	−17.57 (−17.52)
		2.27 (2.40) ^b^	
RC5	CH_3_CH=CHCH_3_ → CH_3_CH=CHCH_2_•	1.40	−31.33
RC6	CH_2_=CHCH_2_CH_2_CH_3_ → CH=CHCH_2_CH•CH_3_	−0.71	−20.52
RC7	CH_2_=CHCH_2_CH_2_CH_3_ → CH=CHCH_2_CH_2_CH_2_•	1.98	−17.98
RC8	H_2_C=CHCH(CH_3_)_2_ → H_2_C=CHC•(CH_3_)_2_	−0.59	−35.59
RC9	CH_2_=CH(CH_2_)_2_CH_2_CH_3_ → CH_2_=CH(CH_2_)_2_CH•CH_3_	0.68	−20.45
RC10	CH_2_=CHCH_2_CH=CH_2_ → CH_2_=CHCH•CH=CH_2_	0.19	−42.10

^a^ The values in the brackets for reaction class (RC)1 to RC4 represent the results at the CCSD(T)/CBS level and all energies include zero-point vibrational energies (ZPVEs); ^b^ the values outside and inside the parentheses are the results obtained by Sun et al. [20] and Vasu et al. [9].

## Supplementary Materials

Supplementary Materials can be found at https://www.mdpi.com/1422-0067/20/6/1275/s1.
