# Supplementary material for: Reaction Mechanisms and Kinetics of the Hydrogen Abstraction Reactions of C4–C6 Alkenes with Hydroxyl Radical: A Theoretical Exploration
Source: Int J Mol Sci. 2019 Mar 14;20(6):1275. doi: 10.3390/ijms20061275 (PMC6471405; doi:10.3390/ijms20061275)
Supplement: Supplementary file 1 [file ijms-20-01275-s001.pdf]

Reaction mechanisms and Kinetics of the Hydrogen Abstraction Reactions of C_4-C_6 Alkenes with Hydroxyl Radical: A Theoretical Exploration

Quan-De Wang [1,*], Mao-Mao Sun [1] and Jin-Hu Liang [2,*]

1 Low Carbon Energy Institute and School of Chemical Engineering, Jiangsu Province Engineering Lab of High-Efficient Energy Storage Technology and Equipment, China University of Mining and Technology, Xuzhou 221008, People's Republic of China; quandewang@cumt.edu.cn (Q.-D.W.); maomaosun2019@126.com (M.S.)

2 School of Environment and Safety Engineering, North University of China, Taiyuan 030051, People's Republic of China; jhliang@nuc.edu.cn (J.-H.L.)

* Correspondence: quandewang@cumt.edu.cn (Q.-D.W.) or jhliang@nuc.edu.cn (J.-H.L.); Tel.: +86-151-6213-7355 (Q.-D.W.) or +86-152-3411-0325 (J.-H.L.)

Table S1

Full list of the studied abstraction reactions and the computed rate coefficients by employing the corrected reaction barriers (*A*: $cm^3\ mol^{-1}\ s^{-1}$; E_a: $cal\ mol^{-1}$)

	Reaction	Abstraction reaction site	Rate coefficients		
			A	*n*	E_a
1-butene	R1-*cis*	$CH_2=CHCH_2CH_3 \rightarrow \bullet HC=CHCH_2CH_3$	1.04E4	2.70	954
	R1-*trans*	$CH_2=CHCH_2CH_3 \rightarrow \bullet HC=CHCH_2CH_3$	1.10E4	2.65	1558
	R2	$CH_2=CHCH_2CH_3 \rightarrow CH_2=C\bullet CH_2CH_3$	1.60E4	2.58	76
	R3	$CH_2=CHCH_2CH_3 \rightarrow CH_2=CHCH\bullet CH_3$	4.14E4	2.55	-1742
	R4	$CH_2=CHCH_2CH_3 \rightarrow CH_2=CHCH_2CH_2\bullet$	2.51E5	2.28	-1209
2-butene	R5	$CH_3CH=CHCH_3 \rightarrow CH_3CH=CHCH_2\bullet$	5.23E4	2.44	-944
	R6	$CH_3CH=CHCH_3 \rightarrow CH_3C=C\bullet CH_3$	2.10E4	2.55	136
isobutene	R7	$H_2C=C(CH_3)_2 \rightarrow \bullet HC=C(CH_3)_2$	9.01E3	2.69	851
	R8	$H_2C=C(CH_3)_2 \rightarrow H_2C=C(\bullet CH_2)CH_3$	3.33E4	2.50	-1819
1-pentene	R9-*cis*	$CH_2=CHCH_2CH_2CH_3 \rightarrow \bullet CH=CHCH_2CH_2CH_3$	7.46E3	2.69	956
	R9-*trans*	$CH_2=CHCH_2CH_2CH_3 \rightarrow \bullet CH=CHCH_2CH_2CH_3$	1.01E4	2.65	1812
	R10	$CH_2=CHCH_2CH_2CH_3 \rightarrow CH_2=C\bullet CH_2CH_2CH_3$	2.72E4	2.62	-287
	R11	$CH_2=CHCH_2CH_2CH_3 \rightarrow CH_2=CHCH\bullet CH_2CH_3$	2.72E5	2.37	-1929
	R12	$CH_2=CHCH_2CH_2CH_3 \rightarrow CH=CHCH_2CH\bullet CH_3$	5.21E3	2.70	-3523
	R13	$CH_2=CHCH_2CH_2CH_3 \rightarrow CH=CHCH_2CH_2CH_2\bullet$	1.41E4	2.62	-915
2-pentene	R14	$CH_3CH=CHCH_2CH_3 \rightarrow \bullet CH_2CH=CHCH_2CH_3$	5.31E3	2.76	-1305
	R15	$CH_3CH=CHCH_2CH_3 \rightarrow CH_3C\bullet =CHCH_2CH_3$	1.64E4	2.56	191
	R16	$CH_3CH=CHCH_2CH_3 \rightarrow CH_3CH=C\bullet CH_2CH_3$	2.12E4	2.55	-136
	R17	$CH_3CH=CHCH_2CH_3 \rightarrow CH_3CH=CHCH\bullet CH_3$	2.30E4	2.56	-2204
	R18	$CH_3CH=CHCH_2CH_3 \rightarrow CH_3CH=CHCH_2CH_2\bullet$	1.32E4	2.57	-1917
2-methyl-1-butene	R19-*cis*	$H_2C=C(CH_3)CH_2CH_3 \rightarrow \bullet HC=C(CH_3)CH_2CH_3$	1.46E4	2.66	1575
	R19-*trans*	$H_2C=C(CH_3)CH_2CH_3 \rightarrow \bullet HC=C(CH_3)CH_2CH_3$	1.01E4	2.65	1812
	R20	$H_2C=C(CH_3)CH_2CH_3 \rightarrow H_2C=C(CH_2\bullet)CH_2CH_3$	2.12E3	2.79	-1295
	R21	$H_2C=C(CH_3)CH_2CH_3 \rightarrow H_2C=C(CH_3)CH\bullet CH_3$	2.19E4	2.47	-2397
	R22	$H_2C=C(CH_3)CH_2CH_3 \rightarrow H_2C=C(CH_3)CH_2CH_2\bullet$	7.00E3	2.62	-2058
2-methyl-2-butene	R23	$HC(CH_3)=C(CH_3)_2 \rightarrow \bullet C(CH_3)=C(CH_3)_2$	5.08E4	2.53	-772
	R24	$HC(CH_3)=C(CH_3)_2 \rightarrow HC(CH_2\bullet)=C(CH_3)_2$	8.39E3	2.75	-2075
	R25-*cis*	$HC(CH_3)=C(CH_3)_2 \rightarrow HC(CH_3)=C(CH_2\bullet)CH_3$	5.30E3	2.90	-2122
	R25-*trans*	$HC(CH_3)=C(CH_3)_2 \rightarrow HC(CH_3)=C(CH_2\bullet)CH_3$	8.03E3	2.74	-2211
2-methyl-3-butene	R26-*cis*	$H_2C=CHCH(CH_3)_2 \rightarrow \bullet HC=CHCH(CH_3)_2$	1.64E4	2.70	657

	R26-*trans*	$H_2C=CHCH(CH_3)_2 \rightarrow \bullet HC=CHCH(CH_3)_2$	1.54E4	2.67	1169
	R27	$H_2C=CHCH(CH_3)_2 \rightarrow H_2C=C\bullet CH(CH_3)_2$	2.41E4	2.56	-425
	R28	$H_2C=CHCH(CH_3)_2 \rightarrow H_2C=CHC\bullet (CH_3)_2$	7.28E4	2.41	-2655
	R29	$H_2C=CHCH(CH_3)_2 \rightarrow H_2C=CHCH(CH_2\bullet)(CH_3)$	2.41E4	2.55	-1565
1-hexene	R30-*cis*	$CH_2=CHCH_2CH_2CH_2CH_3 \rightarrow \bullet CH=CHCH_2CH_2CH_2CH_3$	8.67E3	2.65	1537
	R30-*trans*	$CH_2=CHCH_2CH_2CH_2CH_3 \rightarrow \bullet CH=CHCH_2CH_2CH_2CH_3$	1.78E4	2.63	1781
	R31	$CH_2=CHCH_2CH_2CH_2CH_3 \rightarrow CH_2=C\bullet CH_2CH_2CH_2CH_3$	1.75E4	2.59	91
	R32	$CH_2=CHCH_2CH_2CH_2CH_3 \rightarrow CH_2=CHCH\bullet CH_2CH_2CH_3$	2.82E5	2.33	-1532
	R33	$CH_2=CHCH_2CH_2CH_2CH_3 \rightarrow CH_2=CHCH_2CH\bullet CH_2CH_3$	1.60E4	2.43	-2847
	R34	$CH_2=CHCH_2CH_2CH_2CH_3 \rightarrow CH_2=CHCH_2CH_2CH\bullet CH_3$	4.69E4	2.59	-989
	R35	$CH_2=CHCH_2CH_2CH_2CH_3 \rightarrow CH_2=CHCH_2CH_2CH_2CH\bullet$	7.44E3	2.57	-377
2-hexene	R36	$CH_3CH=CHCH_2CH_2CH_3 \rightarrow \bullet CH_2CH=CHCH_2CH_2CH_3$	6.25E3	2.76	-1448
	R37	$CH_3CH=CHCH_2CH_2CH_3 \rightarrow CH_3C\bullet =CHCH_2CH_2CH_3$	3.00E4	2.61	-153
	R38	$CH_3CH=CHCH_2CH_2CH_3 \rightarrow CH_3CH=C\bullet CH_2CH_2CH_3$	2.74E4	2.60	-662
	R39	$CH_3CH=CHCH_2CH_2CH_3 \rightarrow CH_3CH=CHCH\bullet CH_2CH_3$	4.65E4	2.58	-2467
	R40	$CH_3CH=CHCH_2CH_2CH_3 \rightarrow CH_3CH=CHCH_2CH\bullet CH_3$	5.41E3	2.72	-3748
	R41	$CH_3CH=CHCH_2CH_2CH_3 \rightarrow CH_3CH=CHCH_2CH_2CH_2\bullet$	1.34E4	2.62	-944
3-hexene	R42	$CH_3CH_2CH=CHCH_2CH_3 \rightarrow \bullet CH_2CH_2CH=CHCH_2CH_3$	6.59E4	2.43	-1730
	R43	$CH_3CH_2CH=CHCH_2CH_3 \rightarrow CH_3CH\bullet CH=CHCH_2CH_3$	2.67E6	2.01	-970
	R44	$CH_3CH_2CH=CHCH_2CH_3 \rightarrow CH_3CH_2C\bullet =CHCH_2CH_3$	1.18E5	2.38	351
1-4-pentadiene	R45-*cis*	$CH_2=CHCH_2CH=CH_2 \rightarrow \bullet CH=CHCH_2CH=CH_2$	1.35E5	2.33	1902
	R45-*trans*	$CH_2=CHCH_2CH=CH_2 \rightarrow \bullet CH=CHCH_2CH=CH_2$	2.37E5	2.33	2170
	R46	$CH_2=CHCH_2CH=CH_2 \rightarrow CH_2=C\bullet CH_2CH=CH_2$	9.20E4	2.27	-232
	R47	$CH_2=CHCH_2CH=CH_2 \rightarrow CH_2=CHCH\bullet CH=CH_2$	1.67E4	2.68	-1585

Table S2

Classification of the studied reactions into 10 RCs and the selected prototype reaction of each RC

RCs	Prototype Reaction	Reactions
**RC1-*cis***	R1	R7, R9, R19, R26, R30, R45
**RC1-*trans***	R1	R7, R9, R19, R26, R30, R45
**RC2**	R2	R6, R10, R15, R16, R23, R27, R31, R37, R38, R44, R46
**RC3**	R3	R11, R17, R21, R32, R39, R43
**RC4**	R4	R18, R22, R29, R42
**RC5**	R5	R8, R14, R20, R24, R25, R36
**RC6**	R12	R33, R40
**RC7**	R13	R35, R41
**RC8**	R28	—
**RC9**	R34	—
**RC10**	R47	—

Table S3

List of the T1 diagnostics during CCSD(T) calculations.

Species	CCSD(T)	
	cc-pvdz	cc-pvtz
OH	0.006	0.007
H2O	0.006	0.007
1-butene	0.009	0.010
2-butene	0.008	0.010

1-pentene	0.008	0.009
2-methyl-3-butene	0.008	0.009
1-hexene	0.008	0.009
1,4-pentadiene	0.010	0.010
RC1-*cis*-TS	0.023	0.024
RC1-*trans*-TS	0.023	0.024
RC2-TS	0.022	0.023
RC3-TS	0.017	0.018
RC4-TS	0.015	0.017
RC5-TS	0.019	0.021
RC6-TS	0.014	0.016
RC7-TS	0.014	0.016
RC8-TS	0.016	0.018
RC9-TS	0.013	0.015
RC10-TS	0.021	0.022

Table S4

Comparisons of the computed reaction barriers for abstraction reactions of ethylene and propene with OH (kcal/mol).

Reaction	Δ*E*	Theoretical level	Reference
CH_2=CH_2 + OH → C_2H_3 + H_2O	5.49	CCSD(T)-MP2/CBS//M06-2X/6-311+g(d,p)	This work
	5.6	QCISD(T)/6-311+G(2df,2p)//QCISD/6-31G(d,p)	Liu *et al.*[a]
	5.4	G2//QCISD/6-31G(d,p)	Liu *et al.*[a]
CH_2=$CHCH_3$ → •HC=$CHCH_3$	4.56 (*cis*)	CCSD(T)-MP2/CBS//M06-2X/6-311+g(d,p)	This work
	5.04 (*trans*)	CCSD(T)-MP2/CBS//M06-2X/6-311+g(d,p)	This work
	4.2 (*cis*)	RQCISD(T)/cc-pV∞Z//B3LYP/6-311++G(d,p)	Zador *et al.*[b]
	4.7 (*trans*)	RQCISD(T)/cc-pV∞Z//B3LYP/6-311++G(d,p)	Zador *et al.*[b]
CH_2=$CHCH_3$ → CH_2=C•CH_3	3.31	CCSD(T)-MP2/CBS//M06-2X/6-311+g(d,p)	This work
	3.3	RQCISD(T)/cc-pV∞Z//B3LYP/6-311++G(d,p)	Zador *et al.*[b]
CH_2=$CHCH_3$ → CH_2=$CHCH_2$•	1.73	CCSD(T)-MP2/CBS//M06-2X/6-311+g(d,p)	This work
	2.9	RQCISD(T)/cc-pV∞Z//B3LYP/6-311++G(d,p)	Zador *et al.*[b]
	0.66	UCCSD(T)/CBS//[5,5]-CASPT2/cc-pVTZ	Szori *et al.*[c]
	1.10	[5,5]-CASPT2/CBS//[5,5]-CASPT2/cc-pVTZ	Szori *et al.*[c]
	1.14	UCCSD(T)/CBS(Extrapolation of cc-pVXZ Basis)	Szori *et al.*[c]
	1.6	CCSD(T)/cc-pVDZ// B3LYP/cc-pVTZ	Huyhn *et al.* [d]
	2.6	PMP2/aug-cc-PVQZ//MP2/cc-PVTZ	Zhou *et al.*[e]

[a]Liu *et al.*, Theoretical study on mechanisms of the high-temperature reactions C_2H_3+H_2O and C_2H_4+OH, Phys. Chem. Chem. Phys.,2002, 4,1021–1027.

[b]Zador *et al.*, The reaction between propene and hydroxyl, Phys. Chem. Chem. Phys., 2009, 11, 11040-11053.

[c]Szori *et al.*, High Accuracy ab Initio Calculations on Reactions of OH with 1-Alkenes. The Case of Propene, J. Chem. Theory Comput., 2009, 5, 2313-2321.

[d]Huyhn *et al.*, Kinetics of Enol Formation from Reaction of OH with Propene, J. Phys. Chem. A, 2009, 113, 3177-3185.

[e]Zhou *et al.*, Kinetics and Mechanism for Formation of Enols in Reaction of Hydroxide Radical with Propene, J. Phys. Chem. A, 2009, 113, 2372-2382.

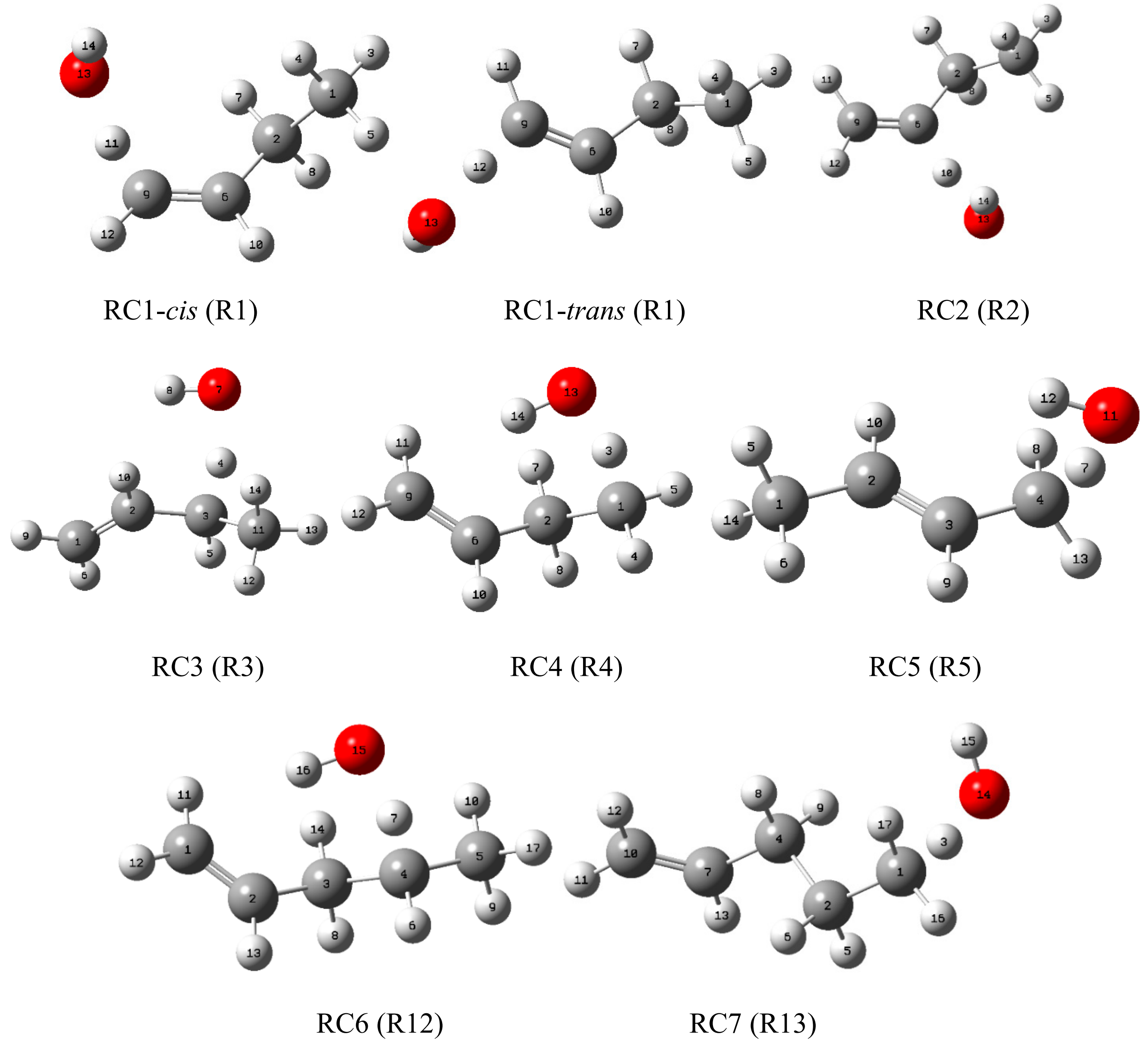

Figure S1 Optimized geometry of TS structure of prototype reactions

Additional remarks to Figure S1: Conservation of the Reaction Centers Reactions in the same reaction class should have the same reactive moiety and share similarities in the shape of the potential energy surfaces along the reaction path. Thus, the reaction centers from the optimized transition state structures for the reactions in the same class should be identical. Figure S1 displays the optimized TS structure of prototype reactions, and the labeling of atoms involved in the reaction centers are given. Table S3 to S10 lists key parameters including bond lengths and bond angles at the reaction centers in the optimized TS structures. The computed rate coefficients and energy information are also provided in these tables. It can be seen that the differences of the bond lengths and bond angles of the reaction centers at TSs are nearly identical. Thus, the reactive moiety and potential energy surfaces

along the reaction path are very well conserved among the reactions.

Table S5

Optimized geometric parameters of reaction centers at transition states, computed barriers and rate coefficients for RC1-*cis*.

RC1-*cis*	$d_{C9\text{-}H11}$	$d_{H11\text{-}O13}$	$A_{C9\text{-}H11\text{-}O13}$	M06-2X barrier (kcal mol^{-1})	Corrected Barrier (kcal mol^{-1})	**Rate coefficients**		
						A	*n*	E_a
R1-*cis*	1.21	1.31	162	3.59	4.34	1.04E4	2.70	954
R7	1.21	1.31	162	3.45	4.19	9.01E3	2.69	851
R9-*cis*	1.20	1.31	163	3.58	4.33	7.46E3	2.69	956
R19-*cis*	1.21	1.31	162	3.92	4.71	1.46E4	2.66	1575
R26-*cis*	1.20	1.31	162	3.35	4.08	1.64E4	2.70	657
R30-*cis*	1.20	1.31	163	3.83	4.61	8.67E3	2.65	1537
R45-*cis*	1.21	1.31	163	3.83	4.61	1.35E5	2.33	1902
Averaged value	1.21	1.31	162	3.65	4.41			
SE of Mean				0.08	0.09			
Standard Deviation				0.21	0.23			

d and A represent the bond length and the bond angle with units of Å and degree, respectively.

Table S6

Optimized geometric parameters of reaction centers at transition states, computed barriers and rate coefficients for RC1-*trans*.

RC1-*trans*	$d_{C9\text{-}H12}$	$d_{H12\text{-}O13}$	$A_{C9\text{-}H12\text{-}O13}$	M06-2X barrier (kcal mol^{-1})	Corrected Barrier (kcal mol^{-1})	**Rate coefficients**		
						A	*n*	E_a
R1-*trans*	1.20	1.30	1.62	4.16	4.97	1.10E4	2.65	1558
R7	1.21	1.31	162	3.45	4.19	9.01E3	2.69	851
R9-*trans*	1.20	1.30	1.62	4.28	5.10	1.01E4	2.65	1812
R19-*trans*	1.20	1.31	1.62	3.47	4.21	1.10E4	2.66	1080
R26-*trans*	1.20	1.31	1.62	3.90	4.68	1.54E4	2.67	1169
R30-*trans*	1.20	1.30	1.62	4.23	5.04	1.78E4	2.63	1781
R45-*trans*	1.21	1.30	1.62	4.30	5.12	2.37E5	2.33	2170
Averaged value	1.20	1.30	1.62	3.97	4.76			
SE of Mean				0.14	0.15			
Standard Deviation				0.37	0.41			

d and A represent the bond length and the bond angle with units of Å and degree, respectively.

Table S7

Optimized geometric parameters of reaction centers at transition states, computed barriers and rate coefficients for RC2.

RC2	$d_{C6\text{-}H10}$	$d_{H10\text{-}O13}$	$A_{C6\text{-}H10\text{-}O13}$	M06-2X barrier (kcal mol^{-1})	Corrected Barrier (kcal mol^{-1})	**Rate coefficients**		
						A	*n*	E_a
R2	1.18	1.37	161	2.24	2.87	1.60E4	2.58	76
R6	1.18	1.37	161	2.22	2.85	2.10E4	2.55	136
R10	1.18	1.36	160	1.99	2.59	2.72E4	2.62	-287

R15	1.18	1.37	161	2.19	2.81	1.64E4	2.56	191
R16	1.18	1.38	161	1.82	2.41	2.12E4	2.55	-136
R23	1.18	1.38	162	1.39	1.94	5.08E4	2.53	-772
R27	1.18	1.37	161	1.74	2.32	2.41E4	2.56	-425
R31	1.18	1.37	161	2.17	2.79	1.75E4	2.59	91
R37	1.18	1.37	160	1.97	2.57	3.00E4	2.61	-153
R38	1.18	1.37	160	1.46	2.01	2.74E4	2.60	-662
R44	1.18	1.38	161	1.83	2.42	1.18E5	2.38	351
R46	1.19	1.33	162	1.74	2.32	9.20E4	2.27	-232
Averaged value	1.18	1.37	161	1.90	2.49			
SE of Mean				0.08	0.09			
Standard Deviation				0.29	0.31			

d and A represent the bond length and the bond angle with units of Å and degree, respectively.

Table S8

Optimized geometric parameters of reaction centers at transition states, computed barriers and rate coefficients for RC3.

RC3	$d_{C3\text{-}H14}$	$d_{H4\text{-}O7}$	$A_{C3\text{-}H4\text{-}O7}$	M06-2X barrier (kcal mol^{-1})	Corrected Barrier (kcal mol^{-1})	**Rate coefficients**		
						A	*n*	E_a
R3	1.15	1.51	164	0.03	0.45	4.14E4	2.55	-1742
R11	1.15	1.53	166	-0.39	-0.01	2.72E5	2.37	-1929
R17	1.15	1.52	163	-0.35	0.03	2.30E4	2.56	-2204
R21	1.16	1.50	157	-0.47	-0.10	2.19E4	2.47	-2397
R32	1.15	1.53	166	-0.21	0.19	2.82E5	2.33	-1532
R39	1.14	1.55	165	-0.73	-0.38	4.65E4	2.58	-2467
R43	1.15	1.52	163	-0.26	0.13	2.67E6	2.01	-970
Averaged value	1.15	1.52	163	-0.34	0.04			
SE of Mean				0.09	0.10			
Standard Deviation				0.24	0.25			

d and A represent the bond length and the bond angle with units of Å and degree, respectively.

Table S9

Optimized geometric parameters of reaction centers at transition states, computed barriers and rate coefficients for RC4.

RC4	$d_{C1\text{-}H3}$	$d_{H3\text{-}O13}$	$d_{C6\text{-}H14}$	$A_{C1\text{-}H3\text{-}O13}$	M06-2X barrier (kcal mol^{-1})	Corrected Barrier (kcal mol^{-1})	**Rate coefficients**		
							A	*n*	E_a
R4	1.18	1.38	2.64	163	0.72	1.20	2.51E5	2.28	-1209
R18	1.18	1.38	2.60	162	0.55	1.02	1.32E4	2.57	-1917
R22	1.19	1.38	2.55	163	0.55	1.02	7.00E3	2.62	-2058
R29	1.18	1.37	2.63	163	0.60	1.07	2.41E4	2.55	-1565
R42	1.18	1.38	2.61	162	0.52	0.99	6.59E4	2.43	-1730
Averaged value	1.18	1.38	2.61	163	0.59	1.06			
SE of Mean					0.04	0.04			
Standard Deviation					0.08	0.08			

d and A represent the bond length and the bond angle with units of Å and degree, respectively.

Table S10

Optimized geometric parameters of reaction centers at transition states, computed barriers and rate coefficients for RC5.

RC5	$d_{C4\text{-}H7}$	$d_{H7\text{-}O11}$	$A_{C4\text{-}H7\text{-}O11}$	M06-2X barrier (kcal mol^{-1})	Corrected Barrier (kcal mol^{-1})	**Rate coefficients**		
						A	n	E_a
R5	1.16	1.48	161	0.95	1.46	5.23E4	2.44	-944
R8	1.16	1.46	161	0.52	0.99	3.33E4	2.50	-1819
R14	1.16	1.48	161	0.87	1.37	5.31E3	2.76	-1305
R20	1.16	1.46	161	0.82	1.31	2.12E3	2.79	-1295
R24	1.16	1.48	160	0.09	0.52	8.39E3	2.75	-2075
R25-*cis*	1.16	1.48	160	0.03	0.45	5.30E3	2.90	-2122
R25-*trans*	1.16	1.48	160	0.08	0.50	8.03E3	2.74	-2211
R36	1.16	1.48	161	0.77	1.26	6.25E3	2.76	-1448
Averaged value	1.16	1.48	161	0.52	0.98			
SE of Mean				0.14	0.15			
Standard Deviation				0.39	0.43			

d and A represent the bond length and the bond angle with units of Å and degree, respectively.

Table S11

Optimized geometric parameters of reaction centers at transition states, computed barriers and rate coefficients for RC6.

RC6	$d_{C4\text{-}H7}$	$d_{H7\text{-}O15}$	$d_{C2\text{-}H16}$	$A_{C4\text{-}H7\text{-}O15}$	M06-2X barrier (kcal mol^{-1})	Corrected Barrier (kcal mol^{-1})	**Rate coefficients**		
							A	n	E_a
R12	1.16	1.44	2.65	164	-1.22	-0.92	5.21E3	2.70	-3523
R33	1.16	1.45	2.68	164	-1.11	-0.80	1.60E4	2.43	-2847
R40	1.16	1.44	2.62	163	-1.44	-1.16	5.41E3	2.72	-3748
Averaged value	1.16	1.44	2.65	164	-1.26	-0.96			
SE of Mean					0.10	0.11			
Standard Deviation					0.17	0.18			

d and A represent the bond length and the bond angle with units of Å and degree, respectively.

Table S12

Optimized geometric parameters of reaction centers at transition states, computed barriers and rate coefficients for RC7.

RC7	$d_{C1\text{-}H3}$	$d_{H3\text{-}O14}$	$A_{C1\text{-}H3\text{-}O14}$	M06-2X barrier (kcal mol^{-1})	Corrected Barrier (kcal mol^{-1})	**Rate coefficients**		
						A	n	E_a
R13	1.17	1.42	169	1.68	2.25	1.41E4	2.62	-915
R35	1.17	1.42	169	1.99	2.59	7.44E3	2.57	-377
R41	1.17	1.42	169	1.66	2.23	1.34E4	2.62	-944
Averaged value	1.17	1.42	169	1.78	2.36			
SE of Mean				0.11	0.12			
Standard Deviation				0.19	0.20			

d and A represent the bond length and the bond angle with units of Å and degree, respectively.

Table S13

Theoretical predicted potential energies in kcal mol^{-1} at CCSD(T)-MP2/CBS level.

Reaction Class	Reaction	RC	TS (ΔE)	PC	Products ($\Delta_f H^o_{0K}$)
**RC1-cis**	CH_2=$CHCH_2CH_3$ → •HC=$CHCH_2CH_3$	-1.05	4.53	-9.01	-7.58
**RC1-trans**	CH_2=$CHCH_2CH_3$ → •HC=$CHCH_2CH_3$	-1.32	4.98	-7.74	-7.10
**RC2**	CH_2=$CHCH_2CH_3$ → CH_2=C•CH_2CH_3	-1.12	2.96	-11.51	-10.99
**RC3**	CH_2=$CHCH_2CH_3$ → CH_2=CHCH•CH_3	-2.20	0.27	-36.02	-34.08
**RC4**	CH_2=$CHCH_2CH_3$ → CH_2=$CHCH_2CH_2$•	-1.78	1.43	-19.27	-17.57
**RC5**	CH_3CH=$CHCH_3$ → CH_3CH=$CHCH_2$•	-1.37	1.40	-32.71	-31.33
**RC6**	CH_2=$CHCH_2CH_2CH_3$ → CH=$CHCH_2CH$•CH_3	-2.06	-0.71	-21.96	-20.52
**RC7**	CH_2=$CHCH_2CH_2CH_3$ → CH=$CHCH_2CH_2CH_2$•	-0.26	1.98	-19.42	-17.98
**RC8**	H_2C=CHCH$(CH_3)_2$ → H_2C=CHC•$(CH_3)_2$	-1.91	-0.59	-37.81	-35.59
**RC9**	CH_2=$CHCH_2CH_2CH_2CH_3$ → CH_2=$CHCH_2CH_2CH$•CH_3	0.05	0.68	-21.92	-20.45
**RC10**	CH_2=$CHCH_2CH$=CH_2 → CH_2=CHCH•CH=CH_2	-1.47	0.19	-43.45	-42.10

Table S14

Fitted Arrhenius rate coefficients (*A*: cm^3 mol^{-1} s^{-1}, E_a: cal mol^{-1}) for the prototype reactions at CCSD(T)-MP2/CBS//M06-2X/6-311+G(d,p) level.

Reaction Class	Reaction	*A*	*n*	E_a
**RC1-*cis***	CH_2=$CHCH_2CH_3$ → •HC=$CHCH_2CH_3$	1.04×10^4	2.70	1071
**RC1-*trans***	CH_2=$CHCH_2CH_3$ → •HC=$CHCH_2CH_3$	1.10×10^4	2.65	1508
**RC2**	CH_2=$CHCH_2CH_3$ → CH_2=C•CH_2CH_3	1.61×10^4	2.58	105
**RC3**	CH_2=$CHCH_2CH_3$ → CH_2=CHCH•CH_3	4.14×10^4	2.55	-1965
**RC4**	CH_2=$CHCH_2CH_3$ → CH_2=$CHCH_2CH_2$•	7.65×10^4	2.41	-1171
**RC5**	CH_3CH=$CHCH_3$ → CH_3CH=$CHCH_2$•	1.39×10^5	2.34	-746
**RC6**	CH_2=$CHCH_2CH_2CH_3$ → CH=$CHCH_2CH$•CH_3	5.21×10^3	2.70	-3368
**RC7**	CH_2=$CHCH_2CH_2CH_3$ → CH=$CHCH_2CH_2CH_2$•	1.41×10^4	2.62	-1250
**RC8**	H_2C=CHCH$(CH_3)_2$ → H_2C=CHC•$(CH_3)_2$	7.28×10^4	2.41	-2610
**RC9**	CH_2=$CHCH_2CH_2CH_2CH_3$ → CH_2=$CHCH_2CH_2CH$•CH_3	8.94×10^4	2.45	-1582
**RC10**	CH_2=$CHCH_2CH$=CH_2 → CH_2=CHCH•CH=CH_2	1.67×10^4	2.68	-1836

Table S15

Comparisons of computed overall rate constants with experimental results (cm^3 mol^{-1} s^{-1}).

T(K)	1-butene, this work	T(K)	1-butene[a]	T(K)	1-butene[b]	T(K)	1-butene[c]
**500**	3.54E+12	946	1.04E+13	650	3.97E+12	880	1.02E+13
**600**	4.33E+12	954	1.06E+13	691	4.55E+12	894	1.05E+13
**700**	5.37E+12	980	1.09E+13	732	4.95E+12	951	1.13E+13
**800**	6.65E+12	1008	1.14E+13	778	5.37E+12	1000	1.24E+13
**900**	8.18E+12	1057	1.22E+13	833	5.82E+12	980	1.21E+13

**1000**	9.96E+12	1162	1.6E+13			1057	1.32E+13
**1100**	1.2E+13	1222	1.53E+13			1095	1.4E+13
**1200**	1.43E+13	1256	1.72E+13			1097	1.4E+13
**1300**	1.7E+13					1223	1.8E+13
**1400**	1.99E+13					1250	1.88E+13
**1500**	2.31E+13					1329	2.3E+13
**1600**	2.67E+13					1337	2.41E+13
**1700**	3.06E+13					1341	2.36E+13
**1800**	3.49E+13						
**1900**	3.95E+13						
**2000**	4.45E+13						
**2100**	4.99E+13						
**2200**	5.57E+13						
**2300**	6.19E+13						
**2400**	6.86E+13						
**2500**	7.56E+13						

[a] F. Khaled, J. Badra, A. Farooq, A shock tube study of C4-C6 straight chain alkenes + OH reactions, Proc. Combust. Inst. 36 (2017) 289-298.

[b] F.P. Tully, Hydrogen-atom abstraction from alkenes by OH, ethene and 1-butene, Chem. Phys. Lett. 143 (1988) 510-514.

[c] S.S. Vasu, L.K. Huynh, D.F. Davidson, R.K. Hanson, D.M. Golden, Reactions of OH with butene isomers: measurements of the overall rates and a theoretical study, J. Phys. Chem. A 115 (2011) 2549-2556.

Table S16

Comparisons of computed overall rate constants with experimental results ($cm^3\ mol^{-1}\ s^{-1}$).

T(K)	1-hexene, this work	2-hexene, this work	1-pentene, this work	T(K)	1-hexene[a]	T(K)	2-hexene[a]	T(K)	1-pentene[a]
**500**	5.69E+12	1.24E+13	5.34E+12	836	1.11E+13	881	1.72E+13	875	1.1E+13
**600**	6.7E+12	1.29E+13	5.7E+12	920	1.3E+13	958	1.95E+13	910	1.2E+13
**700**	8.06E+12	1.44E+13	6.57E+12	945	1.34E+13	1068	2.3E+13	944	1.25E+13
**800**	9.75E+12	1.66E+13	7.81E+12	1000	1.4E+13	1133	2.5E+13	1004	1.35E+13

**900**	1.17E+13	1.93E+13	9.38E+12	1058	1.56E+13	1183	2.7E+13	1107	1.6E+13
**1000**	1.41E+13	2.26E+13	1.13E+13	1087	1.68E+13	1200	2.75E+13	1160	1.7E+13
**1100**	1.67E+13	2.64E+13	1.35E+13	1117	1.83E+13	1214	2.82E+13	1128	1.7E+13
**1200**	1.97E+13	3.08E+13	1.61E+13	1270	2.5E+13	1293	3.1E+13	1140	1.75E+13
**1300**	2.31E+13	3.57E+13	1.9E+13	1387	3.35E+13	1304	3.15E+13	1180	1.92E+13
**1400**	2.68E+13	4.11E+13	2.23E+13			1377	3.5E+13	1247	2.04E+13
**1500**	3.09E+13	4.71E+13	2.59E+13					1337	2.6E+13
**1600**	3.53E+13	5.36E+13	3E+13					1379	2.9E+13
**1700**	4.02E+13	6.08E+13	3.44E+13						
**1800**	4.55E+13	6.86E+13	3.93E+13						
**1900**	5.12E+13	7.7E+13	4.46E+13						
**2000**	5.74E+13	8.61E+13	5.04E+13						
**2100**	6.4E+13	9.58E+13	5.67E+13						
**2200**	7.11E+13	1.06E+14	6.34E+13						
**2300**	7.86E+13	1.17E+14	7.06E+13						
**2400**	8.67E+13	1.29E+14	7.84E+13						
**2500**	9.52E+13	1.42E+14	8.66E+13						

[a] Experimental results from F. Khaled, J. Badra, A. Farooq, A shock tube study of C4-C6 straight chain alkenes + OH reactions, Proc. Combust. Inst. 36 (2017) 289-298.

Table S17

Calculated single-point energies and zero-point energies (ZPE) in hartree for the studied reaction systems.

Species	E(UM062X/6-311+G(d,p))	ZPE
OH	-75.72657462	0.008577
H2O	-76.42088886	0.021623
React1	-157.1747494	0.108773
React2	-157.1789881	0.108241
React3	-157.180765	0.108608
React4	-196.4783619	0.137668
React5	-196.4820913	0.137081
React6	-196.4839572	0.137149

React7	-196.4853743	0.136898
React8	-196.4802844	0.13713
React9	-235.7817448	0.165844
React10	-235.7856962	0.165913
React11	-235.7851736	0.165821
React12	-195.248625	0.113824
R1-cis-prod	-156.4908635	0.094925
R1-trans-prod	-156.4904558	0.095245
R2-prod	-156.4964971	0.094799
R3-prod	-156.5312636	0.094626
R4-prod	-156.5043393	0.093932
R5-prod	-156.5312636	0.094624
R6-prod	-156.5001361	0.094702
R7-prod	-156.4956739	0.094494
R8-prod	-156.5292475	0.094167
R9-cis-prod	-195.7944445	0.123301
R9-trans-prod	-195.7940756	0.123993
R10-prod	-195.8001845	0.12384
R11-prod	-195.8343491	0.123348
R12-prod	-195.8127452	0.122319
R13-prod	-195.8081817	0.122382
R14-prod	-195.8343491	0.123348
R15-prod	-195.8034566	0.123773
R16-prod	-195.8030861	0.12335
R17-prod	-195.8387806	0.123414
R18-prod	-195.8118086	0.121976
R19-cis-prod	-195.8000031	0.123642
R19-trans-prod	-195.799347	0.122925
R20-prod	-195.8317518	0.123598
R21-prod	-195.8367075	0.122713
R22-prod	-195.8134742	0.122255
R23-prod	-195.8072641	0.123455
R24-prod	-195.8397595	0.123223
R25-cis-prod	-195.8367075	0.122713
R25-trans-prod	-195.8367586	0.12297
R26-cis-prod	-195.7965741	0.123255
R26-trans-prod	-195.796184	0.123375
R27-prod	-195.8018374	0.123224
R28-prod	-195.8397595	0.123223
R29-prod	-195.8097519	0.122054
R30-cis-prod	-235.0979469	0.15239
R30-trans-prod	-235.0974611	0.152178
R31-prod	-235.1036272	0.152273

R32-prod	-235.1377692	0.152835
R33-prod	-235.1159294	0.151317
R34-prod	-235.116533	0.150975
R35-prod	-235.1117189	0.150802
R36-prod	-235.1380037	0.152228
R37-prod	-235.1070252	0.151935
R38-prod	-235.1067638	0.152128
R39-prod	-235.1418761	0.151782
R40-prod	-235.1201731	0.150802
R41-prod	-235.1155668	0.150814
R42-prod	-235.1148737	0.150787
R43-prod	-235.1418761	0.151783
R44-prod	-235.1063925	0.15256
R45-cis-prod	-194.5646391	0.100053
R45-trans-prod	-194.564219	0.100375
R46-prod	-194.5690103	0.100053
R47-prod	-194.6195102	0.100314
R1-cis-TS	-232.8928435	0.114506
R1-trans-TS	-232.892011	0.114588
R2-TS	-232.8956683	0.115203
R3-TS	-232.900016	0.116044
R4-TS	-232.8985995	0.115732
R5-TS	-232.9028875	0.115624
R6-TS	-232.9000027	0.114738
R7-TS	-232.8989887	0.114243
R8-TS	-232.9046999	0.115316
R9-cis-TS	-272.1965187	0.143452
R9-trans-TS	-272.19562	0.143678
R10-TS	-272.1993037	0.1437
R11-TS	-272.2041943	0.144832
R12-TS	-272.2050689	0.144377
R13-TS	-272.1999908	0.143912
R14-TS	-272.2059772	0.144322
R15-TS	-272.2031665	0.143593
R16-TS	-272.2038356	0.143673
R17-TS	-272.207943	0.144333
R18-TS	-272.2063438	0.144166
R19-cis-TS	-272.2017279	0.143087
R19-trans-TS	-272.2023742	0.143025
R20-TS	-272.2080453	0.144511
R21-TS	-272.2102109	0.144616
R22-TS	-272.2081633	0.144191
R23-TS	-272.2072585	0.142917

R24-TS	-272.2102344	0.143852
R25-cis-TS	-272.2101275	0.143653
R25-trans-TS	-272.2102344	0.143835
R26-cis-TS	-272.1984973	0.142587
R26-trans-TS	-272.1976117	0.142573
R27-TS	-272.2017963	0.143354
R28-TS	-272.2072913	0.144494
R29-TS	-272.2040864	0.143842
R30-cis-TS	-311.4999488	0.172084
R30-trans-TS	-311.4990494	0.171807
R31-TS	-311.5027856	0.172275
R32-TS	-311.507597	0.173333
R33-TS	-311.5090093	0.173304
R34-TS	-311.506101	0.172994
R35-TS	-311.5035813	0.172806
R36-TS	-311.509613	0.173009
R37-TS	-311.5068329	0.172126
R38-TS	-311.5076696	0.172141
R39-TS	-311.5121256	0.173133
R40-TS	-311.5127183	0.172593
R41-TS	-311.5063414	0.172303
R42-TS	-311.5059916	0.172462
R43-TS	-311.5110196	0.173217
R44-TS	-311.5069453	0.172455
R45-cis-TS	-270.9665658	0.119806
R45-trans-TS	-270.9654879	0.119461
R46-TS	-270.9701806	0.120092
R47-TS	-270.9741293	0.121192
C2H4	-78.56360706	0.051316
TS-C2H4	-154.2803275	0.057202
C3H6	-117.8717307	0.079989
TS-C3H6-RC1-cis	-193.5897354	0.085659
TS-C3H6-RC1-trans	-193.5887223	0.085589
TS-C3H6-RC2	-193.5917086	0.086159
TS-C3H6-RC5	-193.5948234	0.087137

Table S18

Calculated single-point energies in hartree for selected reaction systems.

Species	CCSD(T)/cc-pvdz	CCSD(T)/cc-pvtz	MP2/cc-pvdz	MP2/cc-pvtz	MP2/cc-pvqz	CCSD(T)/cc-pvqz
OH	-75.559305643	-75.637722322	-75.542840711555	-75.618886250836	-75.643530916793	-75.661627517
H2O	-76.240987437	-76.332156682	-76.228376530062	-76.318593069633	-76.347601616945	-76.359766067

C2H4	-78.354406774	-78.438663344	-78.314541209632	-78.399221021288	-78.425201638543	
TS-C2H4	-153.89880519	-154.06409455	-153.83596285509	-153.99845799984	-154.04940409695	
C3H6	-117.55644854	-117.6823487	-117.49947835275	-117.62578290783	-117.66482084849	
TS-C3H6-RC1-cis	-193.10334628	-193.30953293	-193.02363710599	-193.22711543254	-193.29086647547	
TS-C3H6-RC1-trans	-193.10131127	-193.30825211	-193.02179798771	-193.2259612903	-193.28995796668	
TS-C3H6-RC2	-193.10501752	-193.31168414	-193.02633900651	-193.23018147423	-193.29408411561	
TS-C3H6	-193.10828489	-193.31512207	-193.03043029753	-193.23445693297	-193.29838363529	
React1	-156.754405	-156.92169085	-156.6799613855	-156.84767046814	-156.89964732668	-156.9679079
React2	-156.75817436	-156.92546812	-156.68401674594	-156.85173711848	-156.90377090088	
React3	-156.75959016	-156.92722006	-156.68571536365	-156.85384283473	-156.90598139371	
React4	-195.95286387	-196.16158008	-195.86076498682	-196.0699017581	-196.13489122578	
React6	-195.95719403	-196.16643644	-195.86580864165	-196.07556982444	-196.14069163016	
React8	-195.95492241	-196.16352003	-195.86342891908	-196.0725599101	-196.13752487872	
React9	-235.15121027	-235.40133034	-235.04139799826	-235.29194935638	-235.36994261597	
React12	-194.72840448	-194.92927915	-194.64257500356	-194.84356849027	-194.9069371323	
R1-cis-prod	-156.07354212	-156.2345848	-155.99231539331	-156.15270862336	-156.20276029325	-156.27954803
R1-trans-prod	-156.07326093	-156.23419362	-155.99190072867	-156.1521901796	-156.20221199413	-156.27913407
R2-prod	-156.0784711	-156.23975211	-155.99766602456	-156.15840096359	-156.20856713414	-156.28482868
R3-prod	-156.11391431	-156.27586664	-156.03287521222	-156.19403474453	-156.24443607906	-156.32119853
R4-prod	-156.08714132	-156.24902354	-156.01350939216	-156.1751634022	-156.22564074991	-156.29413738
R5-prod	-156.11392008	-156.27586769	-156.03287915037	-156.19403405471	-156.24443477381	
R8-prod	-156.1122394	-156.27461279	-156.03144213312	-156.19311862229	-156.24354051056	
R12-prod	-195.28967798	-195.49297589	-195.19754941825	-195.40064979324	-195.4642303335	
R13-prod	-195.28567994	-195.48911969	-195.19473493959	-195.39788452264	-195.46136432928	
R21-prod	-195.31301195	-195.51712331	-195.21439151954	-195.41783984933	-195.48136358389	
R28-prod	-195.31590788	-195.5199986	-195.21761637252	-195.42100050573	-195.48458204341	
R34-prod	-234.48819798	-234.73306631	-234.37910160859	-234.62368149741	-234.70024622377	
R47-prod	-194.10156324	-194.2970814	-194.00121683501	-194.19521919507	-194.25676370491	
R1-cis-TS	-232.30150839	-232.54907238	-232.20438987819	-232.44926240044	-232.52589551838	-232.61957967
R1-trans-TS	-232.29954329	-232.54792615	-232.20261187774	-232.44822196253	-232.52512963427	-232.61875138
R2-TS	-232.30482422	-232.55237629	-232.20855282129	-232.45333094693	-232.52986539775	-232.62280589
R3-TS	-232.30894392	-232.55701712	-232.21448019258	-232.45981940625	-232.53663563845	-232.62772329
R4-TS	-232.3059674	-232.55474626	-232.21331210511	-232.4592028878	-232.53605099674	-232.62550215
R5-TS	-232.31077036	-232.55898006	-232.21617808914	-232.46148227503	-232.53838365843	
R8-TS	-232.31325895	-232.56120463	-232.21900807621	-232.46426317798	-232.54099587441	
R12-TS	-271.50797628	-271.79793493	-271.39758147532	-271.68462490094	-271.77440470698	
R13-TS	-271.50455683	-271.79359192	-271.39382665185	-271.68005697055	-271.76963063341	
R21-TS	-271.51356951	-271.80285377	-271.40269456789	-271.6892983027	-271.77891147956	
R28-TS	-271.51089481	-271.80037969	-271.40020353281	-271.6870237498	-271.77678165169	
R34-TS	-310.70611669	-311.03639975	-310.57768009138	-310.90516973259	-311.00768790088	
R47-TS	-270.28313837	-270.56465065	-270.17428813775	-270.45291662222	-270.54120357874	

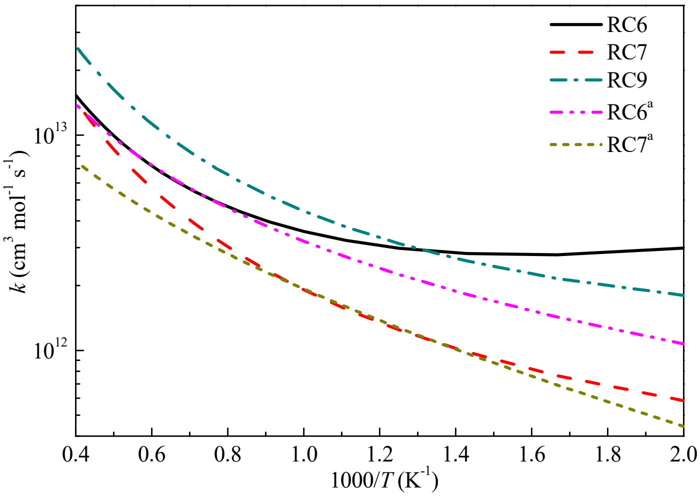

Figure S2 Comparisons of computed rate constants for prototype reactions of RC6, RC7 and RC9 with literature data ([a]Zhang *et al.,* Combust. Flame, 2016, 172, 116-135).

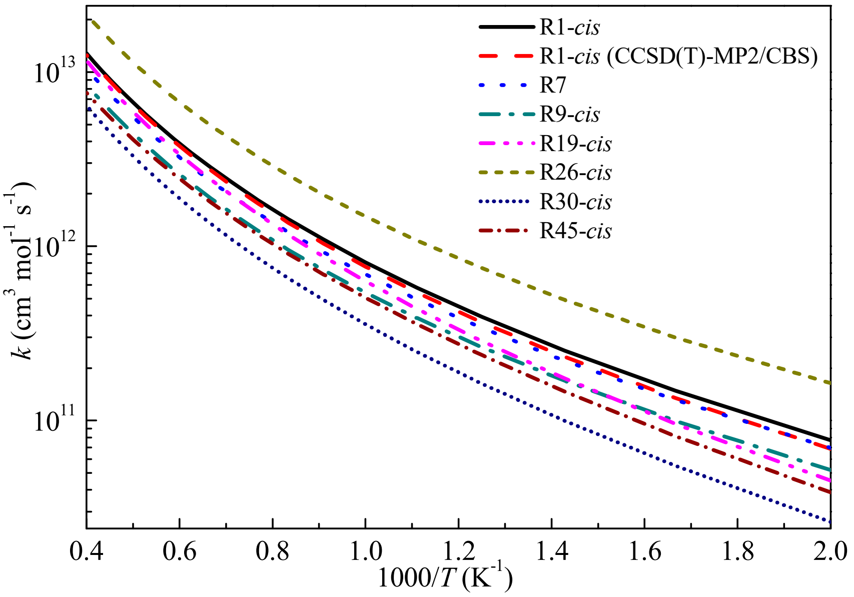

Figure S3 Predicted rate constants for RC1-*cis* in this work.

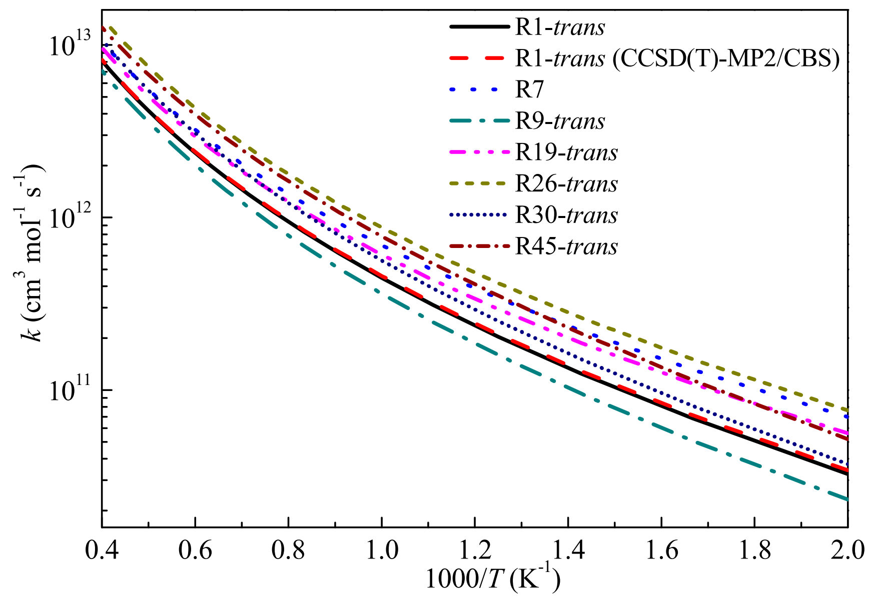

Figure S4 Predicted rate constants for RC1-*trans* in this work.

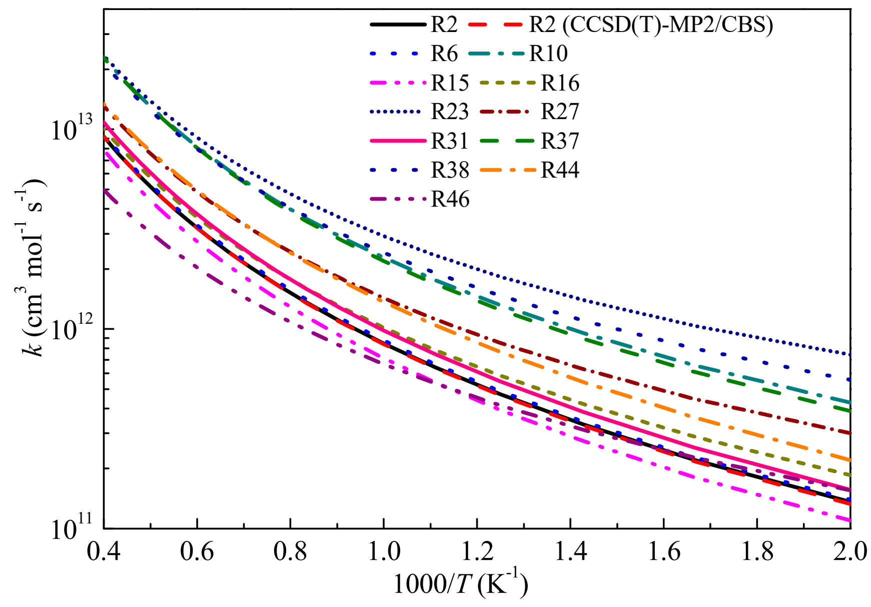

Figure S5 Predicted rate constants for RC2 in this work.

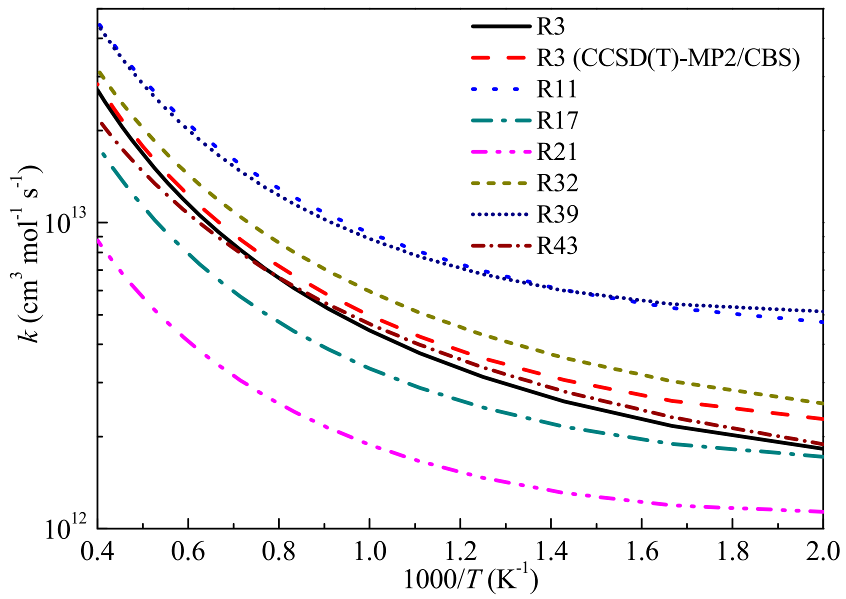

Figure S6 Predicted rate constants for RC3 in this work.

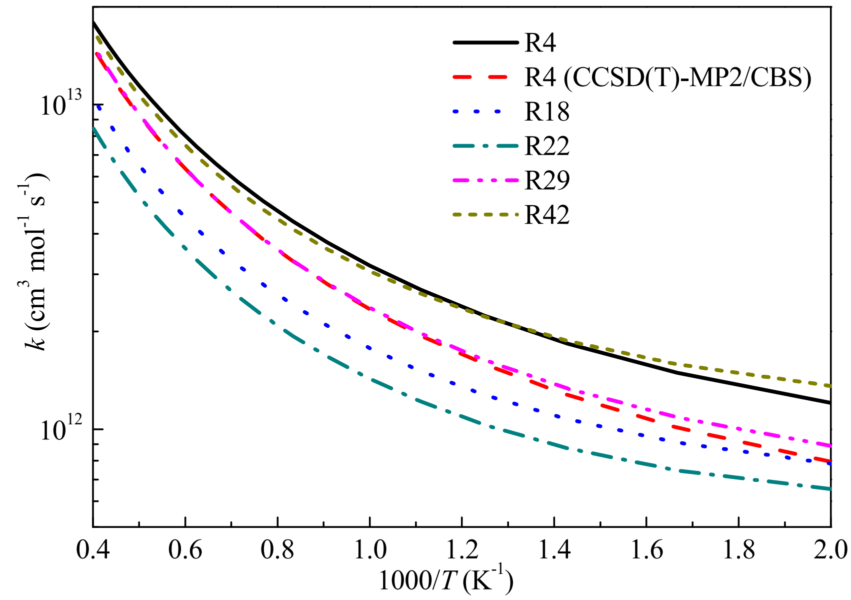

Figure S7 Predicted rate constants for RC4 in this work.

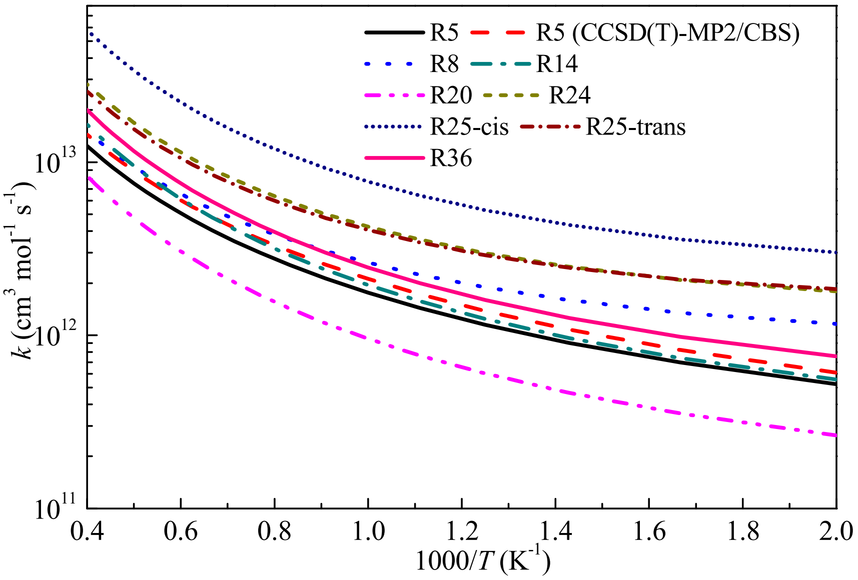

Figure S8 Predicted rate constants for RC5 in this work.

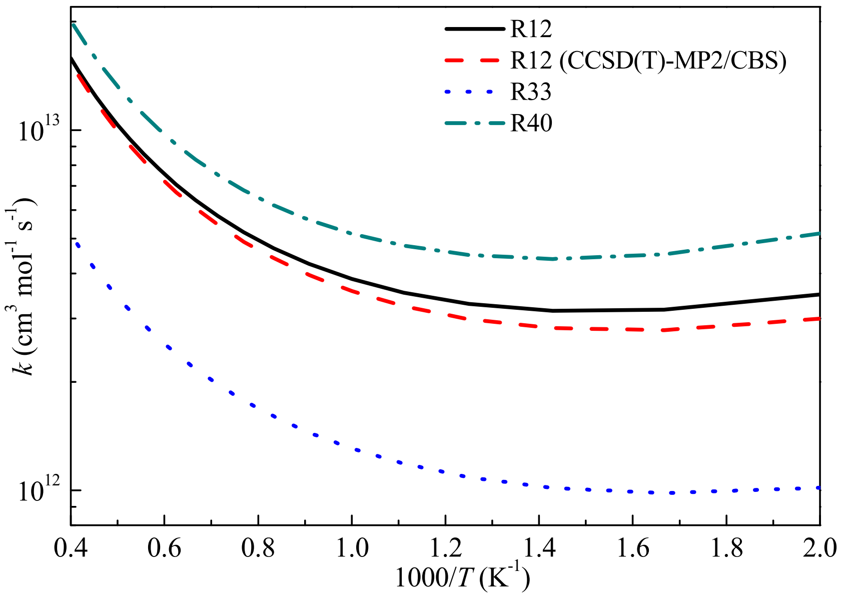

Figure S9 Predicted rate constants for RC6 in this work.

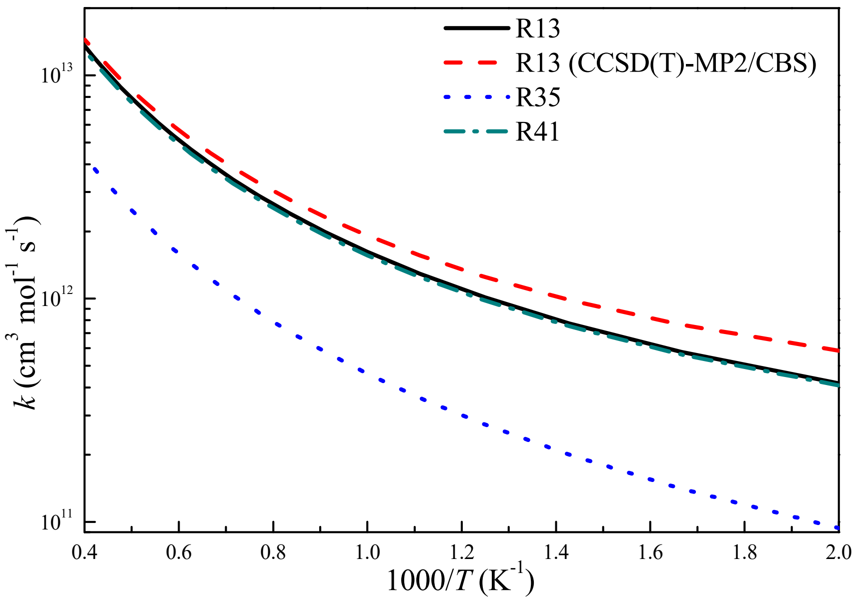

Figure S10 Predicted rate constants for RC7 in this work.

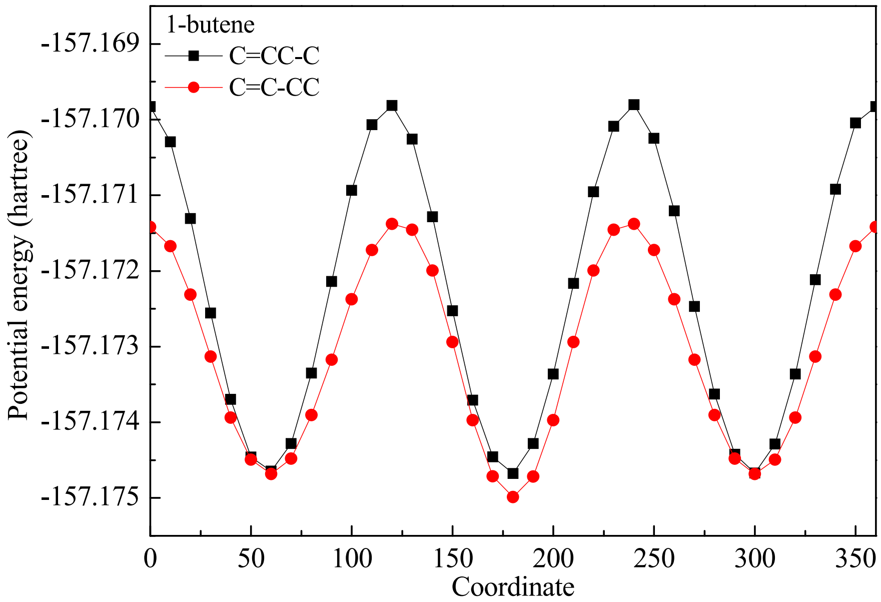

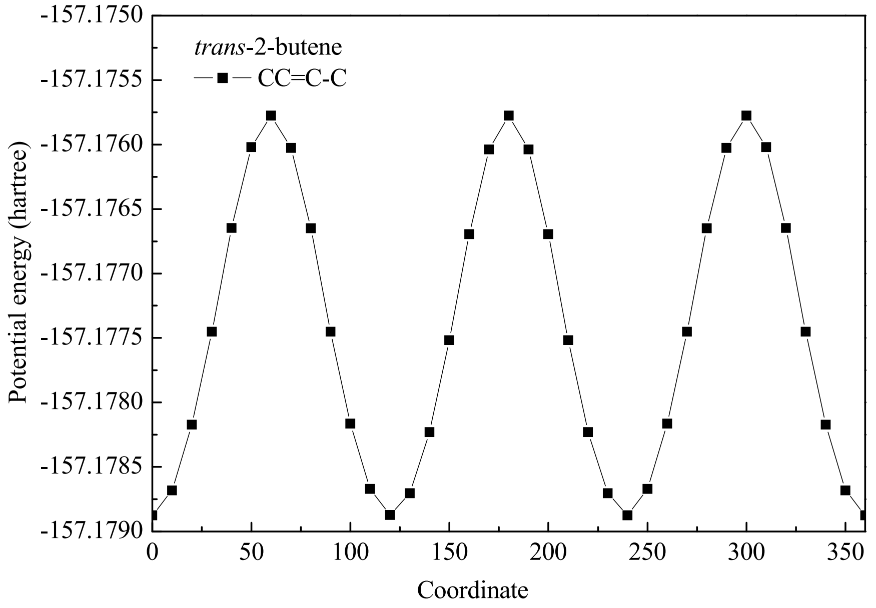
trans-2-butene
CC=C-C
Potential energy (hartree)
-157.1750
-157.1755
-157.1760
-157.1765
-157.1770
-157.1775
-157.1780
-157.1785
-157.1790
0
50
100
150
200
250
300
350
Coordinate

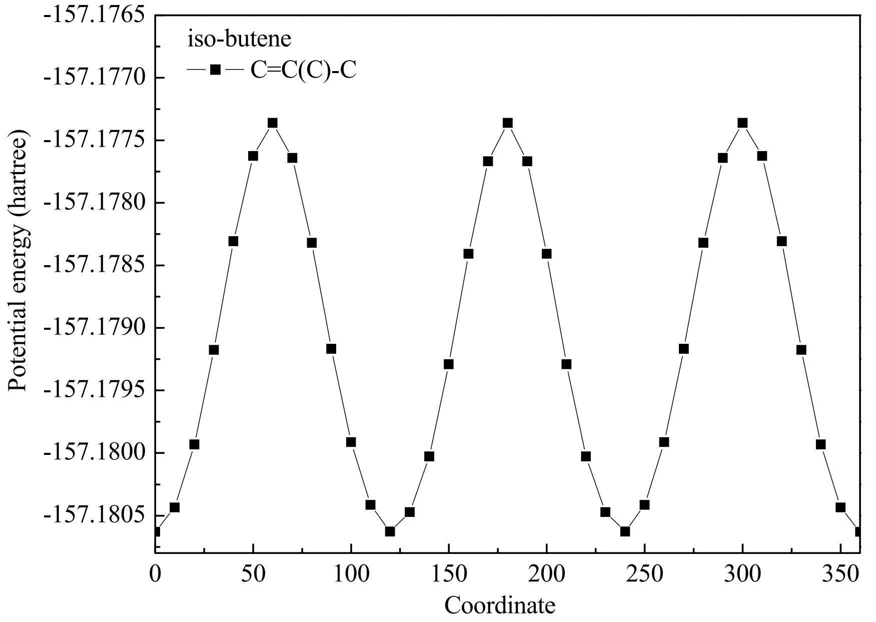
iso-butene
C=C(C)-C
Potential energy (hartree)
-157.1765
-157.1770
-157.1775
-157.1780
-157.1785
-157.1790
-157.1795
-157.1800
-157.1805
0
50
100
150
200
250
300
350
Coordinate

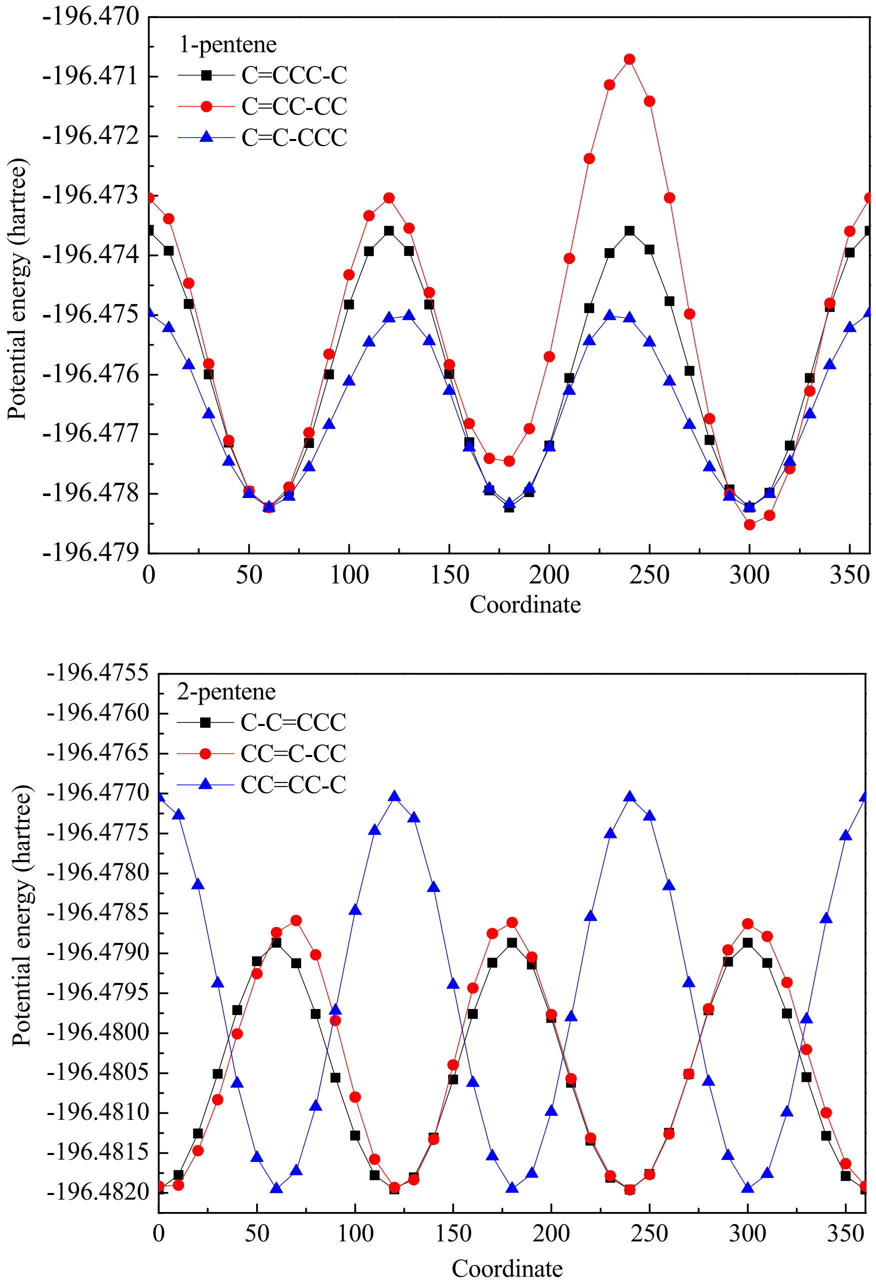

1-pentene
C=CCC-C
C=CC-CC
C=C-CCC
Potential energy (hartree)
Coordinate
2-pentene
C-C=CCC
CC=C-CC
CC=CC-C
Potential energy (hartree)
Coordinate

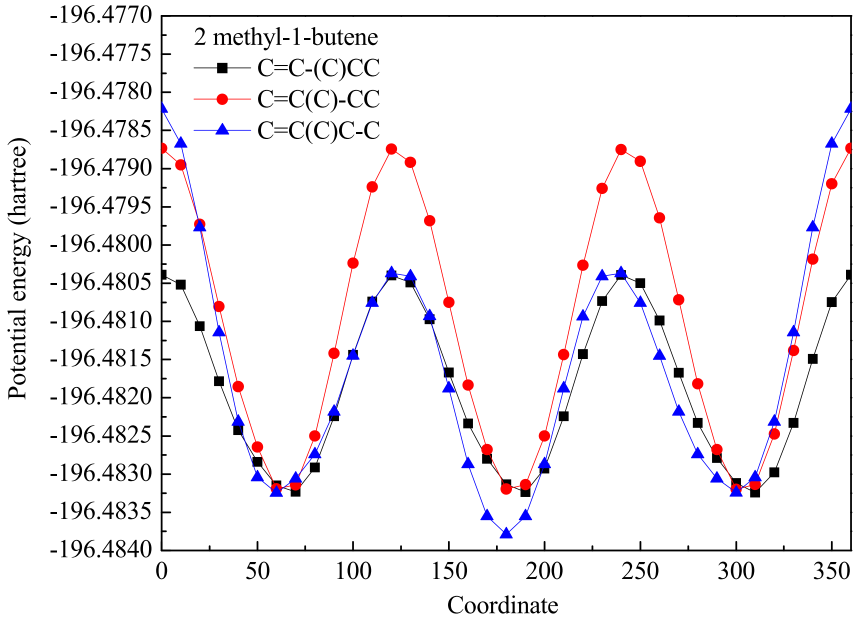

2 methyl-1-butene
C=C-(C)CC
C=C(C)-CC
C=C(C)C-C
Potential energy (hartree)
Coordinate
-196.4770
-196.4775
-196.4780
-196.4785
-196.4790
-196.4795
-196.4800
-196.4805
-196.4810
-196.4815
-196.4820
-196.4825
-196.4830
-196.4835
-196.4840
0
50
100
150
200
250
300
350

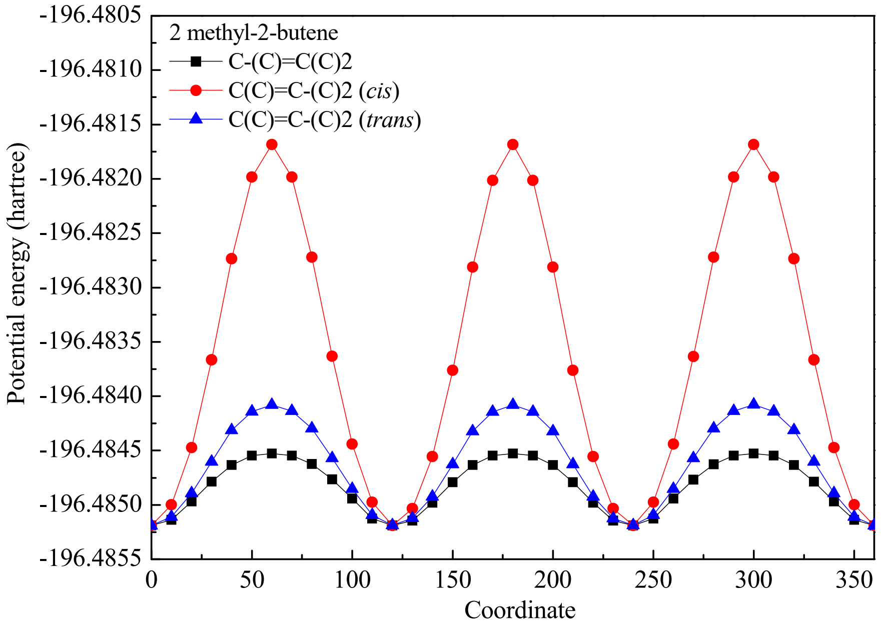

2 methyl-2-butene
C-(C)=C(C)2
C(C)=C-(C)2 (cis)
C(C)=C-(C)2 (trans)
Potential energy (hartree)
Coordinate
-196.4805
-196.4810
-196.4815
-196.4820
-196.4825
-196.4830
-196.4835
-196.4840
-196.4845
-196.4850
-196.4855
0
50
100
150
200
250
300
350

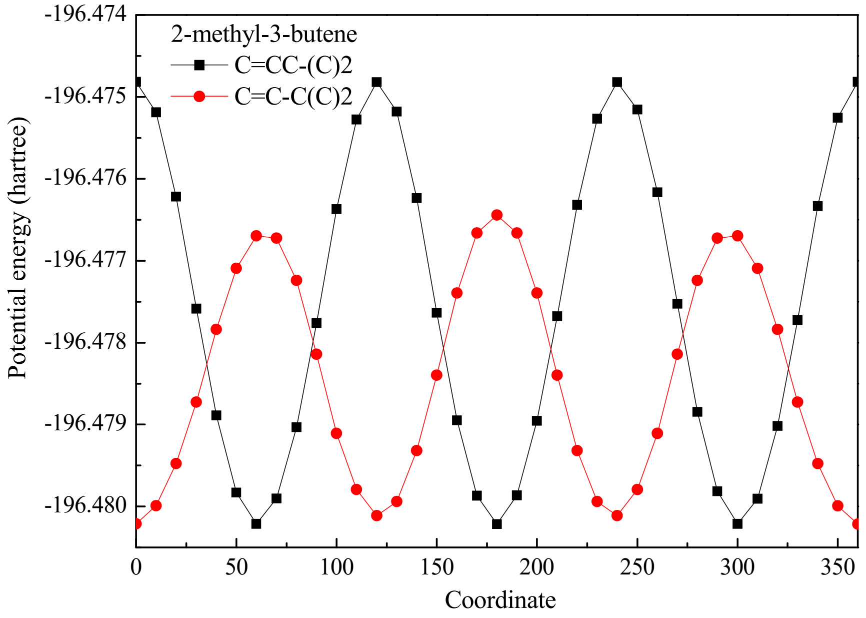

2-methyl-3-butene
C=CC-(C)2
C=C-C(C)2
Potential energy (hartree)
-196.474
-196.475
-196.476
-196.477
-196.478
-196.479
-196.480
0
50
100
150
200
250
300
350
Coordinate

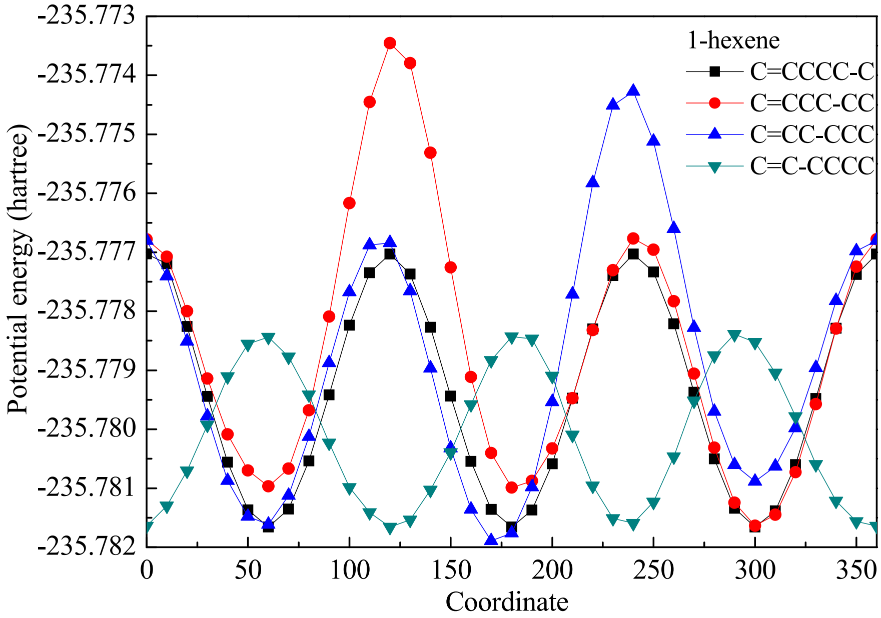

1-hexene
C=CCCC-C
C=CCC-CC
C=CC-CCC
C=C-CCCC
Potential energy (hartree)
-235.773
-235.774
-235.775
-235.776
-235.777
-235.778
-235.779
-235.780
-235.781
-235.782
0
50
100
150
200
250
300
350
Coordinate

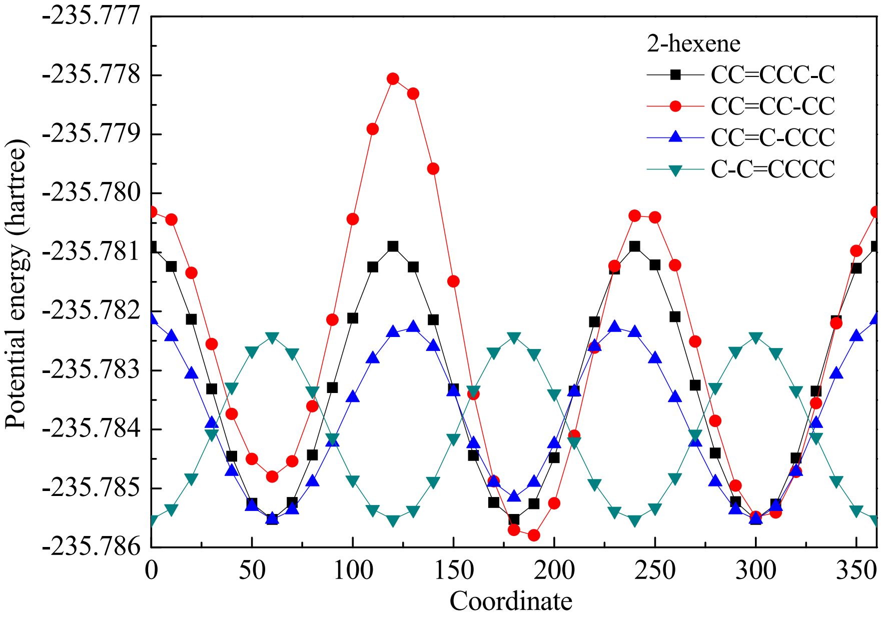
2-hexene
CC=CCC-C
CC=CC-CC
CC=C-CCC
C-C=CCCC
Potential energy (hartree)
-235.777
-235.778
-235.779
-235.780
-235.781
-235.782
-235.783
-235.784
-235.785
-235.786
0
50
100
150
200
250
300
350
Coordinate

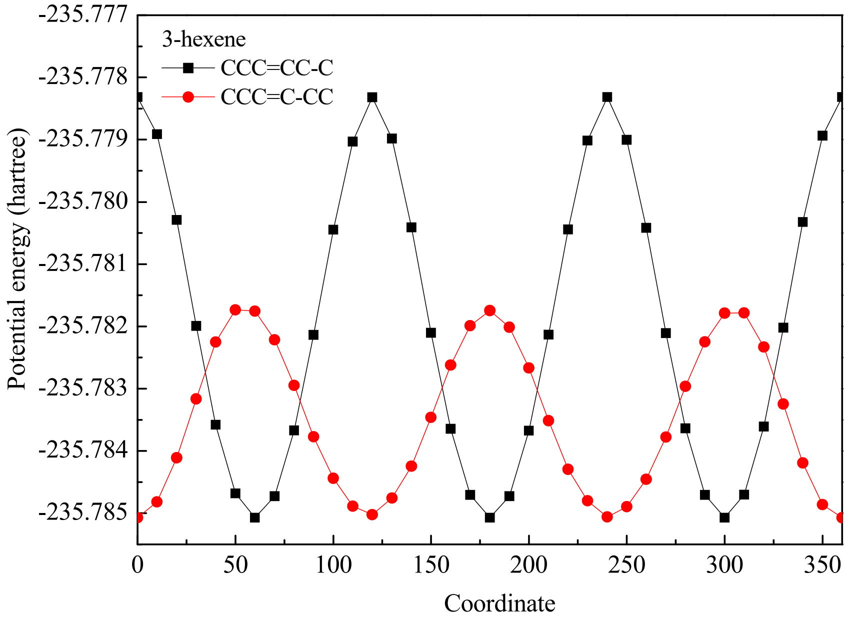
3-hexene
CCC=CC-C
CCC=C-CC
Potential energy (hartree)
-235.777
-235.778
-235.779
-235.780
-235.781
-235.782
-235.783
-235.784
-235.785
0
50
100
150
200
250
300
350
Coordinate

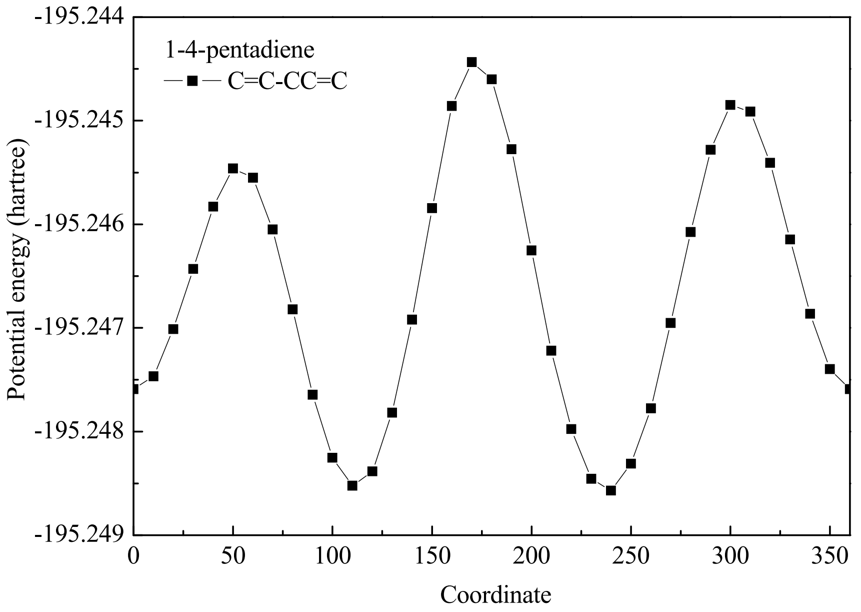

Figure S11 Hindrance potential analysis of reactants at M06-2X/6-311+G(d,p) level. The explicitly shown single C-C bond is the scanned internal rotors.

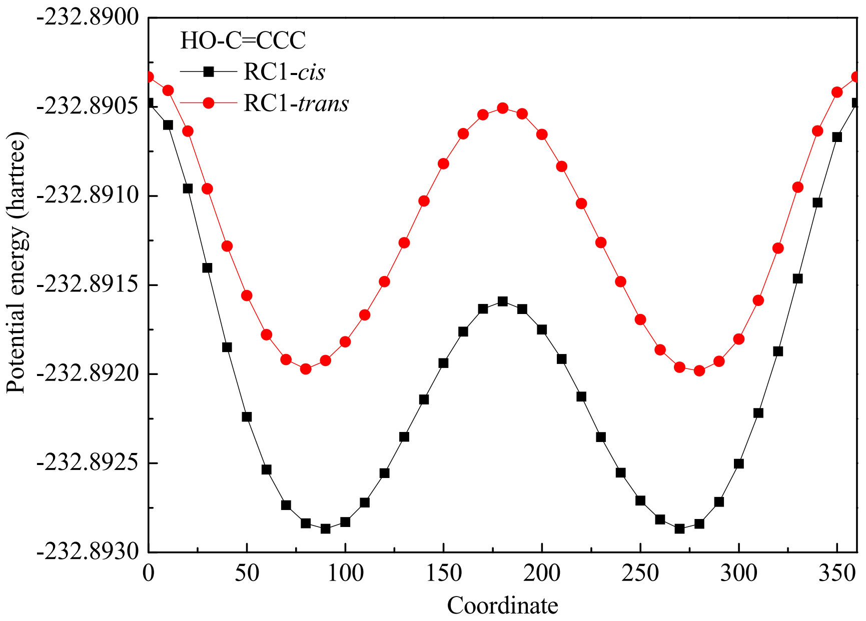

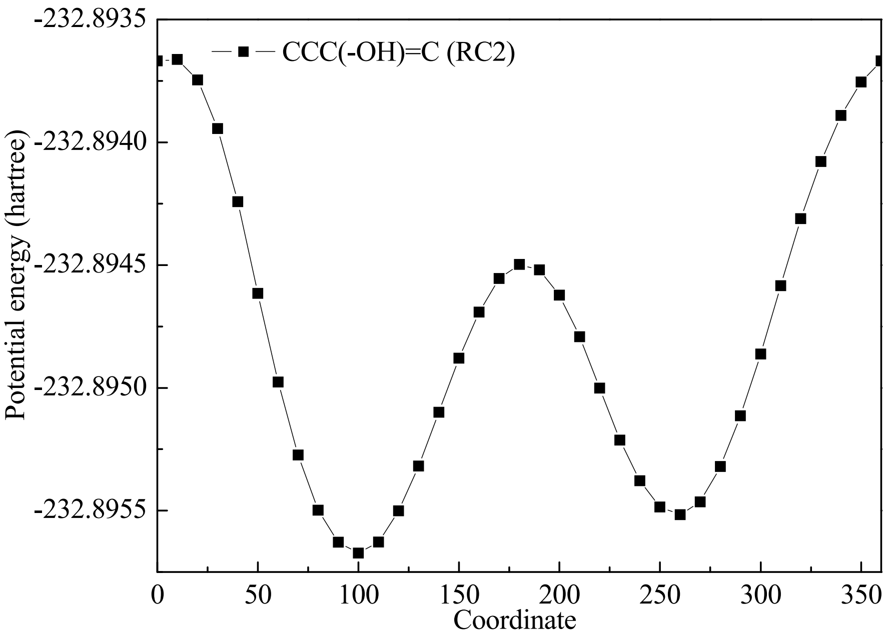
CCC(-OH)=C (RC2)
Potential energy (hartree)
-232.8935
-232.8940
-232.8945
-232.8950
-232.8955
0
50
100
150
200
250
300
350
Coordinate

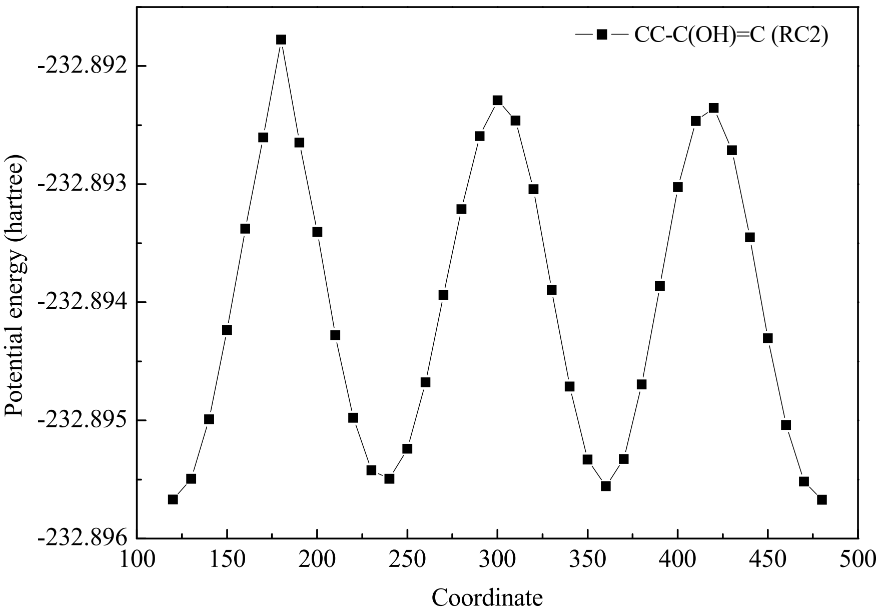
CC-C(OH)=C (RC2)
Potential energy (hartree)
-232.892
-232.893
-232.894
-232.895
-232.896
100
150
200
250
300
350
400
450
500
Coordinate

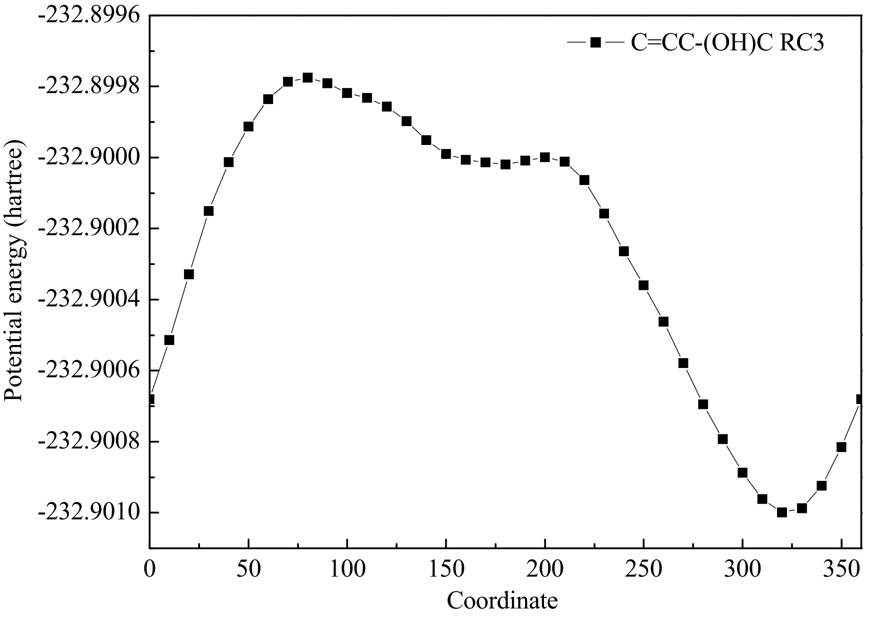

C=CC-(OH)C RC3
-232.8996
-232.8998
-232.9000
-232.9002
-232.9004
-232.9006
-232.9008
-232.9010
Potential energy (hartree)
0
50
100
150
200
250
300
350
Coordinate

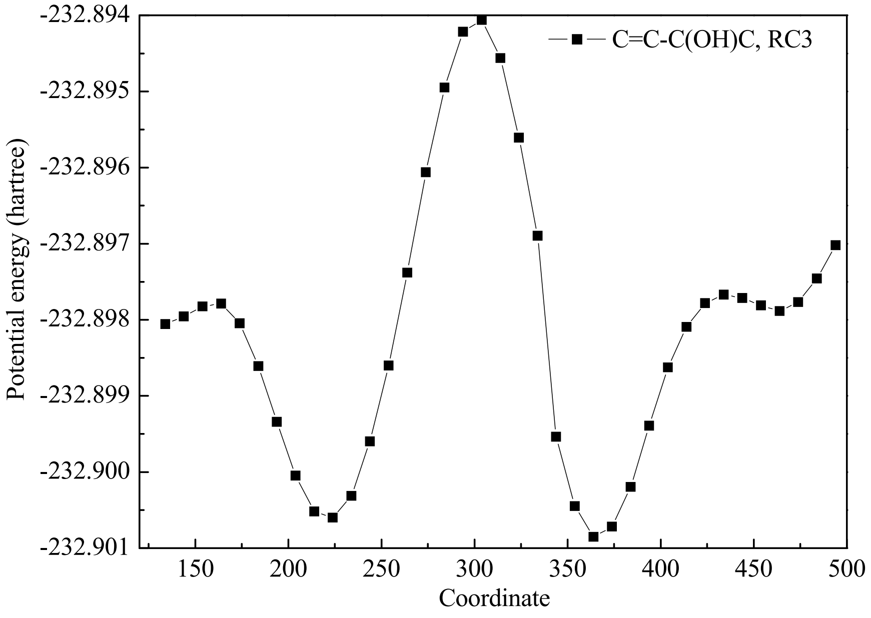

C=C-C(OH)C, RC3
-232.894
-232.895
-232.896
-232.897
-232.898
-232.899
-232.900
-232.901
Potential energy (hartree)
150
200
250
300
350
400
450
500
Coordinate

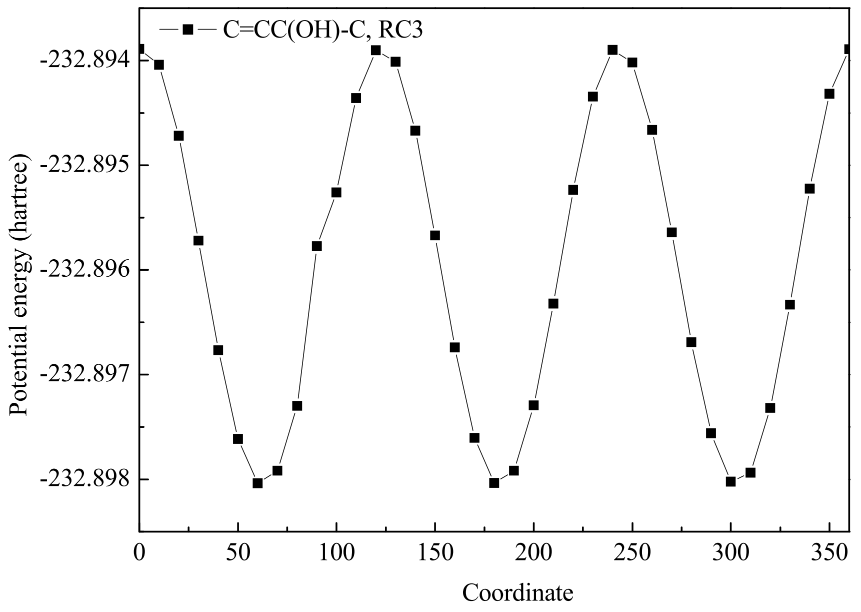
C=CC(OH)-C, RC3
Potential energy (hartree)
-232.894
-232.895
-232.896
-232.897
-232.898
0
50
100
150
200
250
300
350
Coordinate

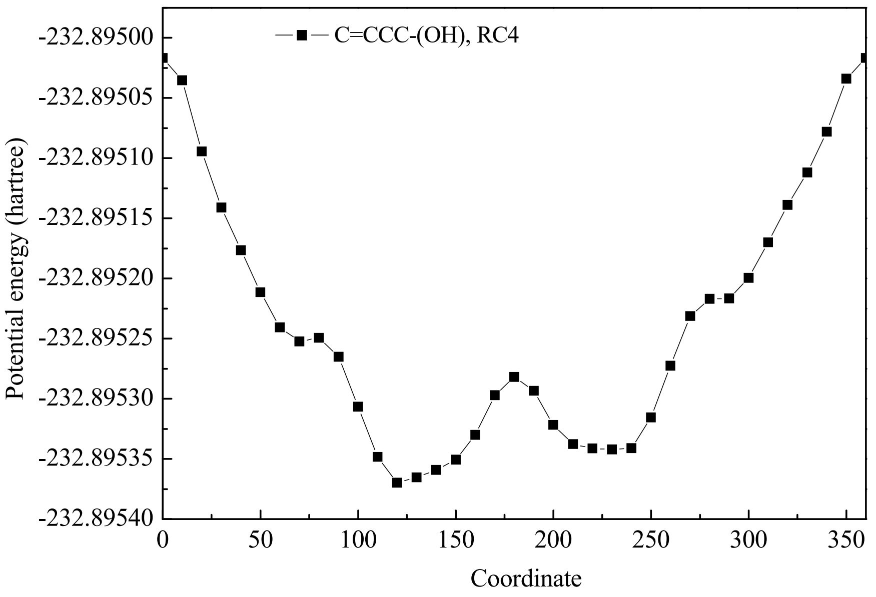
C=CCC-(OH), RC4
Potential energy (hartree)
-232.89500
-232.89505
-232.89510
-232.89515
-232.89520
-232.89525
-232.89530
-232.89535
-232.89540
0
50
100
150
200
250
300
350
Coordinate

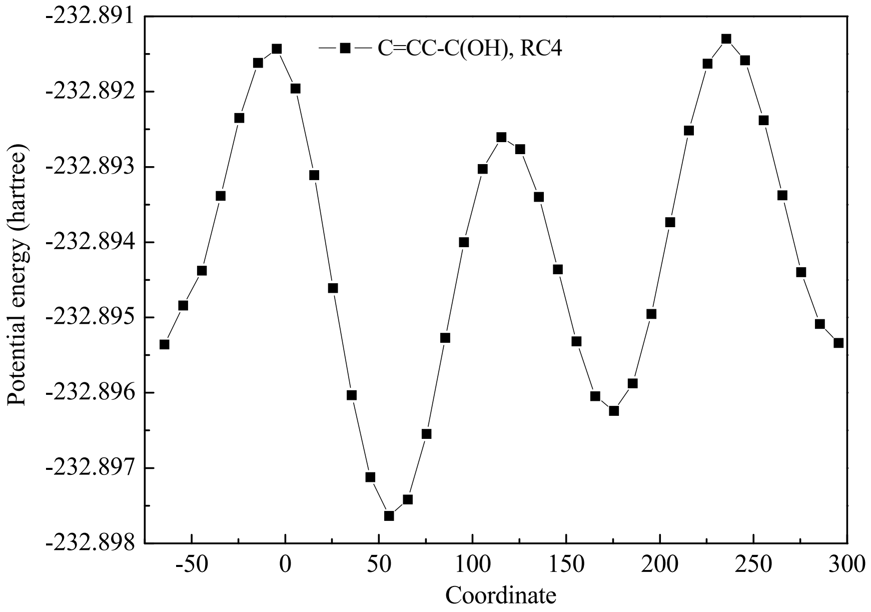

C=CC-C(OH), RC4
Potential energy (hartree)
-232.891
-232.892
-232.893
-232.894
-232.895
-232.896
-232.897
-232.898
-50
0
50
100
150
200
250
300
Coordinate

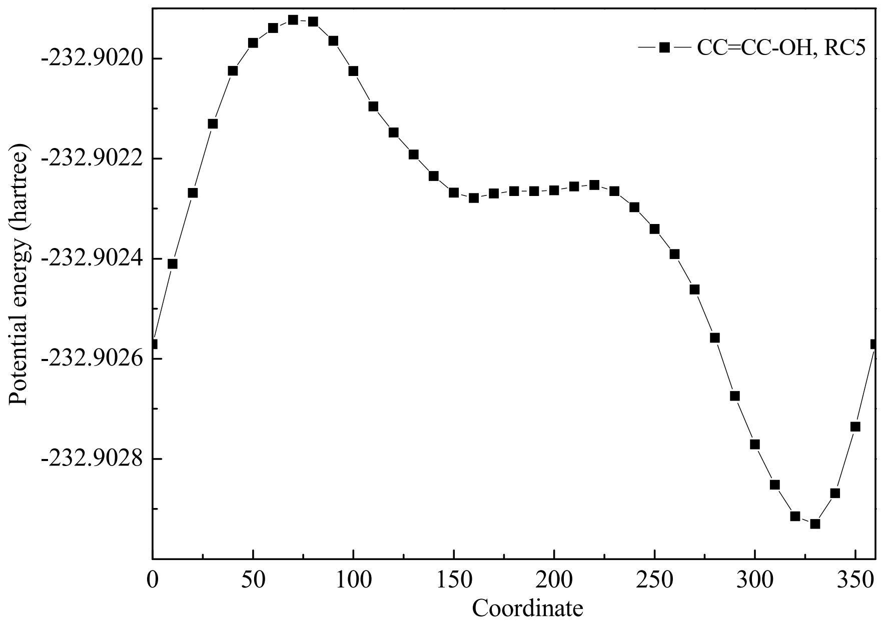

CC=CC-OH, RC5
Potential energy (hartree)
-232.9020
-232.9022
-232.9024
-232.9026
-232.9028
0
50
100
150
200
250
300
350
Coordinate

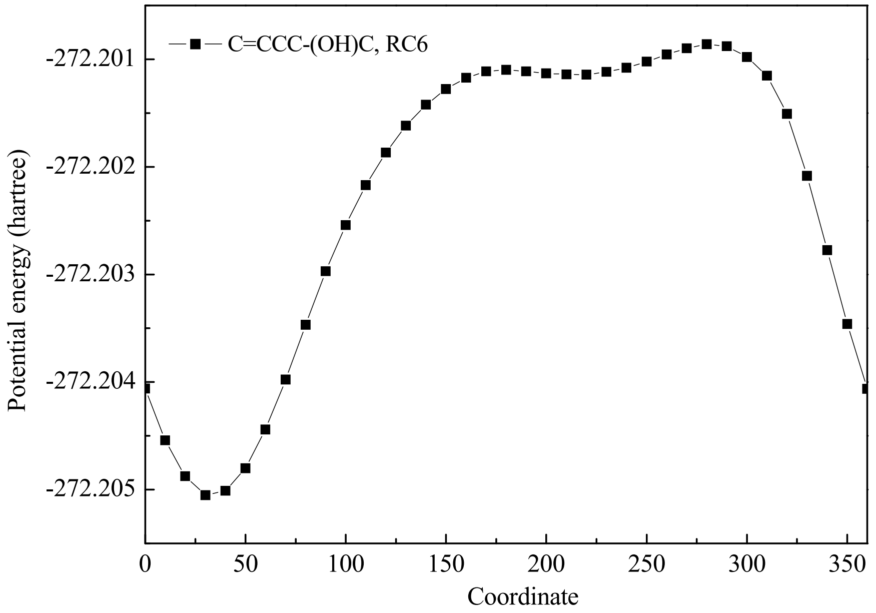
C=CCC-(OH)C, RC6
-272.201
-272.202
-272.203
-272.204
-272.205
Potential energy (hartree)
0
50
100
150
200
250
300
350
Coordinate

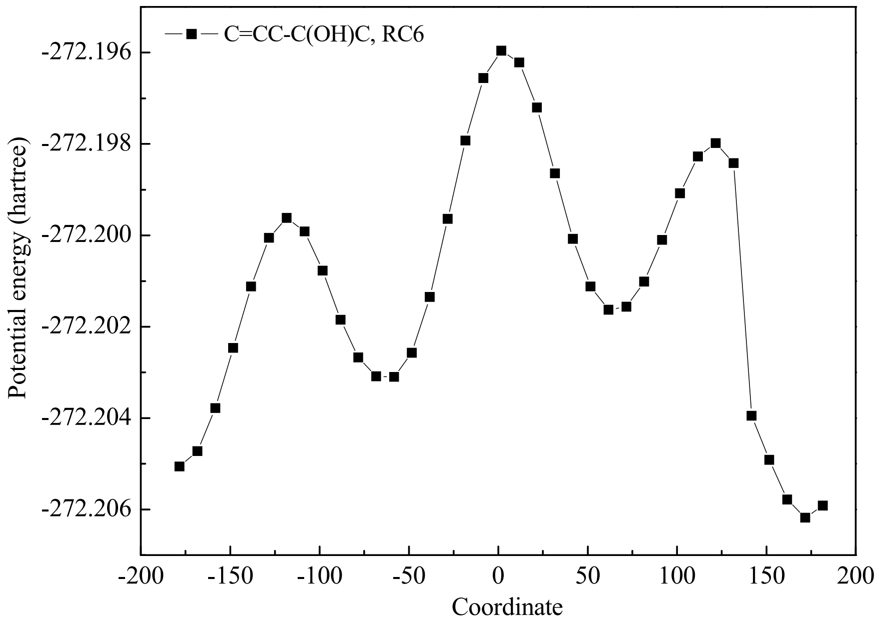
C=CC-C(OH)C, RC6
-272.196
-272.198
-272.200
-272.202
-272.204
-272.206
Potential energy (hartree)
-200
-150
-100
-50
0
50
100
150
200
Coordinate

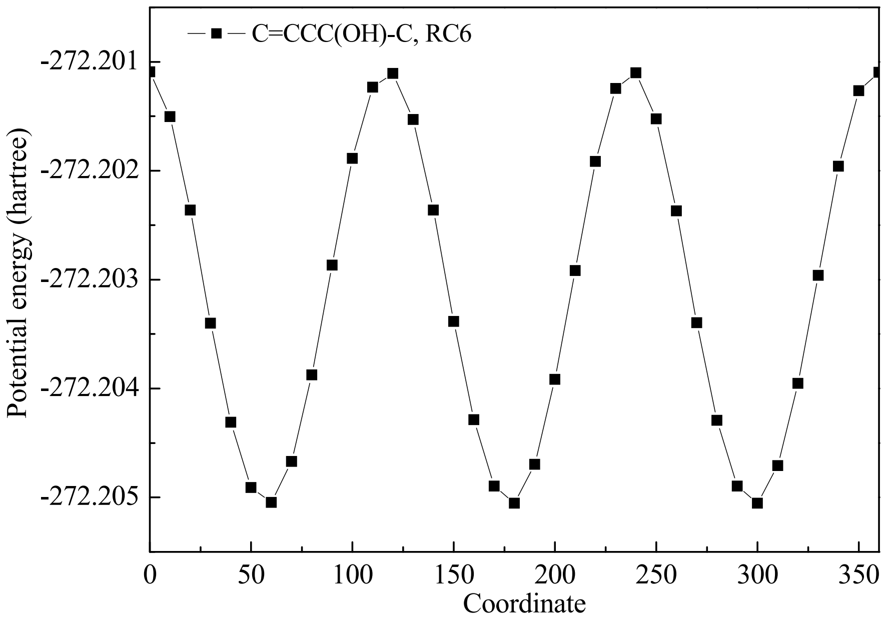
C=CCC(OH)-C, RC6
-272.201
-272.202
-272.203
-272.204
-272.205
0
50
100
150
200
250
300
350
Coordinate
Potential energy (hartree)

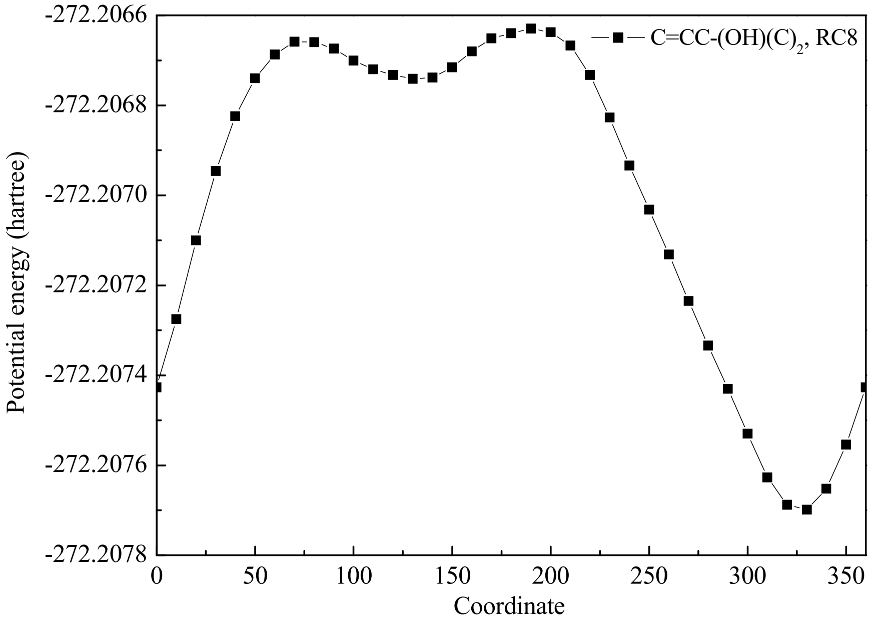
C=CC-(OH)(C)2, RC8
-272.2066
-272.2068
-272.2070
-272.2072
-272.2074
-272.2076
-272.2078
0
50
100
150
200
250
300
350
Coordinate
Potential energy (hartree)

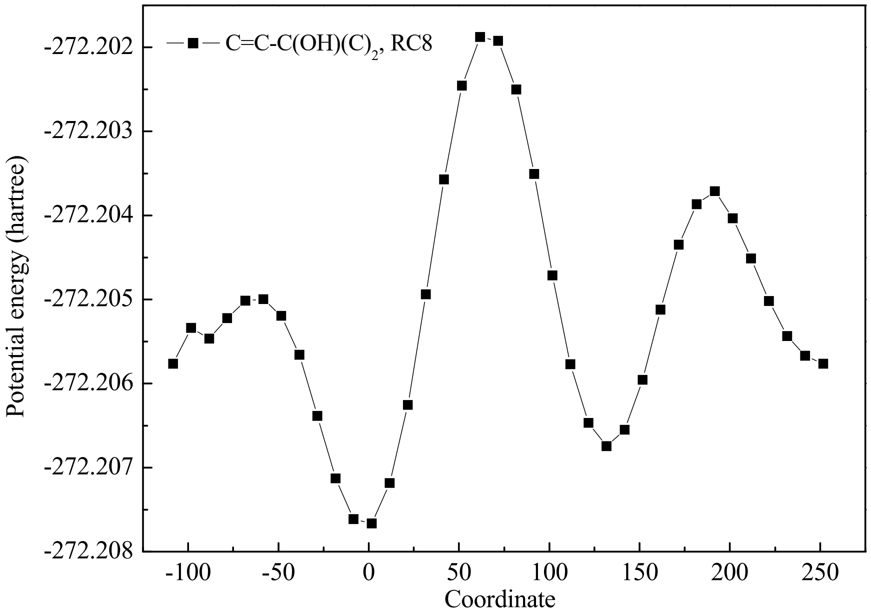
C=C-C(OH)(C)$_2$, RC8
Potential energy (hartree)
Coordinate
-272.202
-272.203
-272.204
-272.205
-272.206
-272.207
-272.208
-100
-50
0
50
100
150
200
250

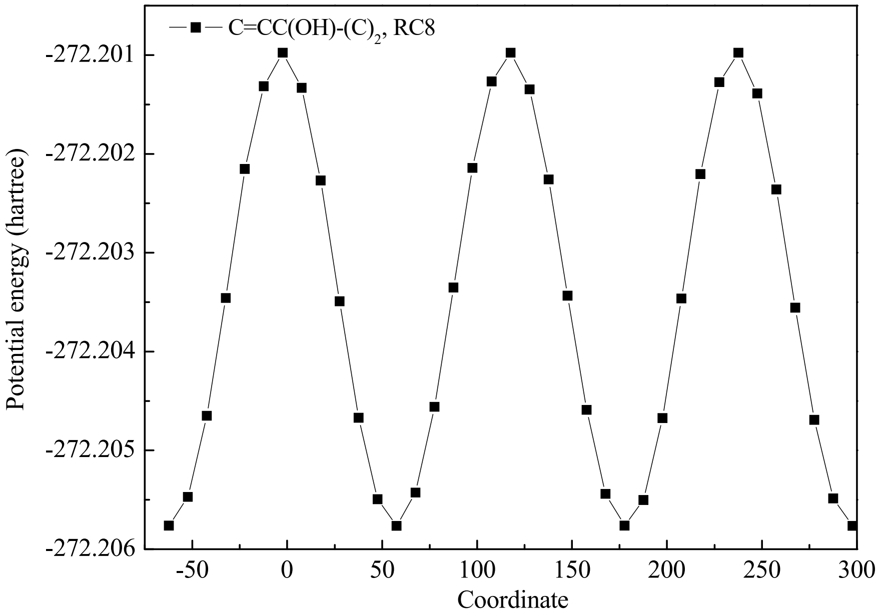
C=CC(OH)-(C)$_2$, RC8
Potential energy (hartree)
Coordinate
-272.201
-272.202
-272.203
-272.204
-272.205
-272.206
-50
0
50
100
150
200
250
300

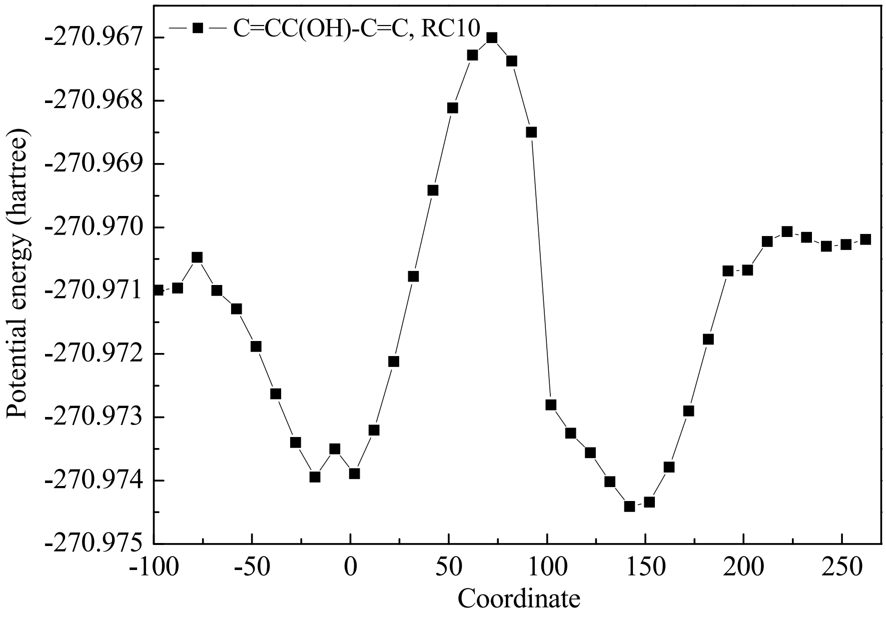

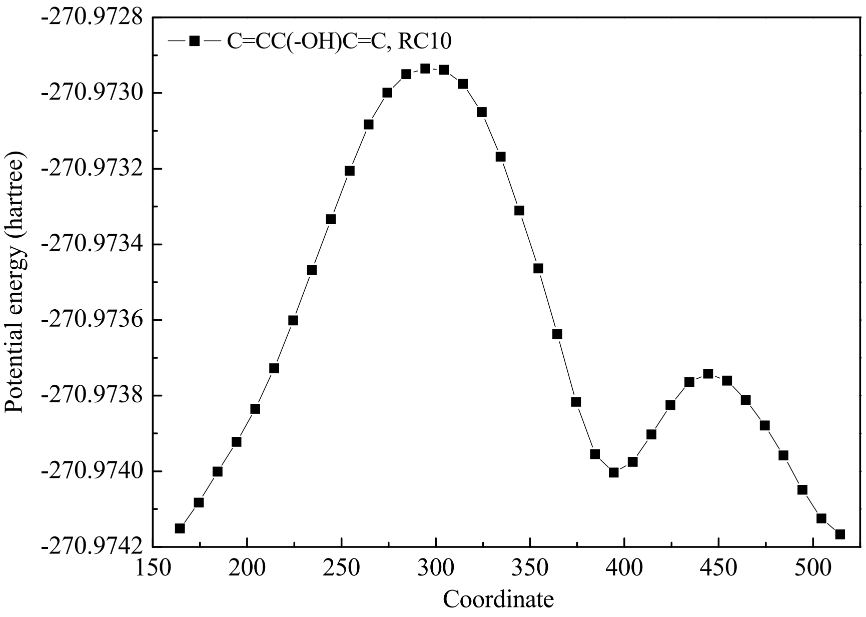

Figure S12 Hindrance potential analysis of TSs of prototype reactions at M06-2X/6-311+G(d,p) level. The explicitly shown single C-C/C-O bond is the scanned internal rotors. And the scanned results for prototype reactions were adopted for reactions within the same reaction classes since the relative potential energies are hardly affected.

Full list of the optimized geometries and frequencies for the studied abstraction reactions

Reactant	Geometry in Cartesian Coordinate (Å)				Frequency (cm^{-1})	Moment of inertia in au
1-butene	6	-1.711012	-0.245072	-0.296471	101.6424	80.93145
	6	-0.540230	0.529766	0.316392	227.2872	430.13440
	1	-2.624123	0.353299	-0.292611	319.5593	439.84766
	1	-1.487404	-0.525163	-1.327944	433.8483	

	1	-1.907607	-1.162454	0.264059	660.5609	
	6	0.714850	-0.292210	0.353730	794.0495	
	1	-0.356337	1.446356	-0.250968	869.5205	
	1	-0.804358	0.831417	1.335799	963.2479	
	6	1.838704	0.008656	-0.286944	984.1441	
	1	0.668321	-1.209004	0.940219	1033.4658	
	1	1.919411	0.911963	-0.884066	1046.6822	
	1	2.711978	-0.630847	-0.237202	1100.8308	
					1202.5711	
					1291.3676	
					1320.3301	
					1346.4855	
					1410.5137	
					1452.7314	
					1487.2332	
					1504.9515	
					1510.1560	
					1729.8315	
					3042.4602	
					3050.9200	
					3084.9277	
					3123.2699	
					3137.1327	
					3137.4992	
					3147.5322	
					3231.2601	
2-butene	6	-0.533497	-0.396441	-0.000019	162.9416	51.80555
	6	0.533497	0.396440	-0.000040	225.4364	482.69824
	6	1.955506	-0.078810	0.000014	241.8250	512.26120
	6	-1.955506	0.078810	0.000021	284.7486	
	1	-0.380851	-1.475004	-0.000059	505.7261	
	1	0.380850	1.475004	-0.000037	768.5711	
	1	2.005742	-1.168997	-0.001074	881.1894	
	1	2.492757	0.289516	-0.878319	984.8878	
	1	2.492066	0.287689	0.879548	1008.7412	
	1	-2.492372	-0.288409	-0.879019	1067.1266	
	1	-2.492450	-0.288795	0.878849	1076.9949	
	1	-2.005741	1.168998	0.000256	1096.4268	
					1171.6691	
					1328.9043	
					1338.7991	
					1414.6285	
					1416.0058	
					1483.9310	
					1484.4683	
					1495.0740	
					1503.0686	
					1772.7685	
					3038.3084	
					3038.5042	
					3097.0847	
					3097.5869	
					3122.2472	

					3123.1865	
					3137.3311	
					3144.0129	
isobutene	6	0.000000	1.456194	0.000000	190.8355	196.83213
	6	0.000000	0.125300	0.000000	235.4964	213.96331
	1	-0.925421	2.021469	-0.000115	383.6814	388.54983
	1	0.925421	2.021469	0.000115	436.3662	
	6	1.271161	-0.678280	0.000111	445.7548	
	6	-1.271161	-0.678280	-0.000110	712.7483	
	1	1.312700	-1.328853	-0.878994	832.9388	
	1	2.153713	-0.038247	0.000384	949.1420	
	1	1.312379	-1.329168	0.878993	971.0626	
	1	-1.312700	-1.328853	0.878994	993.8611	
	1	-2.153713	-0.038247	-0.000384	1019.2202	
	1	-1.312379	-1.329168	-0.878993	1087.7813	
					1107.7637	
					1308.4744	
					1411.2472	
					1418.2021	
					1447.5005	
					1481.9870	
					1489.9064	
					1501.4455	
					1508.2582	
					1741.9696	
					3046.3116	
					3049.9602	
					3102.9779	
					3105.1260	
					3150.1568	
					3150.4314	
					3153.2184	
					3239.4356	
1-pentene	6	0.000000	1.456194	0.000000	95.8605	99.71765
	6	0.000000	0.125300	0.000000	115.9711	844.71141
	1	-0.925421	2.021469	-0.000115	235.4737	857.04442
	1	0.925421	2.021469	0.000115	262.5383	
	6	1.271161	-0.678280	0.000111	388.2875	
	6	-1.271161	-0.678280	-0.000110	444.4945	
	1	1.312700	-1.328853	-0.878994	652.9062	
	1	2.153713	-0.038247	0.000384	750.6083	
	1	1.312379	-1.329168	0.878993	876.8940	
	1	-1.312700	-1.328853	0.878994	900.4082	
	1	-2.153713	-0.038247	-0.000384	963.7817	
	1	-1.312379	-1.329168	-0.878993	968.8264	
					1032.4109	
					1043.7560	
					1073.6858	
					1124.7205	
					1200.6362	
					1270.9678	
					1296.1836	
					1323.9922	

					1335.5089	
					1386.0016	
					1414.5793	
					1454.9052	
					1485.2019	
					1497.1503	
					1507.1816	
					1512.2986	
					1731.4924	
					3034.0260	
					3049.3694	
					3052.7794	
					3074.3662	
					3095.2432	
					3123.2240	
					3135.3214	
					3136.9355	
					3146.5680	
					3234.5744	
2-pentene	6	-1.299962	0.569201	-0.211710	74.5455	105.87031
	6	0.063014	-0.026704	-0.413513	171.2032	856.51535
	1	-1.234014	1.389067	0.509158	198.2118	881.28673
	1	-1.651424	1.002243	-1.154798	210.0986	
	6	1.160538	0.336928	0.243473	303.8810	
	6	2.517424	-0.272332	0.051919	411.9276	
	1	2.898149	-0.686111	0.989846	493.1431	
	1	2.486329	-1.073516	-0.688577	767.9028	
	1	3.239914	0.476914	-0.283958	815.1629	
	6	-2.315323	-0.472691	0.268177	893.6703	
	1	-2.006285	-0.895902	1.226263	961.3922	
	1	-3.307152	-0.032350	0.389670	1000.5908	
	1	-2.397367	-1.293625	-0.448753	1038.0231	
	1	1.085439	1.139191	0.976712	1073.9285	
	1	0.132270	-0.832326	-1.145639	1093.8346	
					1113.5782	
					1183.9341	
					1276.3768	
					1319.4154	
					1338.7921	
					1372.4157	
					1413.2358	
					1417.8432	
					1486.1796	
					1488.5230	
					1500.8908	
					1504.6566	
					1511.4029	
					1764.5446	
					3043.5279	
					3046.2726	
					3050.6010	
					3086.0919	
					3106.6752	

					3119.1723	
					3120.9669	
					3124.9024	
					3135.2804	
					3138.7771	
2-methyl-1-butene	6	0.687663	-0.762222	-0.000051	86.2380	209.24117
	6	2.029684	-0.041072	0.000116	175.9396	507.07759
	6	-0.536607	0.120838	-0.000048	253.7693	683.01359
	6	-1.845088	-0.623299	0.000043	288.1101	
	6	-0.485141	1.451310	-0.000098	408.3976	
	1	0.624303	-1.425597	-0.871539	435.2483	
	1	0.624181	-1.425782	0.871286	501.4490	
	1	2.135639	0.591950	0.883887	692.8510	
	1	2.851159	-0.759270	0.000150	796.3949	
	1	2.135815	0.592051	-0.883562	806.9664	
	1	-1.918401	-1.270543	0.879533	948.5755	
	1	-2.696415	0.057704	-0.000221	970.2056	
	1	-1.918259	-1.271070	-0.879066	1008.7212	
	1	-1.393590	2.043461	-0.000119	1038.0909	
	1	0.452500	1.993769	-0.000117	1042.1294	
					1108.4813	
					1123.7528	
					1264.5395	
					1299.8068	
					1391.7990	
					1411.1785	
					1414.1981	
					1454.7837	
					1476.1701	
					1488.0342	
					1498.6320	
					1500.8205	
					1514.9249	
					1738.5454	
					3031.1726	
					3040.8059	
					3045.1109	
					3058.1288	
					3097.3233	
					3119.4747	
					3123.4938	
					3139.8294	
					3161.5850	
					3245.6399	
2-methyl-2-butene	6	0.731745	-0.675012	-0.000004	113.2688	223.05408
	6	-0.444689	-0.043657	-0.000001	144.9992	512.39902
	1	0.708050	-1.763305	0.000005	205.0914	702.11935
	6	-1.738408	-0.815613	0.000016	279.9923	
	6	-0.624334	1.450729	-0.000002	306.7965	
	6	2.106856	-0.074425	-0.000013	394.0819	
	1	-2.339414	-0.561079	-0.879039	460.4714	
	1	-1.566261	-1.892401	0.000036	525.8014	
	1	-2.339413	-0.561045	0.879062	783.5712	

	1	-1.200079	1.759623	0.878377	841.8968	
	1	0.314568	2.000525	-0.000086	970.0405	
	1	-1.200230	1.759606	-0.878287	981.2769	
	1	2.096838	1.014369	-0.000165	1022.4455	
	1	2.669417	-0.404093	0.878288	1066.3268	
	1	2.669503	-0.404335	-0.878168	1075.6915	
					1110.3829	
					1144.0809	
					1247.2966	
					1370.8023	
					1418.2400	
					1426.1320	
					1429.8802	
					1478.8725	
					1488.8306	
					1489.9332	
					1500.6236	
					1505.7009	
					1506.2924	
					1769.1931	
					3046.5751	
					3047.7940	
					3052.4463	
					3099.6186	
					3102.1633	
					3104.5585	
					3129.2266	
					3137.6835	
					3147.7173	
					3165.3874	
2-methyl-3-butene	6	2.141611	-0.000005	-0.159675	91.6369	238.31384
	6	0.943760	-0.000018	0.413808	235.6090	506.54901
	6	-0.369717	0.000000	-0.317444	259.0470	654.74316
	6	-1.171094	-1.259468	0.029240	323.3383	
	6	-1.171060	1.259486	0.029253	327.4648	
	1	3.053039	-0.000017	0.426421	350.3100	
	1	2.248056	0.000015	-1.240227	509.0346	
	1	0.872674	-0.000041	1.502231	706.7902	
	1	-0.160832	0.000004	-1.392833	817.1788	
	1	-2.127528	-1.268211	-0.499325	933.2219	
	1	-0.619229	-2.162940	-0.236928	951.9346	
	1	-1.380275	-1.293784	1.102887	969.4605	
	1	-2.127498	1.268256	-0.499304	971.4549	
	1	-1.380231	1.293801	1.102903	1004.4944	
	1	-0.619173	2.162945	-0.236915	1041.8324	
					1137.8643	
					1190.5855	
					1227.9769	
					1314.7478	
					1337.6837	
					1345.6298	
					1398.0691	
					1417.8399	

					1458.9901	
					1491.3448	
					1493.0385	
					1505.2902	
					1516.8128	
					1724.8307	
					3039.4377	
					3041.4840	
					3052.6275	
					3108.8774	
					3114.0611	
					3126.5941	
					3132.0161	
					3133.5912	
					3150.5687	
					3240.5087	
1-hexene	6	3.067003	-0.187521	-0.432351	74.4295	133.56733
	6	2.027925	-0.197497	0.394883	85.5322	1438.25541
	6	0.769828	0.597336	0.207638	116.4740	1466.31773
	6	-0.469622	-0.294979	0.084584	180.8014	
	6	-1.765704	0.503290	-0.032339	237.3156	
	1	-0.528420	-0.955742	0.957933	359.0686	
	1	-0.356611	-0.947079	-0.788831	363.0811	
	1	0.861676	1.229641	-0.681392	461.8950	
	1	0.634820	1.268653	1.064764	655.5718	
	1	3.945574	-0.794376	-0.248247	728.6945	
	1	3.071381	0.433964	-1.322763	789.4191	
	1	2.059362	-0.836430	1.276537	911.6834	
	6	-2.994626	-0.393004	-0.160190	923.2000	
	1	-1.868804	1.149657	0.846227	948.4827	
	1	-1.702370	1.169409	-0.899681	967.4608	
	1	-3.082663	-1.056836	0.703885	1034.7129	
	1	-3.913643	0.192493	-0.227928	1045.8867	
	1	-2.929131	-1.019105	-1.053849	1058.9032	
					1086.0752	
					1132.8550	
					1199.3182	
					1245.4374	
					1264.8855	
					1309.0883	
					1323.6991	
					1334.7300	
					1351.3859	
					1399.8944	
					1411.8751	
					1455.1785	
					1482.4313	
					1488.8675	
					1499.5659	
					1504.1671	
					1511.6602	
					1731.8863	
					3020.0154	

					3028.7583	
					3038.3031	
					3044.1172	
					3057.6257	
					3072.0173	
					3088.4408	
					3115.8559	
					3123.0094	
					3143.0175	
					3156.6638	
					3233.8083	
2-hexene	6	-0.646202	-0.487057	0.369316	75.5212	121.88199
	6	-1.666723	0.527976	-0.157896	103.8980	1485.35428
	6	0.739569	0.081461	0.454747	135.8650	1502.96770
	6	1.789306	-0.358061	-0.233400	210.9211	
	6	3.169602	0.223313	-0.159445	263.5092	
	1	-0.968410	-0.822323	1.362980	291.3763	
	1	-0.641820	-1.371624	-0.276367	315.5287	
	1	3.201400	1.073586	0.524081	391.5071	
	1	3.506906	0.561149	-1.143205	539.0921	
	1	3.890814	-0.522167	0.187405	750.1988	
	1	1.653745	-1.206135	-0.903688	791.0000	
	1	0.870147	0.932263	1.124479	881.8973	
	6	-3.081420	-0.043093	-0.200515	912.6899	
	1	-1.645853	1.421240	0.475573	933.9131	
	1	-1.359792	0.850079	-1.157619	1001.6436	
	1	-3.410818	-0.342552	0.797918	1046.4043	
	1	-3.796804	0.686114	-0.585690	1068.3702	
	1	-3.124314	-0.926860	-0.842715	1074.1607	
					1109.9041	
					1124.2449	
					1185.0524	
					1255.9426	
					1292.8943	
					1320.6423	
					1337.7595	
					1345.1622	
					1395.2368	
					1417.8169	
					1419.3610	
					1483.2157	
					1484.6984	
					1497.9649	
					1501.8359	
					1508.0125	
					1512.9686	
					1762.8275	
					3030.0446	
					3044.8470	
					3049.8462	
					3053.7817	
					3076.8489	
					3095.1640	

					3104.1888	
					3115.1674	
					3119.5345	
					3122.0710	
					3136.2986	
					3136.5492	
3-hexene	6	-0.545633	0.038156	-0.378243	45.8147	136.58613
	6	0.545637	-0.038155	0.378260	119.7802	1425.11175
	6	1.849538	0.646658	0.087853	131.9793	1466.41664
	6	-1.849535	-0.646657	-0.087838	193.0609	
	6	2.989978	-0.356997	-0.111131	235.8848	
	6	-2.989984	0.356995	0.111104	329.1493	
	1	-0.520263	0.660457	-1.273904	331.0643	
	1	0.520266	-0.660455	1.273921	475.5302	
	1	1.741795	1.273651	-0.801712	489.8937	
	1	2.102105	1.313921	0.919265	757.4784	
	1	-2.102084	-1.313942	-0.919236	789.3272	
	1	-1.741800	-1.273628	0.801744	856.3357	
	1	3.939144	0.151841	-0.290865	909.2576	
	1	3.108819	-0.988790	0.772819	920.3738	
	1	2.781745	-1.009330	-0.961760	1013.3512	
	1	-3.939149	-0.151845	0.290840	1029.1063	
	1	-2.781767	1.009354	0.961717	1052.4287	
	1	-3.108819	0.988761	-0.772866	1096.3308	
					1098.2396	
					1127.5627	
					1189.5654	
					1261.6309	
					1282.4783	
					1324.0007	
					1334.3096	
					1348.6780	
					1386.8304	
					1405.6256	
					1405.7128	
					1484.0414	
					1484.9001	
					1500.0232	
					1500.4135	
					1510.2778	
					1510.7054	
					1765.4055	
					3037.7852	
					3038.5750	
					3042.8586	
					3042.9894	
					3079.2534	
					3080.1548	
					3116.3265	
					3116.4941	
					3128.7798	
					3128.8103	
					3136.6914	

					3141.6972	
1-4-pentadiene	6	-1.161245	-0.170178	-0.440714	78.4819	92.36139
	6	0.000001	0.681093	0.000053	103.8898	758.27482
	6	1.161242	-0.170259	0.440684	310.0265	764.66957
	6	-2.350292	-0.189684	0.150024	376.0552	
	6	2.350293	-0.189659	-0.150051	464.1876	
	1	-0.972180	-0.820398	-1.292367	624.0432	
	1	-0.321628	1.326979	0.823540	692.7247	
	1	0.321637	1.327109	-0.823329	902.0598	
	1	0.972174	-0.820624	1.292225	921.6550	
	1	-3.147666	-0.833484	-0.201465	969.8678	
	1	-2.565055	0.442938	1.005948	973.3578	
	1	3.147669	-0.833514	0.201332	978.9659	
	1	2.565060	0.443121	-1.005858	1036.2356	
					1043.4624	
					1092.6578	
					1177.4663	
					1268.1571	
					1299.8277	
					1316.7438	
					1330.2337	
					1444.0087	
					1450.1203	
					1482.1004	
					1715.5980	
					1729.2376	
					3042.5977	
					3089.1151	
					3140.1551	
					3140.3073	
					3151.3658	
					3151.4038	
					3233.4708	
					3233.5011	

**Reaction**	**TS Geometry** in Cartesian Coordinate (Å)				**Frequency(cm^{-1})**	Moment of inertia in au
**R1-*cis***	6	2.054844	-0.627238	-0.385781	1211.5088i	276.01942
	6	0.995137	-0.155067	0.614448	46.5608	710.97445
	1	2.599342	-1.489209	0.003505	127.3655	893.16927
	1	1.591134	-0.914216	-1.331974	177.3043	
	1	2.779199	0.164159	-0.593715	179.0615	
	6	0.263480	1.056425	0.111692	228.8173	
	1	0.274683	-0.950810	0.815254	325.2427	
	1	1.480813	0.096398	1.562893	457.3392	
	6	-1.028903	1.119139	-0.150889	564.2299	
	1	0.865715	1.952025	-0.056772	717.6837	
	1	-1.704524	0.144849	0.064515	794.6701	
	1	-1.582967	1.964631	-0.541782	831.2832	
	8	-2.236048	-1.048590	-0.036478	886.2411	
	1	-2.036386	-1.228482	-0.968035	946.1188	
					971.3622	
					1042.5263	

					1094.4423	
					1177.4391	
					1241.0423	
					1279.3512	
					1290.6602	
					1320.1767	
					1377.1448	
					1408.2057	
					1491.4144	
					1501.1677	
					1508.1471	
					1711.8519	
					3041.7602	
					3049.1099	
					3098.1221	
					3112.3423	
					3123.5423	
					3126.6618	
					3194.1528	
					3819.9039	
**R1-*trans***	6	-2.497394	-0.521346	-0.286053	1231.0048i	116.04582
	6	-1.519348	0.460643	0.364448	87.5902	1150.86449
	1	-3.525981	-0.169825	-0.188113	100.4571	1188.84023
	1	-2.273815	-0.640565	-1.347849	119.4635	
	1	-2.430049	-1.505726	0.183225	190.4102	
	6	-0.100156	-0.034199	0.278630	243.4918	
	1	-1.595201	1.443007	-0.108256	348.2205	
	1	-1.780634	0.589691	1.420265	367.8080	
	6	0.878521	0.596581	-0.343399	559.4728	
	1	0.105255	-0.997903	0.743677	751.4940	
	1	0.828199	1.560842	-0.839573	803.3339	
	1	1.958655	0.066752	-0.392141	858.6854	
	8	3.116342	-0.420519	-0.046338	876.0110	
	1	3.213098	0.007805	0.817720	961.7954	
					979.4901	
					1041.8441	
					1096.0492	
					1174.0997	
					1241.6547	
					1286.3287	
					1299.9070	
					1315.0833	
					1359.9507	
					1412.6561	
					1483.9486	
					1503.3797	
					1509.2953	
					1722.5503	
					3042.8533	
					3051.2675	
					3095.8405	
					3128.5967	
					3132.9535	

					3144.7677	
					3176.7923	
					3830.6197	
**R2**	6	1.778147	-0.577511	0.371283	907.4206i	401.65432
	6	1.077774	0.478493	-0.487989	69.6300	511.93184
	1	2.840489	-0.635108	0.128846	95.5269	829.82339
	1	1.680582	-0.336836	1.432102	162.9524	
	1	1.334920	-1.560850	0.200547	216.7142	
	6	-0.382259	0.563632	-0.184681	251.3279	
	1	1.523825	1.465805	-0.326197	344.7047	
	1	1.208674	0.232207	-1.546255	406.9292	
	6	-1.100320	1.584116	0.250747	601.4363	
	1	-0.955979	-0.456886	-0.352747	726.0417	
	1	-0.645713	2.559826	0.405651	792.2218	
	1	-2.160987	1.489206	0.451365	841.5005	
	8	-1.524685	-1.673556	-0.107203	898.7235	
	1	-1.467878	-1.666515	0.861267	952.1813	
					980.1547	
					1042.2652	
					1097.6834	
					1176.8421	
					1218.2947	
					1280.6469	
					1328.4198	
					1404.1037	
					1420.7574	
					1435.6329	
					1475.9178	
					1503.3314	
					1514.7488	
					1731.5122	
					3039.0828	
					3050.3822	
					3082.3690	
					3127.1664	
					3128.0931	
					3134.1926	
					3226.7424	
					3810.1106	
**R3**	6	-2.090201	-0.634772	0.118972	665.5269i	398.97317
	6	-0.982691	-0.110659	-0.400086	53.7932	570.40165
	6	0.146470	0.445729	0.396964	84.4409	884.84747
	1	1.033376	-0.279943	0.294394	124.7187	
	1	-0.090102	0.453021	1.464002	191.6347	
	1	-2.243085	-0.677263	1.192919	218.8023	
	8	1.903430	-1.462815	-0.041436	309.0207	
	1	1.167917	-2.094598	-0.098514	444.4220	
	1	-2.879813	-1.028628	-0.509559	592.1244	
	1	-0.862551	-0.078210	-1.482037	688.2907	
	6	0.648235	1.797517	-0.093948	801.8506	
	1	-0.135674	2.553056	0.007193	876.8633	
	1	1.517287	2.124357	0.478271	972.0328	
	1	0.934331	1.743836	-1.146601	987.6710	

					1021.1896	
					1050.3812	
					1087.0430	
					1202.0628	
					1233.5468	
					1315.5046	
					1333.9727	
					1365.8899	
					1412.8087	
					1460.3242	
					1499.2983	
					1500.8068	
					1627.4905	
					1736.0638	
					3054.2278	
					3097.5406	
					3127.2787	
					3140.0530	
					3144.0991	
					3149.6288	
					3234.5925	
					3797.8052	
**R4**	6	-0.029058	-0.026287	-0.032885	744.3069i	111.28362
	6	0.053092	0.000538	1.485582	46.5238	1163.37530
	1	1.071100	-0.052031	-0.467386	64.3170	1188.37800
	1	-0.514869	-0.921041	-0.425633	109.1832	
	1	-0.470847	0.874230	-0.460422	176.1663	
	6	0.710719	-1.233019	2.033424	296.1976	
	1	0.599732	0.888473	1.814289	332.2264	
	1	-0.962765	0.077606	1.893059	431.4940	
	6	1.842518	-1.238934	2.731188	660.0434	
	1	0.218150	-2.178379	1.810423	711.2348	
	1	2.358455	-0.314190	2.972811	812.4630	
	1	2.281535	-2.161008	3.093482	917.5260	
	8	2.428859	-0.231191	-0.598566	972.7016	
	1	2.554318	-0.785389	0.190469	981.9995	
					1037.0990	
					1053.5159	
					1074.3208	
					1162.2058	
					1226.6682	
					1282.3526	
					1307.2209	
					1327.8378	
					1349.4355	
					1420.3209	
					1455.2227	
					1480.9763	
					1493.5251	
					1729.0606	
					3060.2161	
					3085.4164	
					3105.6263	

					3141.0649	
					3150.9073	
					3156.2763	
					3242.2448	
					3799.9636	
**R5**	6	-2.493657	-0.528619	-0.071501	893.8934i	205.03753
	6	-1.224778	0.121759	0.386378	52.0692	946.71886
	6	-0.214896	0.469831	-0.412711	98.3762	1070.26374
	6	1.044930	1.098435	0.056287	182.0642	
	1	-2.633816	-1.497566	0.416217	209.8490	
	1	-2.490180	-0.684460	-1.151339	238.1202	
	1	1.909269	0.330595	-0.008441	284.7076	
	1	1.002154	1.416266	1.098253	317.8996	
	1	-0.295093	0.266958	-1.479332	507.3953	
	1	-1.138785	0.328702	1.452438	673.8621	
	8	2.637847	-0.954806	0.014150	795.1382	
	1	1.850811	-1.490408	0.205851	888.9280	
	1	1.384646	1.914984	-0.583481	960.0099	
	1	-3.361379	0.084948	0.185924	997.7318	
					1012.4956	
					1073.8811	
					1106.0394	
					1171.9062	
					1309.2667	
					1332.9318	
					1343.9107	
					1375.2525	
					1421.7096	
					1467.9215	
					1487.5403	
					1500.0254	
					1572.2587	
					1760.3803	
					3051.8585	
					3081.2088	
					3113.1673	
					3127.5064	
					3137.6800	
					3144.5062	
					3159.7740	
					3795.5331	
**R6**	6	1.853526	-0.907085	0.004100	870.3029i	421.37249
	6	0.475044	-0.340436	-0.068610	104.8367	497.70249
	1	2.397096	-0.729970	-0.926612	131.5403	889.15797
	1	2.426291	-0.442140	0.810203	164.5792	
	1	1.811161	-1.984231	0.185317	195.1001	
	6	-0.694613	-0.948601	0.050708	226.3078	
	6	-2.035016	-0.279702	-0.024390	231.6981	
	1	-2.586998	-0.411894	0.910138	284.1463	
	1	-1.924467	0.787327	-0.221116	507.2852	
	1	-2.640845	-0.720119	-0.820365	588.2463	
	1	-0.689336	-2.029306	0.204068	832.2135	
	1	0.444646	0.823479	-0.273757	856.3115	

	8	0.367591	2.174590	-0.066513	908.5104	
	1	0.228086	2.165079	0.893383	986.2248	
					1057.6096	
					1070.7257	
					1095.9527	
					1154.0830	
					1219.2191	
					1309.5542	
					1385.8486	
					1411.2567	
					1426.9611	
					1473.4629	
					1490.8509	
					1494.5954	
					1501.6545	
					1770.6016	
					3041.9988	
					3042.9607	
					3102.3256	
					3107.9950	
					3116.2954	
					3120.3679	
					3143.5177	
					3809.2090	
**R7**	6	1.853526	-0.907085	0.004100	1164.0012i	212.18290
	6	0.475044	-0.340436	-0.068610	112.9768	736.07444
	1	2.397096	-0.729970	-0.926612	124.6843	916.65954
	1	2.426291	-0.442140	0.810203	162.4358	
	1	1.811161	-1.984231	0.185317	211.8433	
	6	-0.694613	-0.948601	0.050708	236.7768	
	6	-2.035016	-0.279702	-0.024390	352.9161	
	1	-2.586998	-0.411894	0.910138	398.7129	
	1	-1.924467	0.787327	-0.221116	441.0297	
	1	-2.640845	-0.720119	-0.820365	540.6105	
	1	-0.689336	-2.029306	0.204068	767.1563	
	1	0.444646	0.823479	-0.273757	821.8251	
	8	0.367591	2.174590	-0.066513	890.3660	
	1	0.228086	2.165079	0.893383	966.6047	
					968.1327	
					987.2593	
					1085.0058	
					1103.0553	
					1263.4557	
					1282.5162	
					1303.6520	
					1405.4233	
					1419.0491	
					1476.7249	
					1484.1430	
					1493.7580	
					1499.5173	
					1731.9015	
					3041.7745	

					3045.8704	
					3104.3775	
					3106.3619	
					3146.2574	
					3152.4445	
					3196.2628	
					3821.8602	
**R8**	6	-1.498107	-0.943794	-0.584919	911.5316i	305.34001
	6	-0.736609	-0.099758	0.114784	56.7184	651.67691
	6	0.372373	-0.585349	0.981850	95.9872	741.63388
	1	1.378604	-0.265980	0.496648	156.1454	
	1	0.404892	-1.668640	1.090787	223.2818	
	1	-1.353272	-2.016876	-0.524091	308.2742	
	8	2.373018	0.120839	-0.505113	394.8416	
	1	1.899124	-0.255995	-1.264692	430.8080	
	1	0.402569	-0.091266	1.955978	440.8268	
	6	-0.905762	1.392897	0.051167	677.4506	
	1	0.032679	1.862349	-0.258865	756.6916	
	1	-1.693820	1.680118	-0.644902	845.4157	
	1	-1.151835	1.790831	1.040150	909.4083	
	1	-2.294448	-0.585229	-1.227400	949.4472	
					974.0737	
					996.3751	
					1070.6899	
					1081.2102	
					1295.5075	
					1337.0035	
					1363.8413	
					1413.5963	
					1440.8166	
					1468.7783	
					1486.3823	
					1500.6428	
					1541.3659	
					1736.3487	
					3037.3211	
					3075.4130	
					3099.7314	
					3136.5019	
					3144.1005	
					3160.9468	
					3233.3425	
					3778.7423	
**R9**-*cis*	6	1.670139	-0.290635	-0.392703	1163.8539i	353.91659
	6	0.445948	-0.178326	0.523135	63.2464	1211.89074
	1	2.079550	-1.304474	-0.329339	73.3135	1467.30088
	1	1.349584	-0.145978	-1.428544	126.3211	
	6	-0.600499	-1.201490	0.190086	156.6582	
	1	0.767892	-0.319280	1.561889	242.5704	
	1	0.011618	0.822100	0.444876	255.3667	
	6	-1.825770	-0.939616	-0.226813	283.9109	
	1	-0.298779	-2.248031	0.271697	388.6183	
	1	-2.149289	0.211228	-0.369510	452.0372	

	1	-2.604400	-1.651329	-0.475455	555.8430	
	6	2.747290	0.727835	-0.030608	723.1332	
	1	3.097989	0.578132	0.993905	752.3680	
	1	3.609387	0.649440	-0.695472	846.6112	
	1	2.357367	1.746020	-0.104476	882.2162	
	8	-2.503149	1.451118	-0.128767	900.1334	
	1	-2.818377	1.346617	0.781989	949.1422	
					961.9009	
					1030.3398	
					1073.6002	
					1123.4560	
					1180.1429	
					1236.7780	
					1269.3711	
					1276.7180	
					1291.6489	
					1329.3796	
					1352.1042	
					1398.5680	
					1411.7552	
					1487.8255	
					1496.9028	
					1505.6986	
					1512.7929	
					1709.8155	
					3031.2820	
					3043.2982	
					3046.0181	
					3079.1897	
					3096.9378	
					3111.5741	
					3119.5282	
					3125.9404	
					3197.4219	
					3816.7407	
**R9-*trans***	6	1.761075	-0.587664	-0.080151	1222.4515i	150.99873
	6	0.864909	0.602137	0.281896	69.7815	1819.09632
	1	1.638906	-1.372061	0.673967	81.8378	1878.63786
	1	1.420048	-1.011476	-1.029497	109.5972	
	6	-0.576787	0.197171	0.426312	138.3181	
	1	1.210637	1.032990	1.228886	223.9193	
	1	0.957392	1.382585	-0.479421	253.3284	
	6	-1.563660	0.662549	-0.316789	264.3872	
	1	-0.799291	-0.538306	1.198558	373.5131	
	1	-1.492338	1.378540	-1.129660	408.7638	
	1	-2.681732	0.279560	-0.088261	537.6416	
	6	3.230716	-0.188579	-0.180317	752.5872	
	1	3.591856	0.211288	0.770730	781.1647	
	1	3.857146	-1.042250	-0.445328	862.8734	
	1	3.374249	0.582291	-0.941782	888.2956	
	8	-3.739385	-0.476498	0.003428	893.5919	
	1	-3.459302	-1.204862	-0.571317	949.9031	
					977.2486	

					1034.2002	
					1072.1530	
					1122.4754	
					1175.8780	
					1238.7922	
					1271.4464	
					1287.0709	
					1301.4329	
					1331.0790	
					1343.2513	
					1392.5776	
					1420.5080	
					1488.0219	
					1501.7292	
					1509.2979	
					1515.0778	
					1711.1792	
					3045.4591	
					3059.0409	
					3062.0245	
					3087.1542	
					3106.8606	
					3134.2570	
					3137.9026	
					3141.7431	
					3182.4733	
					3827.5477	
**R10**	6	-1.286187	0.288278	-0.365918	926.6365i	427.36804
	6	-0.432513	-0.657701	0.486068	51.7693	1022.10328
	1	-0.950802	1.315324	-0.192387	75.1897	1353.91256
	1	-1.109011	0.069206	-1.423605	101.4397	
	6	1.024430	-0.534826	0.185573	150.4137	
	1	-0.604884	-0.436365	1.545296	185.8666	
	1	-0.739588	-1.697158	0.317458	238.1694	
	6	1.874132	-1.437163	-0.273370	270.0819	
	1	1.455889	0.550924	0.372747	376.8916	
	1	1.556913	-2.462812	-0.447419	422.6534	
	1	2.910702	-1.192847	-0.474351	588.2372	
	6	-2.772824	0.162593	-0.045966	725.0770	
	1	-2.966300	0.401789	1.002805	749.3895	
	1	-3.369978	0.839223	-0.659749	862.7774	
	1	-3.127506	-0.855368	-0.226952	872.0144	
	8	1.846437	1.836600	0.136777	903.1006	
	1	1.730840	1.848189	-0.826382	950.9974	
					960.6599	
					1030.6882	
					1073.6848	
					1122.0753	
					1172.5460	
					1213.7932	
					1259.4877	
					1290.7878	
					1325.5045	

					1381.6184	
					1417.1217	
					1420.8132	
					1434.0563	
					1474.9668	
					1501.3550	
					1505.5558	
					1513.7135	
					1738.3191	
					3027.9864	
					3047.3302	
					3051.8236	
					3072.6071	
					3094.7685	
					3121.7445	
					3123.5960	
					3135.0440	
					3228.8417	
					3812.5453	
**R11**	6	-1.286187	0.288278	-0.365918	634.6449i	513.20123
	6	-0.432513	-0.657701	0.486068	39.5656	894.91983
	1	-0.950802	1.315324	-0.192387	78.3647	1291.41560
	1	-1.109011	0.069206	-1.423605	88.5673	
	6	1.024430	-0.534826	0.185573	119.1372	
	1	-0.604884	-0.436365	1.545296	155.4970	
	1	-0.739588	-1.697158	0.317458	224.9740	
	6	1.874132	-1.437163	-0.273370	270.2832	
	1	1.455889	0.550924	0.372747	395.9494	
	1	1.556913	-2.462812	-0.447419	435.5977	
	1	2.910702	-1.192847	-0.474351	583.4771	
	6	-2.772824	0.162593	-0.045966	668.9685	
	1	-2.966300	0.401789	1.002805	742.5152	
	1	-3.369978	0.839223	-0.659749	858.8333	
	1	-3.127506	-0.855368	-0.226952	902.3743	
	8	1.846437	1.836600	0.136777	971.8463	
	1	1.730840	1.848189	-0.826382	975.4398	
					1029.6665	
					1052.1124	
					1086.9788	
					1117.1495	
					1198.6654	
					1265.0759	
					1288.2329	
					1308.2880	
					1328.9741	
					1359.7013	
					1386.9201	
					1418.2439	
					1462.7326	
					1486.6323	
					1507.8305	
					1510.4816	
					1651.8050	

					1745.1130	
					3046.9702	
					3055.7485	
					3074.8944	
					3091.9656	
					3128.2293	
					3138.9286	
					3144.0907	
					3148.2918	
					3235.1228	
					3793.5956	
**R12**	6	-2.458621	-0.156175	-0.263522	769.0202i	442.61474
	6	-1.425829	-0.696851	0.376528	71.0499	858.89693
	6	-0.090027	-0.979980	-0.244991	101.1894	1189.98353
	6	1.038722	-0.199452	0.420004	110.0588	
	6	2.401043	-0.427724	-0.207018	137.2390	
	1	1.049593	-0.360473	1.502232	212.5807	
	1	0.791126	0.931894	0.298585	235.3064	
	1	0.131247	-2.052022	-0.158123	292.2892	
	1	2.689532	-1.480548	-0.121601	388.3250	
	1	2.386270	-0.167744	-1.267892	444.8792	
	1	-2.396720	0.095714	-1.318389	649.8280	
	1	-3.399538	0.037003	0.238068	676.4292	
	1	-1.525245	-0.940537	1.433257	743.5522	
	1	-0.118162	-0.742827	-1.313269	889.6762	
	8	0.149159	2.176876	-0.017323	910.5393	
	1	-0.762540	1.851971	0.079460	967.6596	
	1	3.169442	0.173650	0.280253	972.9993	
					1021.1663	
					1042.2258	
					1082.8007	
					1093.5874	
					1172.8440	
					1205.8297	
					1265.5383	
					1290.2508	
					1327.0017	
					1354.2541	
					1374.8664	
					1410.8601	
					1453.8391	
					1482.0379	
					1491.0880	
					1493.4150	
					1530.8579	
					1724.7710	
					3024.1996	
					3039.7282	
					3072.3915	
					3091.4288	
					3112.5372	
					3134.7413	
					3139.0675	

					3142.8128	
					3233.6609	
					3762.7172	
**R13**	6	1.637067	1.090381	-0.241489	783.5928i	286.97187
	6	0.254686	0.959598	0.365479	46.1456	1353.28013
	1	2.233294	0.115181	0.001860	67.2623	1524.45712
	6	-0.532168	-0.216471	-0.223478	101.2297	
	1	0.338246	0.836893	1.449809	132.4995	
	1	-0.305466	1.887078	0.196896	151.5554	
	6	-1.891105	-0.358201	0.394673	232.9920	
	1	-0.632761	-0.087814	-1.306698	316.2666	
	1	0.046130	-1.131872	-0.052533	391.1072	
	6	-3.039528	-0.256862	-0.264701	442.8893	
	1	-3.994570	-0.360447	0.236409	653.4876	
	1	-3.059303	-0.070438	-1.334156	724.7052	
	1	-1.909245	-0.545580	1.467134	788.1901	
	8	2.769755	-1.194950	0.135787	865.3034	
	1	2.693176	-1.466205	-0.792855	898.9394	
	1	2.239294	1.888525	0.194440	966.4680	
	1	1.619449	1.183611	-1.329511	970.7643	
					1030.8980	
					1045.4105	
					1062.9782	
					1090.0649	
					1176.7271	
					1207.3602	
					1263.0034	
					1295.2674	
					1312.0620	
					1333.3515	
					1341.6116	
					1387.4335	
					1450.5787	
					1455.9591	
					1477.1816	
					1486.7230	
					1496.3631	
					1731.6536	
					3028.9041	
					3038.5112	
					3072.0438	
					3078.6897	
					3094.8100	
					3138.8744	
					3146.7515	
					3147.9099	
					3236.3794	
					3792.7751	
**R14**	6	2.016977	-0.368080	0.274280	892.7173i	345.72087
	6	0.661899	-0.603135	-0.322562	37.8727	1335.29528
	1	1.951252	-0.430877	1.363919	70.2966	1578.36807
	1	2.696605	-1.163314	-0.051165	112.6467	
	6	-0.459432	-0.796301	0.374407	158.1500	

	6	-1.799141	-0.995514	-0.231785	181.5877	
	1	-2.376055	-1.788899	0.247119	222.5893	
	1	-2.446279	-0.051885	-0.056759	286.6604	
	1	-1.762661	-1.142578	-1.311290	332.8083	
	6	2.598863	0.986686	-0.142985	415.7458	
	1	1.961747	1.803077	0.203759	491.9342	
	1	3.597163	1.130856	0.274411	697.5346	
	1	2.674958	1.059317	-1.230783	780.2261	
	1	-0.408565	-0.790584	1.461875	830.2657	
	1	0.608157	-0.605326	-1.411984	894.7660	
	8	-2.834267	1.365796	0.104305	956.0168	
	1	-1.937186	1.711910	-0.031673	980.2746	
					1011.8153	
					1037.5038	
					1095.4496	
					1117.0847	
					1183.1381	
					1270.6125	
					1309.6013	
					1327.0395	
					1337.4670	
					1361.1464	
					1383.5107	
					1411.0503	
					1471.2576	
					1486.6955	
					1504.2139	
					1510.3554	
					1571.2529	
					1754.9738	
					3042.1148	
					3051.9852	
					3083.9333	
					3091.6366	
					3121.6711	
					3127.7130	
					3138.5433	
					3139.9262	
					3159.7553	
					3799.0612	
**R15**	6	-1.477485	0.260075	-0.468138	864.2298i	465.85626
	6	-0.334967	-0.709243	-0.362728	61.5215	974.22264
	1	-1.119311	1.263774	-0.227281	102.0384	1333.29369
	1	-1.836904	0.279609	-1.502781	129.9965	
	6	0.898780	-0.412620	0.014506	174.2459	
	6	2.091682	-1.298434	0.151916	191.8182	
	1	1.831242	-2.327756	-0.108311	206.9709	
	1	2.901858	-0.969475	-0.503386	226.6700	
	6	-2.634289	-0.126456	0.458168	299.6947	
	1	-2.308438	-0.120323	1.500170	413.5421	
	1	-3.467096	0.571574	0.355083	495.4631	
	1	-3.003438	-1.128757	0.226515	589.1856	
	1	1.106627	0.716910	0.296101	783.3018	

	1	-0.562073	-1.754504	-0.587966	865.2013	
	1	2.473346	-1.282204	1.175189	877.5294	
	8	1.408815	2.046759	0.175793	896.2746	
	1	1.451339	2.097149	-0.792016	967.1807	
					1034.3456	
					1056.1130	
					1095.2775	
					1109.4833	
					1169.5401	
					1214.0429	
					1286.0453	
					1308.7517	
					1353.8369	
					1397.8442	
					1408.6483	
					1420.1250	
					1473.0202	
					1488.2709	
					1493.6020	
					1504.5682	
					1509.4140	
					1763.1520	
					3045.1920	
					3045.5406	
					3048.8258	
					3097.1019	
					3112.9780	
					3114.9947	
					3125.2990	
					3128.5657	
					3136.4493	
					3808.5432	
**R16**	6	-1.407783	-0.595495	-0.562456	803.4283i	486.83278
	6	-0.004772	-0.242098	-0.188869	52.4103	892.98306
	1	-1.683385	-0.059079	-1.476711	83.8717	1254.95842
	1	-1.448347	-1.665359	-0.794904	141.7869	
	6	1.075018	-1.004075	-0.109078	167.0584	
	6	2.443424	-0.529549	0.281103	186.4525	
	1	3.162525	-0.712470	-0.521886	214.1071	
	1	2.432100	0.537451	0.507345	227.8755	
	6	-2.399432	-0.244050	0.549306	295.3951	
	1	-2.348488	0.822427	0.779600	405.7633	
	1	-3.422281	-0.482404	0.252070	490.8856	
	1	-2.166145	-0.797845	1.460698	635.6986	
	1	0.125788	0.892311	0.103871	777.1790	
	1	2.801129	-1.069057	1.161922	844.2904	
	1	0.962951	-2.066936	-0.331002	868.9372	
	8	0.342164	2.251901	-0.003143	908.4691	
	1	0.608111	2.277362	-0.935902	967.5711	
					1036.2563	
					1070.2447	
					1094.6447	
					1108.3467	

					1170.5087	
					1221.1391	
					1264.1375	
					1315.5306	
					1347.6891	
					1408.6498	
					1410.4903	
					1437.5161	
					1472.9110	
					1492.6072	
					1500.2488	
					1503.1895	
					1511.7703	
					1765.8423	
					3040.4831	
					3046.3741	
					3055.1479	
					3081.1695	
					3105.6576	
					3107.5182	
					3133.6690	
					3137.6366	
					3144.8654	
					3813.0799	
**R17**	6	1.554339	-0.037609	0.427272	637.6091i	453.01319
	6	0.411306	-0.218176	-0.234120	53.2067	1095.02691
	6	-0.915480	-0.438197	0.406672	74.1245	1434.95096
	1	-1.544772	0.509175	0.251778	109.5418	
	1	-0.816397	-0.534632	1.491122	175.9796	
	1	1.533633	-0.060221	1.516091	200.8254	
	8	-1.977813	1.926576	-0.097978	204.7892	
	1	-1.074461	2.282129	-0.068784	229.0361	
	1	0.422011	-0.202326	-1.324352	290.6097	
	6	-1.717822	-1.582461	-0.200462	415.4030	
	1	-1.192874	-2.531232	-0.059852	483.5933	
	1	-2.702624	-1.662456	0.261702	638.2212	
	1	-1.857355	-1.428325	-1.272744	752.6376	
	6	2.889331	0.188433	-0.214212	836.3857	
	1	3.320404	1.140771	0.107350	895.0762	
	1	3.597131	-0.594087	0.072829	967.5346	
	1	2.807768	0.196663	-1.302222	1002.3618	
					1031.2473	
					1070.1197	
					1080.2029	
					1108.0513	
					1182.1897	
					1235.8907	
					1284.1167	
					1331.1189	
					1357.0528	
					1387.3372	
					1409.3271	
					1417.6549	

					1482.8002	
					1495.1638	
					1498.3512	
					1500.0821	
					1664.0612	
					1775.4374	
					3040.9704	
					3047.0393	
					3091.2613	
					3099.7187	
					3119.5793	
					3122.8464	
					3126.0158	
					3139.3623	
					3141.4572	
					3787.1298	
**R18**	6	-2.056893	-0.504969	-0.305508	757.5037i	117.74480
	6	-0.863956	-1.177083	0.356585	49.2816	1925.66786
	1	-1.965891	0.662383	-0.142893	55.3488	1950.66197
	1	-2.085197	-0.642386	-1.387730	89.9898	
	1	-3.013680	-0.772442	0.144403	159.7661	
	6	0.439379	-0.747840	-0.254029	177.8787	
	1	-0.860486	-0.957390	1.427664	214.8662	
	1	-0.971712	-2.264399	0.253492	279.7454	
	6	1.416079	-0.124354	0.401729	331.9377	
	1	0.567238	-0.956494	-1.316702	406.0459	
	1	1.279976	0.079271	1.463649	498.6920	
	8	-1.439511	1.917306	0.086087	731.2567	
	1	-0.505859	1.666578	-0.019050	777.1280	
	6	2.715144	0.313843	-0.205698	813.1309	
	1	3.558123	-0.181505	0.283792	917.8283	
	1	2.751044	0.080197	-1.270976	993.0630	
	1	2.864021	1.390159	-0.082818	1003.3091	
					1030.6419	
					1049.0450	
					1075.8029	
					1104.2150	
					1174.8142	
					1206.3349	
					1278.1905	
					1301.6011	
					1324.2105	
					1339.4368	
					1367.4971	
					1420.1253	
					1452.6896	
					1483.0554	
					1490.0557	
					1496.2410	
					1501.1932	
					1754.6671	
					3045.7173	
					3053.9767	

					3087.1350	
					3106.8178	
					3110.5653	
					3121.6791	
					3125.8165	
					3139.1144	
					3159.4910	
					3810.4955	
**R19-*cis***	6	-1.208906	1.723647	-0.385694	1182.3625i	470.72099
	6	-0.486029	0.820465	0.619400	40.0733	837.86479
	1	-1.230512	2.753871	-0.026461	117.6161	1203.14000
	1	-0.696463	1.711540	-1.350753	126.0270	
	1	-2.241644	1.407512	-0.546177	187.9670	
	6	-0.358123	-0.602912	0.133374	211.6193	
	1	0.508997	1.216326	0.828816	239.7675	
	1	-1.043149	0.814256	1.563279	272.4574	
	6	0.816224	-1.171649	-0.093283	363.3876	
	1	1.820447	-0.542225	0.126984	411.3194	
	1	0.998639	-2.175871	-0.460726	505.6803	
	8	2.800240	0.314248	0.005243	588.7694	
	1	2.701296	0.526546	-0.935212	757.6528	
	6	-1.643524	-1.365282	-0.080673	786.1702	
	1	-2.224117	-0.914457	-0.890045	799.5128	
	1	-1.454657	-2.408423	-0.333594	883.0267	
	1	-2.258604	-1.328668	0.823202	941.6592	
					993.4888	
					1016.4842	
					1035.6967	
					1095.6740	
					1120.5556	
					1243.7750	
					1285.0845	
					1296.1080	
					1315.9273	
					1369.7727	
					1404.6923	
					1408.3615	
					1481.5206	
					1488.2277	
					1497.6142	
					1502.4566	
					1512.7238	
					1724.7907	
					3043.3319	
					3048.0451	
					3050.2459	
					3107.9303	
					3114.5704	
					3126.5085	
					3132.5135	
					3148.2146	
					3187.4626	
					3823.5690	

**R19-*trans***	6	-0.495120	-0.799147	-0.146256	1142.9883i	331.38559
	6	0.152404	0.350993	-0.038874	82.0552	1072.37010
	6	-0.558678	1.677673	-0.016884	100.9074	1361.01606
	6	1.661921	0.429790	0.049757	112.9628	
	1	-1.691529	-0.771781	-0.270847	143.7280	
	1	-0.202357	2.308320	-0.836571	237.6180	
	1	-1.636882	1.552737	-0.109439	247.7571	
	1	-0.339347	2.207791	0.914729	269.9723	
	1	2.006349	1.057397	-0.780645	386.1748	
	1	-0.078906	-1.799393	-0.153104	428.9504	
	8	-2.972569	-0.687822	-0.009950	467.1989	
	1	-2.946167	-0.869676	0.941684	549.9006	
	6	2.402155	-0.901136	0.037301	740.4160	
	1	2.201656	-1.454306	-0.882772	791.9164	
	1	3.479044	-0.739210	0.103788	811.6299	
	1	2.103844	-1.526456	0.881735	883.6277	
	1	1.908763	0.988109	0.960771	956.4683	
					1006.1708	
					1029.0381	
					1032.7926	
					1112.1541	
					1123.0743	
					1236.9820	
					1271.4319	
					1299.5154	
					1301.0818	
					1382.6839	
					1418.0820	
					1423.1934	
					1471.4710	
					1486.7795	
					1493.1416	
					1505.1175	
					1511.7685	
					1722.5067	
					3036.8032	
					3042.1111	
					3056.0711	
					3067.4300	
					3099.9821	
					3130.1887	
					3133.8970	
					3154.1207	
					3203.5071	
					3818.3727	
**R20**	6	-0.558580	1.529169	0.676563	929.0200i	440.67554
	6	-0.131903	0.599221	-0.181466	58.3439	928.72672
	6	1.204603	0.704506	-0.832235	65.4629	1114.23900
	1	1.895694	-0.110887	-0.376868	117.2832	
	1	1.699801	1.660938	-0.669357	228.6819	
	1	0.045376	2.402158	0.898821	280.4981	
	8	2.449529	-1.084256	0.565979	291.9956	
	1	2.043310	-0.691567	1.356017	311.4124	

	1	1.186683	0.448129	-1.894293	411.8596	
	6	-0.912410	-0.642420	-0.536336	426.8327	
	1	-0.262837	-1.504537	-0.342511	493.8255	
	1	-1.073671	-0.637810	-1.621023	662.0630	
	1	-1.519307	1.457150	1.171739	761.4259	
	6	-2.244002	-0.816436	0.182475	802.4704	
	1	-2.923869	0.009938	-0.036937	807.5429	
	1	-2.728939	-1.741516	-0.132479	907.0403	
	1	-2.104716	-0.862190	1.265063	951.3798	
					976.6320	
					1013.3250	
					1036.8392	
					1105.9948	
					1114.2571	
					1261.7395	
					1297.8242	
					1334.2459	
					1348.9555	
					1399.8120	
					1418.2894	
					1440.1194	
					1474.4138	
					1485.0003	
					1506.0397	
					1517.7176	
					1543.3003	
					1731.5145	
					3033.7449	
					3050.2999	
					3064.0043	
					3088.0056	
					3123.9067	
					3129.6787	
					3154.1018	
					3166.0270	
					3240.0178	
					3799.0369	
**R21**	6	-1.217084	1.060389	-0.925639	853.6418i	567.40719
	6	-0.831228	0.217336	0.033952	56.0501	695.58624
	6	0.528145	0.261382	0.661266	90.1948	994.61593
	1	1.044676	-0.729657	0.364382	129.9379	
	1	0.442477	0.155408	1.748630	185.1285	
	1	-0.574599	1.853070	-1.289902	235.4383	
	8	1.478236	-1.842651	-0.539304	248.6055	
	1	1.019103	-1.498748	-1.323635	292.3351	
	6	-1.726801	-0.877016	0.549306	401.5173	
	1	-1.940001	-0.725173	1.611641	409.7398	
	1	-1.230268	-1.847533	0.456529	503.6070	
	1	-2.205546	0.986883	-1.365114	637.5356	
	1	-2.672554	-0.910456	0.008244	740.3892	
	6	1.450835	1.401135	0.275708	801.4216	
	1	1.014722	2.365083	0.552109	817.0301	
	1	2.410802	1.302153	0.783386	943.1068	

	1	1.642103	1.410822	-0.799392	970.0695	
					1008.4977	
					1019.1305	
					1050.6770	
					1088.8067	
					1112.2777	
					1219.7907	
					1265.8308	
					1353.6960	
					1392.1056	
					1417.7205	
					1419.9648	
					1453.5529	
					1486.7573	
					1495.3837	
					1497.3388	
					1509.3512	
					1651.8489	
					1745.1295	
					3044.5931	
					3046.7529	
					3068.9678	
					3106.6347	
					3117.8709	
					3139.4030	
					3141.1169	
					3153.1990	
					3238.1597	
					3772.3837	
**R22**	6	-1.364302	-1.065742	0.084038	1000.4636i	502.38620
	6	-0.079760	-0.895028	0.881257	52.4166	769.11795
	1	-1.786450	0.006028	-0.194897	108.4170	886.42173
	1	-1.225355	-1.568112	-0.873622	146.8136	
	1	-2.163444	-1.540821	0.653176	187.7445	
	6	0.938278	-0.027332	0.180804	268.0842	
	1	-0.304188	-0.474594	1.864169	315.0643	
	1	0.361770	-1.886882	1.048447	363.4588	
	6	1.427417	1.071781	0.755233	407.4481	
	1	1.117154	1.375313	1.749542	436.7105	
	8	-1.892022	1.348939	-0.472969	533.4063	
	1	-0.980303	1.598908	-0.241955	694.3198	
	1	2.163420	1.690721	0.253767	727.6748	
	6	1.347716	-0.460165	-1.200882	790.0820	
	1	1.628240	-1.517991	-1.206082	880.7968	
	1	0.518694	-0.341759	-1.905106	939.5401	
	1	2.190539	0.126594	-1.566377	948.3570	
					981.8893	
					1002.1136	
					1047.6605	
					1066.8211	
					1105.2428	
					1197.5136	
					1256.3765	

					1311.3395	
					1332.1277	
					1362.4485	
					1414.6767	
					1449.6592	
					1463.1133	
					1471.5984	
					1479.6152	
					1490.8118	
					1501.9490	
					1730.5891	
					3034.9855	
					3044.7058	
					3094.4498	
					3101.8078	
					3106.2608	
					3138.7362	
					3141.7931	
					3169.6324	
					3230.3074	
					3763.7564	
**R23**	6	0.350764	0.492933	-0.112572	838.5251i	489.45835
	6	-0.759132	-0.232605	-0.026344	89.6197	763.66373
	1	1.346269	-0.116639	-0.279958	100.6833	1208.11226
	6	-0.733818	-1.737538	-0.093724	104.5836	
	6	-2.136202	0.365322	0.124410	116.2511	
	6	0.595506	1.964754	-0.047353	158.3773	
	1	-1.368610	-2.089452	-0.912645	192.6513	
	1	0.276410	-2.116203	-0.246464	246.7076	
	1	-1.138309	-2.165350	0.829159	287.2988	
	1	-2.615084	-0.028617	1.026027	380.5144	
	1	-2.128132	1.451935	0.184876	406.8425	
	1	-2.762073	0.073583	-0.724170	517.6904	
	1	-0.312529	2.519280	0.195205	580.7806	
	1	1.345664	2.193097	0.713870	760.8098	
	1	0.981539	2.334278	-1.000671	877.7102	
	8	2.509953	-0.790085	0.020831	968.3236	
	1	2.392518	-0.852427	0.981614	977.7878	
					985.0839	
					1050.0748	
					1069.8103	
					1101.0573	
					1138.2831	
					1218.7462	
					1290.5557	
					1401.9945	
					1407.1046	
					1415.5950	
					1458.4506	
					1478.8986	
					1481.2059	
					1484.2585	
					1497.3316	

					1498.8960	
					1502.8200	
					1771.4930	
					3040.6885	
					3046.7009	
					3054.4488	
					3097.2532	
					3104.1321	
					3117.8416	
					3136.9044	
					3150.8509	
					3158.7918	
					3807.5764	
**R24**	6	1.189024	-0.662483	-0.245575	859.9117i	388.33530
	6	0.175373	0.201166	-0.391831	46.9349	1077.45264
	6	-1.050682	-0.198496	-1.139205	89.0708	1135.93342
	1	-1.927915	-0.290493	-0.389043	107.5298	
	1	-0.964193	-1.161178	-1.642211	111.9327	
	1	1.075869	-1.640237	-0.711191	192.3402	
	8	-2.652810	-0.506915	0.888477	221.0566	
	1	-1.919924	-0.978487	1.317357	305.7892	
	1	-1.398618	0.571930	-1.832348	329.3357	
	6	0.126587	1.592762	0.178808	400.2017	
	1	-0.786463	1.712342	0.770032	443.3195	
	1	0.980789	1.832257	0.808736	524.2067	
	1	0.080936	2.328742	-0.629879	714.7804	
	6	2.487849	-0.448316	0.470810	793.4231	
	1	2.567287	0.538026	0.924853	838.0530	
	1	2.618840	-1.196344	1.258212	928.4640	
	1	3.326971	-0.569038	-0.220381	967.7108	
					990.1517	
					1062.4261	
					1068.8975	
					1074.2891	
					1143.6599	
					1241.3508	
					1346.7413	
					1367.0213	
					1382.2044	
					1424.1946	
					1432.2406	
					1463.0673	
					1485.7082	
					1491.4796	
					1493.6712	
					1504.0050	
					1595.9529	
					1769.8975	
					3040.5636	
					3050.1974	
					3074.8372	
					3094.3749	
					3102.3076	

					3143.7103	
					3154.4041	
					3158.3302	
					3171.0247	
					3802.7987	
**R25-*cis***	6	-1.343635	0.260040	-0.355918	930.7670i	531.69778
	6	-0.163979	0.617757	0.168787	27.9537	816.90636
	6	0.605254	-0.212966	1.137377	77.7585	1094.13760
	1	1.461115	-0.746039	0.565036	95.4855	
	8	2.217348	-1.320251	-0.569325	181.5305	
	1	1.476015	-1.255152	-1.193840	235.0282	
	1	1.135377	0.394895	1.873743	244.1788	
	6	0.517204	1.898821	-0.236042	293.6596	
	1	1.523160	1.688351	-0.612972	318.1588	
	1	0.630877	2.563546	0.625645	392.4317	
	1	-1.799282	0.949655	-1.064046	423.6241	
	6	-2.131753	-0.985622	-0.080788	523.3443	
	1	-3.092471	-0.739220	0.381061	709.6853	
	1	-2.357392	-1.506410	-1.015580	797.5968	
	1	-1.611406	-1.684638	0.572346	838.0437	
	1	-0.041723	2.427084	-1.009084	911.8267	
	1	0.038402	-0.998241	1.631789	968.4630	
					983.6683	
					1055.5859	
					1070.3426	
					1075.0758	
					1137.1085	
					1241.8923	
					1326.9770	
					1344.4702	
					1392.3446	
					1414.0653	
					1421.1295	
					1468.9616	
					1484.1527	
					1485.4227	
					1501.1225	
					1502.4409	
					1590.0371	
					1768.7114	
					3035.1301	
					3042.2853	
					3085.6902	
					3092.7090	
					3094.2276	
					3138.3433	
					3141.3274	
					3155.5407	
					3179.9589	
					3788.6723	
**R25-*trans***	6	-2.488468	-0.446756	0.471839	858.8932i	388.10341
	6	-1.189669	-0.662988	-0.243970	46.3865	1078.08180
	1	-2.567053	0.540102	0.924901	89.0131	1136.46153

	1	-3.327508	-0.567277	-0.219497	107.7320	
	6	-0.175618	0.199888	-0.391944	112.1212	
	6	1.050214	-0.201692	-1.138687	185.2474	
	1	1.398149	0.567202	-1.833541	220.9753	
	1	1.927678	-0.292381	-0.388751	305.7599	
	1	0.963324	-1.165391	-1.639692	329.0533	
	1	-1.076991	-1.641684	-0.707704	400.1058	
	8	2.654407	-0.504360	0.888694	443.1903	
	1	1.921635	-0.973184	1.320786	524.1566	
	1	-2.620445	-1.193922	1.259885	714.1492	
	6	-0.126387	1.592739	0.175642	793.3623	
	1	-0.082499	2.326979	-0.634727	838.2074	
	1	-0.979614	1.833229	0.806517	928.6680	
	1	0.787634	1.714063	0.764991	967.7827	
					990.0958	
					1062.5108	
					1068.9103	
					1074.2714	
					1143.6132	
					1241.3564	
					1346.8748	
					1367.2906	
					1382.2679	
					1424.1383	
					1432.2015	
					1463.1036	
					1485.7005	
					1491.5020	
					1493.6642	
					1504.0038	
					1595.5732	
					1769.8389	
					3040.4664	
					3050.1430	
					3075.0815	
					3094.2717	
					3102.2657	
					3143.8035	
					3154.5539	
					3158.4387	
					3170.9216	
					3803.6679	
**R26-*cis***	6	-1.344256	1.012729	-0.559652	1183.9132i	417.31221
	6	-0.034465	0.855005	-0.602999	28.9959	1026.96480
	6	0.756246	-0.172165	0.161905	98.2207	1082.89974
	6	1.451466	-1.129257	-0.811958	160.0385	
	6	1.781066	0.520929	1.064752	196.9936	
	1	-1.983204	0.298655	0.170139	201.4534	
	1	0.549562	1.526156	-1.239073	240.9967	
	1	0.056976	-0.739211	0.783059	322.1717	
	1	2.031316	-1.876963	-0.265757	342.3659	
	1	0.726411	-1.648273	-1.442106	356.9661	
	1	2.138465	-0.581962	-1.464959	509.2135	

	1	2.360361	-0.218142	1.623158	577.1917	
	1	2.479969	1.114832	0.467574	736.9852	
	1	1.291679	1.186528	1.777995	804.2722	
	1	-1.942163	1.728494	-1.111701	868.9479	
	8	-2.590131	-0.752954	0.666057	929.6950	
	1	-2.648668	-1.289932	-0.139081	945.2369	
					952.1156	
					972.7923	
					980.4161	
					1136.1386	
					1178.2203	
					1206.3929	
					1243.6825	
					1286.9225	
					1303.6335	
					1354.4922	
					1386.6910	
					1397.5514	
					1417.2228	
					1488.1978	
					1491.1485	
					1504.4127	
					1512.7525	
					1710.5991	
					3034.7824	
					3039.2868	
					3075.0690	
					3103.7316	
					3108.9527	
					3117.6199	
					3122.3990	
					3127.9932	
					3198.9156	
					3816.5564	
**R26-*trans***	6	1.253549	-0.208002	0.706709	1227.5304i	273.69668
	6	0.227090	-0.078810	-0.112829	74.5798	1386.96428
	6	-1.216982	-0.007819	0.317742	78.8959	1510.51684
	6	-1.825724	1.330517	-0.112995	97.3243	
	6	-2.000572	-1.179960	-0.280377	208.9462	
	1	2.355333	-0.297354	0.233002	215.8246	
	1	1.226010	-0.253605	1.790949	239.3703	
	1	0.406831	-0.030384	-1.187578	288.6034	
	1	-1.252907	-0.077955	1.409773	350.2313	
	1	-2.874985	1.388383	0.186738	355.5203	
	1	-1.291245	2.170605	0.334405	460.6436	
	1	-1.780484	1.438184	-1.200935	564.4058	
	1	-3.049216	-1.135478	0.023660	795.6101	
	1	-1.965324	-1.144646	-1.373332	815.8825	
	1	-1.586499	-2.137116	0.041592	861.0937	
	8	3.473906	-0.002483	-0.373792	933.9049	
	1	3.397071	0.963672	-0.377440	943.1402	
					965.9957	
					978.9375	

					989.5905	
					1135.0167	
					1180.6420	
					1198.6989	
					1239.9907	
					1293.5424	
					1305.9314	
					1341.3059	
					1370.6973	
					1398.1513	
					1418.2733	
					1490.7323	
					1491.4560	
					1504.6374	
					1513.5553	
					1709.7890	
					3041.9365	
					3043.7654	
					3061.3283	
					3114.1642	
					3118.7677	
					3124.3911	
					3129.6285	
					3132.9214	
					3184.1823	
					3820.2091	
**R27**	6	-1.326048	1.683864	0.098176	842.6320i	569.34534
	6	-0.580488	0.602350	-0.046926	51.5313	756.12682
	6	0.899198	0.422098	0.090192	86.2291	936.48858
	6	1.216187	-0.508611	1.265479	142.7408	
	6	1.487278	-0.130115	-1.212123	200.6566	
	1	-1.128952	-0.399837	-0.341759	224.9781	
	1	1.328367	1.412098	0.291058	259.2584	
	1	2.296308	-0.631171	1.375102	290.4508	
	1	0.815498	-0.114502	2.201646	345.1553	
	1	0.779202	-1.495396	1.088410	354.5101	
	1	2.567650	-0.262863	-1.118761	510.0325	
	1	1.040679	-1.102336	-1.438604	651.4312	
	1	1.290020	0.540560	-2.050271	724.4526	
	1	-2.402352	1.656453	-0.024661	801.8255	
	1	-0.872325	2.643885	0.333354	884.3081	
	8	-1.733954	-1.633988	-0.267874	938.2424	
	1	-2.019229	-1.592502	0.658688	944.8049	
					952.6151	
					977.1198	
					997.7775	
					1133.8927	
					1185.8829	
					1202.8124	
					1237.6586	
					1316.8350	
					1328.9721	
					1391.0419	

					1399.7978	
					1423.0736	
					1433.7138	
					1490.1026	
					1496.2481	
					1507.8807	
					1518.3301	
					1724.4310	
					3037.6876	
					3042.9480	
					3049.8605	
					3119.6921	
					3123.7751	
					3124.7251	
					3130.0180	
					3139.9511	
					3225.1473	
					3802.3626	
**R28**	6	-2.012030	-0.868701	-0.074556	558.9104i	602.05967
	6	-0.924137	-0.386762	-0.670375	55.7488	727.86306
	6	0.167846	0.402869	-0.015688	64.6514	915.08036
	1	1.101460	-0.262853	-0.072251	125.1204	
	1	-2.207625	-0.711773	0.980370	201.5918	
	8	1.972885	-1.522470	-0.026178	213.9488	
	1	1.218280	-2.132335	0.025534	248.2696	
	1	-2.752285	-1.429178	-0.632794	302.0153	
	1	-0.783796	-0.568424	-1.734992	363.8122	
	6	0.517087	1.650202	-0.825146	371.3222	
	1	-0.324173	2.350455	-0.806841	530.3530	
	1	1.390862	2.151805	-0.405338	595.2058	
	1	0.730438	1.401053	-1.866557	705.7273	
	6	-0.053965	0.705147	1.457138	796.8729	
	1	0.811191	1.227252	1.869021	919.8152	
	1	-0.932875	1.344988	1.587916	946.4390	
	1	-0.203363	-0.207759	2.037118	970.6971	
					978.2955	
					1029.7994	
					1051.5379	
					1076.0458	
					1153.5844	
					1189.4606	
					1232.5727	
					1322.8386	
					1338.5829	
					1399.0380	
					1414.7172	
					1453.3873	
					1484.7256	
					1489.5241	
					1501.4005	
					1511.3035	
					1699.1376	
					1786.9513	

					3033.1483	
					3040.9735	
					3108.5730	
					3115.7932	
					3127.5190	
					3138.0648	
					3142.6970	
					3158.4725	
					3242.9030	
					3792.8935	
**R29**	6	0.098881	-1.429299	0.243103	997.8284i	498.77916
	6	-0.662177	-0.221746	-0.285466	73.7659	732.51957
	1	1.258034	-1.266528	0.069356	100.4420	1131.28003
	1	-0.018610	-1.570583	1.320236	134.3225	
	1	-0.133273	-2.350397	-0.294646	213.7220	
	6	-0.244802	1.024879	0.444929	292.4249	
	1	-0.423452	-0.096411	-1.346956	295.2290	
	6	0.336343	2.079502	-0.118627	315.6498	
	1	-0.446480	1.032708	1.516555	334.0569	
	1	0.544306	2.103112	-1.184490	382.5628	
	8	2.490601	-0.697372	-0.143428	505.1999	
	1	2.225031	0.219076	0.043927	693.6545	
	1	0.611157	2.953669	0.459992	757.1456	
	6	-2.174907	-0.443929	-0.140004	821.8469	
	1	-2.730773	0.406614	-0.539210	921.6511	
	1	-2.486097	-1.344422	-0.674040	955.0351	
	1	-2.444675	-0.564303	0.913098	969.2934	
					981.3107	
					1006.4229	
					1044.9156	
					1061.8442	
					1154.9577	
					1213.0695	
					1240.9803	
					1294.8969	
					1321.6207	
					1339.2037	
					1352.4613	
					1401.1676	
					1457.2924	
					1460.0819	
					1467.2490	
					1498.5425	
					1499.5906	
					1725.2822	
					3038.6068	
					3055.5966	
					3074.5964	
					3115.1318	
					3127.7971	
					3130.6876	
					3147.8411	
					3148.1894	

					3243.8880	
					3769.9795	
**R30-*cis***	6	2.448940	-0.838991	-0.379828	1157.9664i	399.28297
	6	1.289356	-1.210382	0.131324	43.5062	2008.53889
	6	0.218902	-0.285182	0.632838	73.8429	2252.69085
	6	-1.085635	-0.437068	-0.156126	112.4070	
	6	-2.193170	0.475610	0.364733	124.5348	
	1	-1.422109	-1.480051	-0.112357	141.6324	
	1	-0.892507	-0.215854	-1.211441	179.6787	
	1	0.576569	0.746256	0.572653	231.6159	
	1	0.021374	-0.506127	1.688645	246.8324	
	1	3.241574	-1.479266	-0.749124	364.9203	
	1	2.685844	0.337572	-0.471775	382.2956	
	1	1.063444	-2.278362	0.167965	456.7968	
	6	-3.491702	0.324819	-0.423148	565.7033	
	1	-2.374275	0.254901	1.422396	725.2509	
	1	-1.851183	1.514940	0.319349	739.5419	
	1	-3.868188	-0.699660	-0.362159	802.8017	
	1	-4.269283	0.990835	-0.044344	852.0486	
	1	-3.334624	0.558824	-1.479196	911.5940	
	8	2.989211	1.580374	-0.181251	924.4097	
	1	3.389535	1.440159	0.690641	938.7144	
					964.2517	
					1037.8797	
					1051.4260	
					1086.6544	
					1134.5423	
					1182.2753	
					1236.9466	
					1253.1708	
					1270.3026	
					1280.0938	
					1316.2791	
					1334.4293	
					1335.3368	
					1378.5924	
					1408.5669	
					1415.5673	
					1487.0943	
					1493.4519	
					1501.7932	
					1506.5929	
					1514.4382	
					1712.5181	
					3033.7038	
					3042.4945	
					3045.5760	
					3048.3145	
					3070.4168	
					3087.2027	
					3101.3607	
					3113.8647	
					3119.1089	

					3125.4988	
					3198.5768	
					3829.7831	
**R30-*trans***	6	-2.204962	0.401936	0.541610	1194.8937i	191.24956
	6	-1.225917	0.145551	-0.306062	44.8518	2757.12560
	6	0.135483	0.784440	-0.262056	62.0248	2833.57251
	6	1.245320	-0.246989	-0.035858	72.5265	
	6	2.640032	0.373677	-0.061358	113.5795	
	1	1.178895	-1.025630	-0.804889	129.5107	
	1	1.079859	-0.745910	0.925246	198.3570	
	1	0.164812	1.543733	0.525079	228.7218	
	1	0.314841	1.298715	-1.213905	246.1525	
	1	-3.227621	-0.222455	0.434395	314.8363	
	1	-1.385288	-0.602422	-1.081943	382.2483	
	6	3.739290	-0.658631	0.176825	428.1283	
	1	2.796892	0.865456	-1.027546	554.4615	
	1	2.700075	1.159387	0.699524	734.7489	
	1	3.707547	-1.442350	-0.584474	760.5945	
	1	4.730675	-0.202641	0.147986	808.8324	
	1	3.617646	-1.137513	1.151793	864.4437	
	1	-2.200599	1.122186	1.353759	911.9597	
	8	-4.361276	-0.678967	-0.022735	924.7813	
	1	-4.562994	0.021274	-0.661758	941.2291	
					983.1529	
					1040.8158	
					1056.0656	
					1084.5824	
					1132.7674	
					1175.8206	
					1231.6919	
					1255.1920	
					1266.6301	
					1301.6470	
					1314.2374	
					1329.8139	
					1335.7590	
					1361.1216	
					1404.3117	
					1414.6608	
					1480.4975	
					1492.4384	
					1501.3921	
					1502.9488	
					1514.3071	
					1713.8466	
					3031.7949	
					3037.4338	
					3042.8605	
					3047.2403	
					3067.1648	
					3079.9450	
					3097.6889	
					3115.7658	

					3125.8158	
					3157.0420	
					3178.7688	
					3817.1943	
**R31**	6	-2.594325	-1.166896	-0.502488	917.0561i	493.19766
	6	-1.669425	-0.453656	0.116122	47.0854	1699.83702
	6	-0.295564	-0.840606	0.553252	71.6236	2043.00735
	6	0.789683	-0.010489	-0.140012	91.4642	
	6	2.198495	-0.391183	0.308230	122.0810	
	1	0.611949	1.050357	0.068710	128.7996	
	1	0.700927	-0.140246	-1.224835	180.5541	
	1	-0.147341	-1.906758	0.343414	220.2958	
	1	-0.209652	-0.707698	1.637474	248.0232	
	1	-1.938792	0.673396	0.350665	353.9824	
	6	3.272730	0.439593	-0.388715	375.3723	
	1	2.277310	-0.262482	1.393103	428.5498	
	1	2.366570	-1.455020	0.108770	599.2488	
	1	3.138471	1.503570	-0.177945	727.1432	
	1	4.274386	0.156119	-0.060380	738.9769	
	1	3.224917	0.306556	-1.472675	796.5600	
	1	-2.427304	-2.217498	-0.728765	861.8550	
	1	-3.547672	-0.738279	-0.788349	912.8655	
	8	-2.084486	2.023421	0.201482	922.4719	
	1	-1.857437	2.090037	-0.739380	949.0004	
					956.1687	
					1037.7446	
					1048.3277	
					1085.4981	
					1132.8086	
					1177.4392	
					1214.8803	
					1243.0199	
					1266.5562	
					1312.3094	
					1334.2117	
					1346.5836	
					1402.0285	
					1409.7658	
					1417.1740	
					1431.1191	
					1473.1178	
					1493.1329	
					1502.5170	
					1505.4211	
					1513.9582	
					1732.6480	
					3029.9426	
					3036.6980	
					3044.6508	
					3047.5122	
					3067.3627	
					3078.0896	
					3097.3080	

					3119.8668	
					3126.8291	
					3128.4702	
					3221.4961	
					3809.5300	
**R32**	6	-2.947708	-1.091392	0.168116	633.6835i	576.36821
	6	-1.866335	-0.562753	-0.399240	44.6364	1520.60320
	6	-0.649424	-0.124994	0.338660	56.2988	1959.89606
	1	-0.601506	1.020615	0.298884	73.2472	
	1	-3.001855	-1.245080	1.241371	102.9582	
	8	-0.715652	2.491570	-0.088091	119.0580	
	1	-1.683481	2.477261	-0.170527	142.6712	
	1	-3.807093	-1.392358	-0.419261	188.5214	
	1	-1.842433	-0.426890	-1.479560	242.1387	
	6	0.666891	-0.595103	-0.268640	340.2673	
	1	0.687129	-0.323146	-1.330349	368.5990	
	1	0.709336	-1.690654	-0.223562	468.6349	
	6	1.883571	0.003735	0.432396	595.0603	
	1	1.816704	1.095305	0.380628	691.1948	
	1	1.854162	-0.261355	1.494910	733.1721	
	6	3.198021	-0.469560	-0.181688	791.3769	
	1	4.057724	-0.026689	0.324536	912.3143	
	1	3.292273	-1.556805	-0.115234	919.1470	
	1	3.253729	-0.194578	-1.238213	944.5456	
	1	-0.719574	-0.367782	1.403472	975.7729	
					1033.0539	
					1051.2417	
					1075.1371	
					1097.2864	
					1125.4697	
					1195.9229	
					1241.2529	
					1262.6520	
					1299.9014	
					1322.3908	
					1333.6022	
					1352.4469	
					1368.3838	
					1404.8176	
					1414.6903	
					1463.4218	
					1483.3372	
					1497.8309	
					1505.3118	
					1511.2996	
					1644.1620	
					1741.1452	
					3030.4405	
					3045.5879	
					3050.8641	
					3068.6735	
					3080.5488	
					3094.7824	

					3119.8930	
					3125.6117	
					3141.6079	
					3149.9614	
					3243.1967	
					3798.9804	
**R33**	6	-2.988998	0.032337	-0.157967	731.6682i	462.14224
	6	-1.978042	-0.601792	0.428777	65.5542	1434.22954
	6	-0.714615	-1.017998	-0.265433	81.3117	1763.36825
	6	0.517445	-0.338856	0.321103	104.2091	
	6	1.821628	-0.663224	-0.385349	109.5913	
	1	0.590844	-0.501899	1.402257	151.0338	
	1	0.366543	0.808428	0.212612	174.6600	
	1	-0.590565	-2.105223	-0.175244	259.8588	
	6	3.000453	0.118610	0.187710	301.0037	
	1	2.008630	-1.741574	-0.305516	366.2665	
	1	1.711072	-0.443080	-1.451987	368.7523	
	1	3.143732	-0.111895	1.246420	464.1303	
	1	3.927561	-0.118638	-0.336860	657.0497	
	1	2.823393	1.193020	0.099333	679.8332	
	1	-2.965093	0.267889	-1.218187	722.1632	
	1	-3.875252	0.321295	0.394713	803.9922	
	1	-2.037726	-0.825643	1.492823	914.2943	
	1	-0.785353	-0.788701	-1.333311	938.1228	
	8	-0.147843	2.121996	-0.105389	955.1573	
	1	-1.082265	1.895594	0.043008	976.6986	
					1035.6161	
					1048.7906	
					1051.5263	
					1093.0069	
					1118.4483	
					1171.4724	
					1234.0741	
					1251.5316	
					1268.5900	
					1305.6034	
					1324.3449	
					1347.9362	
					1356.7724	
					1396.3741	
					1417.1390	
					1453.3788	
					1479.7858	
					1487.9460	
					1508.3144	
					1509.6369	
					1523.1670	
					1729.9091	
					3025.5072	
					3029.3152	
					3048.2520	
					3058.3953	
					3075.8569	

					3087.4220	
					3122.1203	
					3130.5487	
					3140.0854	
					3148.6206	
					3239.8782	
					3758.4313	
**R34**	6	3.462652	-0.142649	-0.213343	546.6705i	530.59905
	6	2.338224	0.152205	0.429062	43.8225	1610.46901
	6	1.004723	0.355999	-0.226339	50.1813	1990.97026
	6	-0.035838	-0.664581	0.245577	73.0311	
	6	-1.397305	-0.454852	-0.394954	117.2833	
	1	-0.137842	-0.608335	1.335301	125.0528	
	1	0.629049	1.358844	0.007803	169.6480	
	6	-2.477294	-1.404731	0.091443	177.5377	
	1	-1.730013	0.610304	-0.111041	237.1140	
	1	-1.320481	-0.457706	-1.487310	356.9944	
	1	-2.596903	-1.332063	1.174737	363.4294	
	1	-3.440979	-1.188947	-0.372345	460.3225	
	1	-2.210293	-2.438964	-0.148329	627.4584	
	1	3.482625	-0.243591	-1.294287	659.6967	
	1	4.397838	-0.289593	0.313580	767.5890	
	1	2.355030	0.244648	1.513935	845.5636	
	1	1.117835	0.293434	-1.314002	912.1040	
	1	0.318316	-1.677425	0.015808	929.9185	
	8	-2.033680	2.039122	0.187598	953.3048	
	1	-1.965716	2.368069	-0.723313	967.2513	
					1034.4532	
					1048.2670	
					1053.1744	
					1085.3707	
					1135.3799	
					1194.7363	
					1215.2181	
					1248.3431	
					1264.9115	
					1315.0720	
					1331.0025	
					1339.3679	
					1352.2967	
					1396.9237	
					1411.2202	
					1454.0157	
					1480.2294	
					1493.8229	
					1496.2967	
					1500.7415	
					1575.3463	
					1728.4461	
					3021.6535	
					3031.0565	
					3041.6491	
					3064.6001	

					3079.3113	
					3095.1660	
					3112.5246	
					3136.6633	
					3140.3361	
					3151.6535	
					3233.3088	
					3797.0569	
**R35**	6	-3.541071	0.338089	-0.453363	774.8461i	403.21543
	6	-2.415800	0.576323	0.210739	46.8213	2000.37993
	6	-1.374234	-0.454261	0.531735	59.1235	2258.24883
	6	-0.026965	-0.136619	-0.124677	81.9097	
	6	1.059743	-1.145100	0.239613	119.5911	
	1	0.302342	0.863034	0.179676	152.0644	
	1	-1.232437	-0.501717	1.618188	173.9464	
	6	2.380795	-0.849686	-0.442035	201.8183	
	1	1.200570	-1.155343	1.325038	317.6369	
	1	3.191324	-1.520396	-0.153879	359.0801	
	1	2.299011	-0.804163	-1.530194	366.1064	
	1	2.743710	0.203836	-0.090742	464.4236	
	1	-3.788818	-0.660307	-0.800715	656.3387	
	1	-4.252272	1.126823	-0.668035	725.8873	
	1	-2.200103	1.590955	0.542384	773.7505	
	1	-1.722143	-1.441174	0.209873	819.9416	
	1	-0.157800	-0.111718	-1.212743	885.8854	
	1	0.730843	-2.153718	-0.042166	937.2299	
	8	2.962486	1.582087	0.190413	954.2143	
	1	2.691078	1.934716	-0.672064	964.6829	
					1028.4742	
					1038.6665	
					1056.5423	
					1086.9327	
					1114.7470	
					1167.4479	
					1210.6383	
					1249.7195	
					1279.4346	
					1305.8781	
					1320.0802	
					1333.6062	
					1345.4712	
					1365.3625	
					1405.8726	
					1454.5166	
					1456.3746	
					1480.7302	
					1486.4366	
					1493.8339	
					1507.0785	
					1730.6854	
					3024.6487	
					3030.9958	
					3041.2703	

					3067.3875	
					3076.5624	
					3083.3802	
					3097.2884	
					3134.6063	
					3144.4705	
					3147.0437	
					3230.4118	
					3795.8367	
**R36**	6	-1.354197	-0.716096	-0.179573	878.7419i	409.34450
	6	0.045589	-0.811471	0.346574	38.1392	2108.94546
	1	-1.337117	-0.703977	-1.274127	64.4301	2395.86208
	1	-1.919309	-1.607269	0.120103	85.5010	
	6	1.148478	-0.802047	-0.404121	104.5234	
	6	2.530006	-0.858060	0.135276	164.0200	
	1	3.185704	-1.527462	-0.424673	192.9575	
	1	3.035440	0.175514	0.011578	242.6493	
	1	2.563964	-1.085422	1.200910	266.2579	
	6	-2.090264	0.524866	0.339909	312.5666	
	1	-1.538391	1.418805	0.032725	327.5179	
	1	-2.081802	0.515166	1.434893	395.4702	
	1	1.046149	-0.728092	-1.485648	534.7169	
	1	0.151015	-0.883289	1.429819	688.1923	
	8	3.223519	1.640326	-0.081975	748.8022	
	1	2.289190	1.853137	0.075839	812.0386	
	6	-3.528062	0.597830	-0.166298	882.2791	
	1	-4.098279	-0.278902	0.151744	907.7209	
	1	-4.039021	1.485700	0.210436	932.5041	
	1	-3.555001	0.633358	-1.258407	977.8996	
					1013.3108	
					1046.4064	
					1069.2858	
					1114.4596	
					1121.1864	
					1183.0899	
					1251.8760	
					1290.2994	
					1319.1826	
					1327.1093	
					1339.8737	
					1351.7519	
					1380.2745	
					1394.4262	
					1414.6028	
					1468.1481	
					1484.3824	
					1497.0279	
					1504.2331	
					1511.5210	
					1578.1839	
					1763.6657	
					3034.4371	
					3044.0277	

					3047.1177	
					3070.6490	
					3082.2660	
					3090.9624	
					3120.1968	
					3126.0953	
					3129.8037	
					3142.5153	
					3160.8319	
					3790.7120	
**R37**	6	-0.893614	-0.277653	-0.630231	880.4465i	499.37562
	6	-1.933588	-0.423359	0.486529	32.7480	1605.40863
	6	0.412059	-0.931025	-0.281268	66.7220	1965.10550
	6	1.564984	-0.303548	-0.109359	102.2921	
	6	2.900061	-0.849890	0.272159	120.0250	
	1	-1.292472	-0.733516	-1.544138	158.0091	
	1	-0.722918	0.780931	-0.846223	214.7077	
	1	2.836813	-1.930464	0.425168	228.5931	
	1	3.640712	-0.650047	-0.505512	237.2435	
	1	1.555401	0.866168	-0.285832	286.7892	
	1	0.397287	-2.015890	-0.151444	306.1687	
	6	-3.267534	0.218070	0.115728	389.4306	
	1	-2.080147	-1.485605	0.709445	524.9409	
	1	-1.538718	0.032353	1.400421	606.7438	
	1	-3.683484	-0.241488	-0.784666	743.4387	
	1	-4.000746	0.109469	0.917121	830.1995	
	1	-3.142404	1.285230	-0.084521	869.7040	
	1	3.263341	-0.390296	1.194574	890.7711	
	8	1.380947	2.203897	-0.051837	916.5412	
	1	1.025559	2.146413	0.848954	938.8453	
					1038.1438	
					1056.1180	
					1064.9305	
					1106.6291	
					1122.4381	
					1166.2416	
					1207.6599	
					1263.5485	
					1287.6686	
					1323.7756	
					1332.7984	
					1388.8412	
					1399.7110	
					1418.3983	
					1425.2345	
					1472.4746	
					1491.5044	
					1496.1771	
					1500.7979	
					1504.2076	
					1514.1142	
					1762.9100	
					3034.7039	

					3042.1759	
					3043.8648	
					3047.3661	
					3074.8924	
					3096.1012	
					3109.4801	
					3115.0631	
					3119.0041	
					3122.5896	
					3130.7239	
					3810.4540	
**R38**	6	0.867958	-0.610203	-0.545013	869.6966i	488.71365
	6	1.808746	0.146416	0.398664	37.8552	1559.94474
	6	-0.568163	-0.266344	-0.321471	77.7853	1923.71968
	6	-1.580768	-1.040408	0.036968	93.6357	
	6	-2.987830	-0.578245	0.273667	114.0446	
	1	1.142670	-0.379417	-1.580620	153.1799	
	1	0.994499	-1.691714	-0.411240	211.1124	
	1	-3.074112	0.496939	0.112642	231.6125	
	1	-3.679100	-1.089492	-0.401463	248.1916	
	1	-0.811351	0.881774	-0.475479	284.4821	
	6	3.272973	-0.210286	0.159387	301.6193	
	1	1.658192	1.221095	0.255872	372.6132	
	1	1.530674	-0.080489	1.433003	541.1298	
	1	3.567203	0.024816	-0.866779	605.8568	
	1	3.931938	0.342236	0.831545	748.9249	
	1	3.447000	-1.277358	0.320208	832.6163	
	1	-3.305455	-0.809938	1.293908	860.8688	
	1	-1.383913	-2.107740	0.154501	886.5068	
	8	-1.009630	2.206421	-0.181105	909.1348	
	1	-0.818695	2.172341	0.769537	932.7162	
					1042.9908	
					1067.0798	
					1068.5959	
					1105.3445	
					1121.3559	
					1168.1186	
					1214.9804	
					1251.7833	
					1287.0895	
					1318.0086	
					1330.6029	
					1385.3241	
					1409.9162	
					1415.6996	
					1438.9297	
					1473.0096	
					1490.9080	
					1496.3269	
					1499.3091	
					1502.5929	
					1511.9250	
					1766.1470	

					3031.8075	
					3043.1697	
					3046.6096	
					3052.2825	
					3075.2732	
					3096.7283	
					3102.5693	
					3107.4259	
					3123.4648	
					3125.6103	
					3143.2961	
					3802.8389	
**R39**	6	-2.055201	-0.244792	0.436545	606.3242i	531.43412
	6	-0.922313	-0.081413	-0.246288	43.6795	1634.45441
	6	0.442927	-0.145511	0.343646	64.1689	2029.80109
	1	0.896848	0.904822	0.329952	73.2487	
	1	-1.993449	-0.442750	1.505803	97.4480	
	8	1.330351	2.334969	-0.062621	144.4178	
	1	0.426049	2.688216	-0.034080	195.3245	
	1	-0.973795	0.111859	-1.318267	204.0087	
	6	1.432702	-1.006998	-0.433718	250.2526	
	1	1.441923	-0.676768	-1.477386	265.8587	
	1	1.075078	-2.042748	-0.432894	317.8602	
	6	2.840987	-0.927425	0.147708	396.3286	
	1	3.199751	0.104584	0.133335	530.1328	
	1	2.855028	-1.274436	1.184199	635.5287	
	1	0.407550	-0.423448	1.401974	720.3089	
	1	3.541198	-1.541081	-0.421724	793.4115	
	6	-3.429563	-0.187797	-0.157161	868.5967	
	1	-4.028918	0.593005	0.319563	918.7127	
	1	-3.388510	0.015190	-1.228423	938.5576	
	1	-3.958788	-1.132583	-0.005479	1005.9198	
					1049.8987	
					1069.9539	
					1080.8467	
					1106.1773	
					1123.4959	
					1183.1313	
					1248.7075	
					1281.8045	
					1303.9413	
					1330.5135	
					1341.3636	
					1363.5942	
					1404.1690	
					1414.7712	
					1418.5781	
					1484.9705	
					1485.7570	
					1496.7596	
					1506.4062	
					1508.9049	
					1691.8655	

					1798.4355	
					3041.7151	
					3043.9406	
					3049.2350	
					3076.9358	
					3092.0970	
					3100.5234	
					3123.4624	
					3124.4441	
					3126.1287	
					3130.2015	
					3142.4067	
					3787.6151	
**R40**	6	-0.512926	-0.992165	-0.267042	748.6961i	456.87916
	6	-1.589908	-0.170222	0.432643	56.0233	1520.22072
	6	0.859829	-0.737120	0.283699	70.4528	1848.64444
	6	1.879599	-0.243622	-0.416538	98.4574	
	6	3.248969	0.024034	0.132668	128.0166	
	1	-0.529332	-0.774516	-1.339869	151.8443	
	1	3.302055	-0.220156	1.194922	191.7403	
	1	4.001665	-0.568324	-0.394558	213.6573	
	6	-2.986383	-0.375189	-0.123682	289.1617	
	1	-1.553805	-0.310037	1.517366	310.3325	
	1	-1.319093	0.950878	0.278381	337.1125	
	1	-3.298733	-1.417055	0.002404	391.9199	
	1	-3.714609	0.259194	0.383121	538.3703	
	1	-3.015510	-0.142300	-1.190591	670.4725	
	1	3.525669	1.074347	0.006065	737.1501	
	1	1.724535	-0.023097	-1.472548	790.7001	
	1	1.007819	-0.962696	1.340210	891.7061	
	1	-0.761443	-2.055810	-0.152693	916.6965	
	8	-0.628819	2.170013	-0.054502	932.6240	
	1	0.266253	1.795170	0.013322	1013.7146	
					1032.4350	
					1070.6778	
					1077.1049	
					1087.2467	
					1110.3523	
					1167.6043	
					1190.0863	
					1245.2793	
					1286.8586	
					1335.5444	
					1337.7306	
					1355.8149	
					1386.9166	
					1409.9361	
					1416.0286	
					1476.4945	
					1484.3383	
					1492.7087	
					1495.0023	
					1497.4124	

					1554.7843	
					1753.5825	
					3018.6786	
					3036.3580	
					3040.3224	
					3072.5593	
					3088.1370	
					3098.9227	
					3108.3768	
					3125.0382	
					3128.4037	
					3135.1694	
					3141.6656	
					3771.8952	
**R41**	6	-2.333288	1.061108	0.171002	773.5017i	305.14317
	6	-0.922818	0.958976	-0.373481	38.0491	2253.89974
	1	-2.887255	0.058842	-0.058703	51.6056	2425.46418
	6	-0.111577	-0.151621	0.302943	83.9247	
	1	-0.956462	0.780968	-1.452751	135.4271	
	1	-0.407527	1.916317	-0.230356	147.0158	
	6	1.272026	-0.275361	-0.263537	162.6256	
	1	-0.052379	0.040905	1.379717	205.5395	
	1	-0.650522	-1.096798	0.168739	289.7097	
	6	2.396460	-0.073838	0.417017	313.7522	
	1	2.326495	0.189019	1.472048	320.0972	
	1	1.336042	-0.537979	-1.319871	392.9039	
	8	-3.379858	-1.273621	-0.169090	540.5306	
	1	-3.259434	-1.535983	0.757392	727.8042	
	1	-2.943879	1.821049	-0.318487	784.3866	
	1	-2.365201	1.194709	1.254603	795.1844	
	6	3.778080	-0.185907	-0.154759	869.1596	
	1	3.744965	-0.447171	-1.213868	907.2697	
	1	4.323200	0.756170	-0.049967	934.3650	
	1	4.357524	-0.951229	0.369112	1004.5186	
					1048.3200	
					1059.4692	
					1071.7020	
					1090.0080	
					1108.9341	
					1174.6615	
					1194.9954	
					1249.1643	
					1287.2335	
					1313.8600	
					1336.2784	
					1342.8114	
					1347.8520	
					1392.8829	
					1417.8907	
					1451.8666	
					1477.6622	
					1485.4513	
					1487.5717	

					1495.0295	
					1499.3737	
					1762.3552	
					3027.1272	
					3038.3645	
					3043.3851	
					3066.4508	
					3076.0648	
					3090.2895	
					3101.2764	
					3124.6769	
					3128.9283	
					3141.5541	
					3145.9945	
					3791.7862	
**R42**	6	-2.614072	-0.590672	-0.233466	983.7352i	428.84709
	6	-1.346594	-1.201887	0.343866	47.0632	1696.50812
	1	-2.578982	0.578002	-0.056280	73.4880	1991.38993
	1	-2.701785	-0.714725	-1.314209	114.9537	
	1	-3.524612	-0.918384	0.269498	128.5617	
	6	-0.109562	-0.690016	-0.337197	164.3840	
	1	-1.289748	-0.995147	1.415942	211.3847	
	1	-1.398634	-2.292095	0.229968	290.5661	
	6	0.860830	-0.005505	0.265640	306.9071	
	1	-0.030144	-0.885875	-1.407057	330.7139	
	1	0.778124	0.183386	1.337199	375.0125	
	8	-2.114107	1.858347	0.160283	477.9697	
	1	-1.176486	1.668010	-0.014097	485.6144	
	6	2.102532	0.507976	-0.403604	694.1434	
	1	2.051101	0.301284	-1.476086	772.2548	
	1	2.152713	1.596539	-0.289939	824.4039	
	6	3.368362	-0.114440	0.194199	886.2860	
	1	3.431094	0.087090	1.266343	912.3421	
	1	3.365184	-1.197618	0.056754	917.4077	
	1	4.266056	0.290028	-0.276929	984.2616	
					1015.8320	
					1036.7082	
					1055.7316	
					1098.1606	
					1128.0124	
					1181.2059	
					1207.1050	
					1252.3038	
					1280.1726	
					1317.7552	
					1320.1768	
					1335.3468	
					1349.7596	
					1389.7737	
					1409.9672	
					1461.6433	
					1478.6477	
					1483.1099	

					1487.0895	
					1503.2561	
					1510.0731	
					1757.6237	
					3032.3489	
					3043.5162	
					3048.1279	
					3080.4127	
					3086.6213	
					3091.4992	
					3117.7727	
					3123.8700	
					3126.9874	
					3131.8742	
					3155.8493	
					3776.0965	
**R43**	6	0.976942	-0.502128	0.373579	630.2338i	533.87150
	6	-0.195515	-0.431628	-0.256879	40.0808	1696.68303
	6	-1.520524	-0.339854	0.417947	66.7355	2104.69700
	1	0.985963	-0.495175	1.464167	80.4945	
	8	-2.054377	2.189078	-0.126090	123.3137	
	1	-1.095600	2.343808	-0.116688	140.6865	
	1	-1.929905	0.719479	0.252568	173.7199	
	6	2.315502	-0.578348	-0.298500	192.7605	
	1	2.814606	-1.507290	-0.001712	234.0543	
	1	2.177690	-0.622770	-1.382249	305.1426	
	1	-0.211140	-0.438588	-1.347236	341.5097	
	6	-2.570746	-1.291002	-0.142901	466.3433	
	1	-3.536522	-1.141935	0.341670	486.1169	
	1	-2.700715	-1.131737	-1.215631	638.2688	
	1	-2.263557	-2.329052	0.011013	752.1332	
	6	3.211041	0.608450	0.072437	794.8911	
	1	4.195368	0.518063	-0.390901	868.1870	
	1	2.762412	1.548138	-0.256963	918.3480	
	1	3.352532	0.665760	1.154548	926.9840	
	1	-1.416306	-0.434268	1.502036	1006.5395	
					1030.5194	
					1046.1753	
					1084.2672	
					1097.5402	
					1125.9846	
					1192.2737	
					1241.1272	
					1267.2923	
					1298.4913	
					1331.4410	
					1339.6257	
					1370.0115	
					1400.5161	
					1411.9559	
					1412.8231	
					1487.0926	
					1498.0675	

					1501.2479	
					1504.4281	
					1508.4171	
					1672.8553	
					1779.5194	
					3040.1260	
					3046.0231	
					3050.0454	
					3088.7875	
					3091.8781	
					3117.9019	
					3119.0011	
					3124.2677	
					3127.5142	
					3133.4556	
					3143.9227	
					3792.5234	
**R44**	6	-0.656348	-0.826630	0.155230	832.2583i	528.30341
	6	0.483614	-0.198692	-0.087908	42.6875	1482.90017
	6	1.786303	-0.724682	-0.597247	53.7268	1836.53008
	6	-1.918596	-0.179877	0.649883	93.6743	
	6	2.934974	-0.444666	0.374908	118.4398	
	6	-3.055468	-0.296180	-0.369991	163.4984	
	1	-0.695860	-1.903817	-0.024326	192.7394	
	1	0.510890	0.954867	0.153355	231.2831	
	1	1.682640	-1.800613	-0.775745	248.7116	
	1	-2.223197	-0.662550	1.584370	290.6632	
	1	-1.716770	0.869472	0.877961	343.5337	
	1	3.028446	0.628925	0.552711	471.9738	
	1	2.753151	-0.931272	1.335067	477.9117	
	1	-3.975299	0.146476	0.016818	636.0042	
	1	-2.793386	0.215198	-1.298892	769.6836	
	1	-3.260731	-1.342287	-0.611124	794.2796	
	1	3.883059	-0.810271	-0.023717	876.1529	
	1	2.008015	-0.260178	-1.564025	878.2331	
	8	0.413470	2.323683	-0.008288	909.1953	
	1	0.044411	2.330942	-0.905404	926.8729	
					1027.4127	
					1046.8415	
					1090.5741	
					1096.4640	
					1123.3213	
					1178.4396	
					1210.9086	
					1260.1867	
					1294.3788	
					1310.6050	
					1336.0086	
					1375.2197	
					1410.2217	
					1412.2378	
					1430.6952	
					1473.5110	

					1494.8135	
					1503.0635	
					1505.5362	
					1511.0700	
					1512.7117	
					1757.9436	
					3036.2623	
					3047.1036	
					3051.1695	
					3056.7521	
					3077.3265	
					3093.4056	
					3110.7716	
					3129.7737	
					3132.7198	
					3133.9949	
					3143.0545	
					3805.0431	
**R45-*cis***	6	-0.372738	0.241484	-0.359172	1204.0369i	292.52149
	6	1.009110	0.821868	-0.166558	48.1441	1174.45193
	6	1.927398	-0.186224	0.471317	81.6846	1362.29903
	6	-1.464521	0.711045	0.214444	133.9799	
	6	2.995531	-0.708186	-0.119589	178.9489	
	1	-0.440340	-0.626378	-1.012358	190.9430	
	1	0.941690	1.717744	0.457590	320.2024	
	1	1.414984	1.120671	-1.137888	392.0415	
	1	1.657072	-0.502933	1.476210	466.2665	
	1	-2.517025	0.177392	-0.038184	556.4435	
	1	3.617595	-1.441113	0.379828	668.8284	
	1	3.284451	-0.414316	-1.123945	724.0946	
	8	-3.476795	-0.694140	-0.092453	838.2231	
	1	-3.173274	-1.306474	0.594854	901.9708	
	1	-1.539478	1.548594	0.900865	924.3587	
					959.3702	
					973.0030	
					979.2499	
					1037.6640	
					1075.6395	
					1163.2231	
					1236.6358	
					1263.1907	
					1271.1271	
					1298.9714	
					1326.5250	
					1358.7546	
					1453.9923	
					1485.0435	
					1707.3957	
					1730.9657	
					3050.7681	
					3101.6746	
					3122.0961	
					3146.6591	

					3155.5754	
					3199.8249	
					3243.6691	
					3821.6318	
**R45-*trans***	6	-0.372738	0.241484	-0.359172	1293.7437i	189.37813
	6	1.009110	0.821868	-0.166558	51.3578	1595.29177
	6	1.927398	-0.186224	0.471317	86.1411	1693.89052
	6	-1.464521	0.711045	0.214444	100.6111	
	6	2.995531	-0.708186	-0.119589	117.8236	
	1	-0.440340	-0.626378	-1.012358	229.9437	
	1	0.941690	1.717744	0.457590	301.3195	
	1	1.414984	1.120671	-1.137888	346.6467	
	1	1.657072	-0.502933	1.476210	433.8078	
	1	-2.517025	0.177392	-0.038184	524.7188	
	1	3.617595	-1.441113	0.379828	657.7926	
	1	3.284451	-0.414316	-1.123945	784.9248	
	8	-3.476795	-0.694140	-0.092453	852.1882	
	1	-3.173274	-1.306474	0.594854	894.3490	
	1	-1.539478	1.548594	0.900865	908.4597	
					968.6880	
					973.2723	
					982.2808	
					1035.3196	
					1073.8622	
					1153.2390	
					1224.2563	
					1262.8458	
					1290.2479	
					1301.8706	
					1327.3009	
					1347.2767	
					1450.5155	
					1476.4146	
					1704.8359	
					1728.0640	
					3049.4613	
					3098.5272	
					3143.6846	
					3156.4867	
					3159.0844	
					3185.2445	
					3236.2967	
					3818.0046	
**R46**	6	-1.101688	-0.198292	0.141981	1085.5569i	454.07208
	6	0.115527	-0.966924	0.558255	74.0638	764.45031
	6	1.254798	-0.717743	-0.394886	89.8254	1119.72923
	6	-2.275106	-0.635052	-0.276434	123.0899	
	6	2.365745	-0.061302	-0.077141	172.2807	
	1	-0.936538	0.983963	0.182637	259.9488	
	1	-0.133723	-2.034120	0.577566	343.5218	
	1	0.411686	-0.668965	1.568261	360.6928	
	1	1.114145	-1.084683	-1.409111	445.2827	
	1	3.151392	0.105954	-0.804512	592.1557	

	1	2.526874	0.319112	0.927161	658.9383	
	8	-0.397315	2.196981	0.062715	694.1558	
	1	0.438992	1.933700	-0.356132	877.4874	
	1	-2.488496	-1.700956	-0.319595	896.6615	
	1	-3.061471	0.046023	-0.578653	927.7584	
					950.8508	
					977.4776	
					981.9760	
					1018.0178	
					1041.8740	
					1100.4325	
					1166.4549	
					1258.3734	
					1301.1926	
					1329.0741	
					1426.5592	
					1448.5027	
					1451.4437	
					1472.0863	
					1721.2032	
					1737.1760	
					3047.4290	
					3100.0309	
					3124.4351	
					3145.6031	
					3159.7610	
					3223.3666	
					3241.3684	
					3773.7096	
**R47**	6	-1.356262	-0.769397	0.343886	643.2651i	546.12307
	6	0.017132	-0.200239	0.509954	57.6148	824.84364
	6	0.344271	0.987153	-0.329111	70.1640	1255.53760
	6	-2.339422	-0.203604	-0.350489	100.4166	
	6	0.868939	2.115606	0.139435	134.9910	
	1	-1.534113	-1.716107	0.846676	216.1256	
	1	0.743160	-1.043624	0.210852	255.0100	
	1	0.248424	-0.024948	1.565245	438.8369	
	1	0.152155	0.884318	-1.395178	442.0531	
	1	-3.313508	-0.671995	-0.422200	544.2560	
	1	-2.208543	0.748474	-0.853305	649.4752	
	1	1.110268	2.943558	-0.516472	667.9735	
	1	1.066854	2.248454	1.198461	902.1456	
	8	2.001086	-1.750609	-0.264073	924.7733	
	1	2.518659	-0.940373	-0.403548	966.3824	
					973.1891	
					980.7173	
					1006.8019	
					1041.5228	
					1046.8243	
					1158.4396	
					1200.8681	
					1278.2557	
					1324.6559	

	1332.3244	
	1362.2006	
	1450.7186	
	1467.5320	
	1681.1492	
	1713.1286	
	1788.7935	
	3095.5824	
	3145.1802	
	3145.9415	
	3155.7432	
	3160.5889	
	3238.6223	
	3239.9613	
	3782.9632	
